# A revision of the genus *Ptochoryctis* Meyrick, 1894 (Lepidoptera, Gelechioidea, Xyloryctidae) with the description of 12 new species

**DOI:** 10.3897/zookeys.1285.185098

**Published:** 2026-07-22

**Authors:** Mark J. Sterling, David C. Lees

**Affiliations:** 1 Department of Science, Natural History Museum, Cromwell Road, South Kensington, London SW7 5BD, UK Department of Science, Natural History Museum London United Kingdom

**Keywords:** Autostichidae, Depressariidae, DNA barcodes, generic revision, Lecithoceridae museomics, new species

## Abstract

The genus *Ptochoryctis* Meyrick, 1894 is revised and reinstated in the Xyloryctidae (previously treated in Autostichidae) based on molecular and morphological evidence. A fuller description is provided of the morphology of all the species previously described in *Ptochoryctis* which are not combined elsewhere. The following new species of *Ptochoryctis* are described: *Ptochoryctis blanchella***sp. nov**., *P. caputanatis***sp. nov**., *P. draconella***sp. nov**., *P. flavalbella***sp. nov**., *P. fuscilinea***sp. nov**., *P. kitchingi***sp. nov**., *P. marmorella***sp. nov**., *P. minimella***sp. nov**., *P. persicotincta***sp. nov**., *P. ochraceella***sp. nov**., *P. robinsoni***sp. nov**. and *P. splendidella***sp. nov**. *Deloryctis* Meyrick, 1934 is synonymised with *Ptochoryctis*. The following new combinations are established: *Ptochoryctis corticivora* (Meyrick, 1934), **comb**. **nov**. (which is consequently transferred from Depressariidae to Xyloryctidae), *Metathrinca alma* (Meyrick, 1908), **comb**. **nov**., *M. inviolata* (Meyrick, 1925), **comb**. **nov**., *M. ochrograpta* (Meyrick, 1923), **comb**. **nov**.; and *M. perigramma* (Meyrick, 1926), **comb. no**v.

## Introduction

This is the second part of a revision of the genera *Athrypsiastis* Meyrick, 1910, *Deloryctis* Meyrick, 1934, *Linoclostis* Meyrick, 1908, *Metathrinca* Meyrick, 1908, *Ptochoryctis* Meyrick, 1894, and *Topiris* Walker, 1863, almost all of which occur naturally in the Old World Tropics. The first part covers the genera *Athrypsiastis* and *Topiris* (Sterling et al. 2025). We had intended to cover all the remaining genera in a single work. However, the current concepts of *Metathrinca* and *Linoclostis* make little sense in the context of their included described species and there are a very large number of undescribed species which would currently fall within these concepts. By contrast, *Ptochoryctis* form a comparatively small and well-defined group. In addition, there is no historical issue with the placement of *Metathrinca* and *Linoclostis* within the Xyloryctidae, whereas the family placement of *Ptochoryctis* has been more controversial (Sterling and Singh 2025). For these reasons we have decided to publish a standalone revision of the genus *Ptochoryctis*, which also requires an examination of the monobasic genus *Deloryctis* Meyrick, 1934, originally described in Xyloryctidae but subsequently placed in Depressariidae (Hodges 1978).

The genus *Ptochoryctis* was established by Edward Meyrick for *Ptochoryctis eremopa* Meyrick, 1894. During his lifetime Meyrick placed a further 17 species of (principally) white Asian gelechioid species within the genus, including *Ptochoryctis ancistrias* Meyrick, 1906, which he subsequently (Meyrick 1908) combined as *Metathrinca ancistrias* (Meyrick, 1906) and which is the type species of that genus. Apart from *Ptochoryctis sundarbanica* Sterling & Singh, 2025, which was described for the purpose of including data from this Indian species within this paper in accordance with applicable legislation implementing the Convention on Biological Diversity, the only author other than Meyrick ever to describe a species in *Ptochoryctis* is William Kearfott, who described *Ptochoryctis tsugensis* Kearfott, 1910 with the help of Meyrick (now combined as *Metathrinca tsugensis* (Kearfott, 1910): Okada 1962). Apart from *P. sundarbanica*, no species of *Ptochoryctis* has been described since 1938, when *Ptochoryctis intacta* Meyrick, 1938, combined as *Metathrinca intacta* (Meyrick, 1938) (Clarke 1955b: 446), was described.

Meyrick’s original description of *Ptochoryctis* is as follows (Meyrick 1894: 19): “Head with appressed scales, sidetufts loosely spreading; ocelli present; tongue developed. Antennae ¾, in • ♂ bipectinated, towards apex simple, basal joint stout, without pecten. Labial palpi long, curved, ascending, with appressed scales, terminal joint shorter than second, acute. Maxillary palpi rudimentary. Posterior tibiae clothed with long hairs. Forewings with vein 1b furcate, 2 from 4/5ths, 7 and 8 stalked, 7 to hindmargin, 9 absent, 11 from beyond middle. Hindwings 1, trapezoidal-ovate, hindmargin sinuate, cilia ½; veins 3 and 4 short-stalked, 6 and 7 approximated towards base. Nearly allied to *Cryptophasa*”.

Although this description is reasonably detailed, almost all the characters to which Meyrick refers are also present in the currently described species of *Metathrinca* and *Linoclostis*. The exception is “ocelli present”. This is an error. In no described species of *Ptochoryctis*, including those described here (nor *Metathrinca* or *Linoclostis*), are ocelli present.

On Meyrick’s generic descriptions, *Metathrinca* can be distinguished from *Ptochoryctis* on the basis of the absence of vein 4 (M_3_ in modern notation) in the forewing. This distinction is, however, currently unsatisfactory as, following the expansion of the concept of *Metathrinca* by Diakonoff (1967), all species currently placed in *Metathrinca*, other than *M. ancistrias*, *M. ophiura*Meyrick 1908, and *M. memnon* Meyrick, 1914, have three rather than two medial veins in the forewing (in other words there is no absent medial forewing vein in these taxa). Likewise, *Linoclostis* can be distinguished from *Ptochoryctis* on the basis of the absence of 6 in the forewing (M_1_ in modern notation). This distinction is helpful as far as it goes, but *Linoclostis* is currently a small and poorly known genus and the use of venation alone to distinguish genera is now often not satisfactory.

A number of species were transferred from *Ptochoryctis* to *Metathrinca* by Clarke (1955b) but the reasons for the transfer were not expressed and the concept of *Ptochoryctis* remains unclear.

*Ptochoryctis* was originally placed by Meyrick in his recently established family, the Xyloryctidae. It was included in the “Cryptophasidae” by Fletcher (Fletcher 1929). It was transferred to the Oecophoridae: Autostichinae by Hodges (Hodges 1978: 8). Autostichidae now has family status and currently contains six subfamilies. On a superficial basis the described species of *Ptochoryctis* appear to have much more in common with the genera *Metathrinca*, *Linoclostis*, *Topiris* and *Athrypsiastis* than with any subfamily within the Autostichidae.

Our aims in this paper are to revise the genus *Ptochoryctis* and to consider its family placement on the basis of both molecular and morphological data. We expanded our study to include the monobasic genus *Deloryctis* (Meyrick, 1934: 464). It was noted by Bradley (1959) that *Deloryctis corticivora* Meyrick, 1934 was superficially similar to *Ptochoryctis chalazopa* Meyrick, 1920, that the larva of both species had been found feeding on the bark of Rubber (*Hevea brasiliensis* (Euphorbiaceae)), and that there was an overall similarity between the female genitalia of these two species.

Following our new descriptions and combinations, *Ptochoryctis* consists of 21 described species, all of which occur in the Oriental Tropics.

As noted above, the genus and almost all the previously described species of *Ptochoryctis* were described by Edward Meyrick. Meyrick was a classics scholar. He read classics at Cambridge University and in his professional life he was the classics master at Malborough School, a prestigious English public school. He evidently enjoyed ascribing suitable scientific names to the estimated 20,000 species (Hill, 1939) he described. In addition to explaining the etymology for the newly described species in this paper, with the help of Sir Anthony Galsworthy, a modern classics scholar, we have sought to explore the etymology of Meyrick’s scientific names for the taxa he described. We provide a brief note for each relevant name.

## Materials and methods

The abbreviations used in this paper are as follows:

**BIN** Barcode Index Number.

**BOLD** Barcode of Life Database (www.barcodinglife.org).

**NHMUK** Natural History Museum (formerly British Museum [Natural History]), London, UK.

**ZSI** Zoological Survey of India, Kolkata, India.

**NZCZSI** National Zoological Collection of the Zoological Survey of India.

The specimens examined for this paper, including all type material, are held at the NHMUK unless otherwise expressly stated. All types are adults which were deposited in the main NHMUK collection on or prior to 24 January 2024, with the exception of the types of *P. sundarbanica* which are held by the ZSI in the NZCZSI in Kolkata.

All types of *Ptochoryctis* and *Deloryctis* were examined with the exception of the type of *Ptochoryctis perigramma* (Meyrick, 1926), which was deposited in the Sarawak Museum but is lost (Robinson 2004).

External examination of materials was carried out using a Nikon SMZ800 stereomicroscope. The illustrated material was photographed using a Canon EOS 5DSR camera and MP-E 65 mm lens equipped with a Stack shot system operated by Helicon Remote software (v. 3.8.4 W); the images were combined with Helicon Focus software (v. 6.7.1). Genitalia dissection and mounting followed Robinson (1976). All genitalia preparations were also made using the same Nikon SMZ800 stereomicroscope. Descriptions of the genitalia follow Klots (1970) and Kristensen (2003). Descriptions of wing venation follow the Comstock-Needham method as summarised in Miller (1970) with the modifications adopted in Common (1994: 14). Venation preparations were made following the method described in Sterling et al. 2025.

### DNA sequencing

Specimens sent to BOLD for Sanger sequencing with failure tracking (see below) were sorted by locality and physical appearance and pre-1960 specimens excluded. The selected specimens were sampled by removing one or two legs, where possible from the left-hand side of a specimen, and placing them in an individual well containing three drops of absolute ethanol to minimise electrostatic movement. Care was taken to wipe forceps with tissue paper between each handling to minimise risk of cross contamination. Plates were then sent to BOLD for DNA sequencing following standardised procedures described in Ratnasingham and Hebert (2007) to try to assemble bidirectionally a full length 658 bp DNA barcode of COI-5P using the Folmer-modified primers LepF1 and LepR1). In many cases, because of the age of the specimens, this was done using primer pairs amplifying around or just >1/2 length (overlapping fragments ~ 307–407 bp using primer pairs MlepF1/LepR1 and LepF1/MlepR2). We refer here to this procedure as ‘failure tracking’. In cases where only one of these fragments was sequenced, they were not enough to generate a Barcode Index Number (BIN) but more than enough to identify the species to a known sequence (Hajibabaei et al. 2006; Meusnier et al. 2008).

Two specimens were also sent to BOLD for next generation sequencing (Shokralla et al. 2014). Genome skimming was used for two specimens to obtain DNA barcodes using the methodology set out in Sterling et al. (2025: 301).

### Molecular analysis and taxon sampling

**Figure 1.**
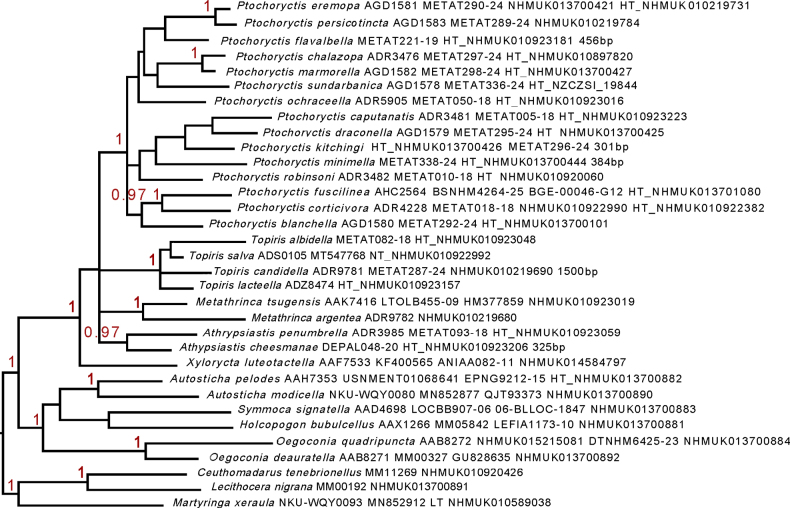
Phylogeny based on 6081 bp (COI + 3-7 nuclear genes indicated in Suppl. material 1; number of different genes is given by #g at end of label), for 32 taxa (the 14 taxa of *Ptochoryctis* here exclude the unstable *P. minimella*) based on an ML Analysis in IQTree. Support values show SHaRLT/ ABayes/ UHboot (1000 reps) measures, respectively, where significant or closely submarginal. Different families as well as *Ptochoryctis* are delineated by coloured vertical bars. Exemplars illustrated as per labelled branches. The tree is midpoint rooted on Lecithoceridae.

Variable length DNA barcode sequences obtained from BOLD or Genome Skimming were aligned using MAFFT online (https://mafft.cbrc.jp/alignment/) and checked by eye in Bioedit v. 7.7.1 (Hall 1999). Other molecular data from GenBank (see Suppl. material 1) were aligned in the same way and concatenated in MS Excel, inserting minimal gaps to allow translation (thus including 9 positions with gaps/ambiguous data only, and 847 parsimony-informative sites) and to facilitate checking by eye in Bioedit v. 7.7.1 (Hall 1999). These resulted in a matrix alignment of 6081 nucleotides, with COI-5P common to all taxa, COI-3P for nine taxa and further scaffolded by 3–7 nuclear genes for all sampled families where publicly available. The nuclear genes (their number indicated in Suppl. material 1 and Fig. 1) comprised EF-1 α in two fragments as well as Wingless, RpS5, CAD, MDH, GAPDH, and IDH for eight outgroup taxa (*Martyringa xeraula* (Meyrick, 1910), *Ceuthomadarus tenebrionellus* Mann, 1864, *Lecithocera nigrana* (Duponchel, 1835) [Lecithoceridae]; *Autosticha modicella* (Christoph, 1882), *Symmoca signatella* Herrich-Schäffer, 1854, *Oegoconia deauratella* (Herrich-Schäffer, 1854), *Holcopogon bubulcellus* (Staudinger, 1859) [Autostichidae]; and *Metathrinca tsugensis* [Xyloryctidae]). COI-5P was available for all 32 taxa and COI-3P for the outgroups *M. xeraula*, *H. bubulcellus*, *S. signatella*, *O. quadripuncta* (Haworth, 1828), *M. tsugensis*, *Topiris salva* and *T. candidella*. COI-5P data was included for *Autosticha pelodes* (Meyrick, 1883) and *Oegoconia quadripuncta* because they are each a representative of the type species of their genera and potentially to link them with COI data for congenerics. The type species of *Symmoca* Hübner, 1825 is *Tinea signella* Hübner, 1796. We used *S. signatella* because it is clearly congeneric and specimens and preparations were readily available. In all, 14 species of the ingroup taxon *Ptochoryctis* were included in the molecular analysis, including the most complete available sequence for their Barcode Index Number, where allocated. However, in the case of three species, the only sequence obtained was too short for a BIN number to be allocated. We included the partial sequences for two of these species in the molecular analysis. However, the partial sequence of *P. minimella* sp. nov. was not included in the molecular only analysis as it proved unstable in all tree runs.

Suppl. material 1 details the accession numbers and other available details for the sequenced taxa (including for *P. minimella* which was used only in our combined analyses). DNA barcodes are accessible in the following BOLD dataset https://v4.boldsystems.org/index.php/MAS_Management_DataConsole?codes=DS-PTOCHO. Details of the sequences used in our molecular analysis are contained in Suppl. material 1.

During ‘Neighbor Joining’ searches on BOLD using the tree view and image building options, we discovered another sequence belonging to *Ptochoryctis*, BINBOLD:ADJ0350, Process ID INFCO10279-17. We did not physically examine this specimen, but we include a short note on it in the morphological systematics section.

**Figure 2.**
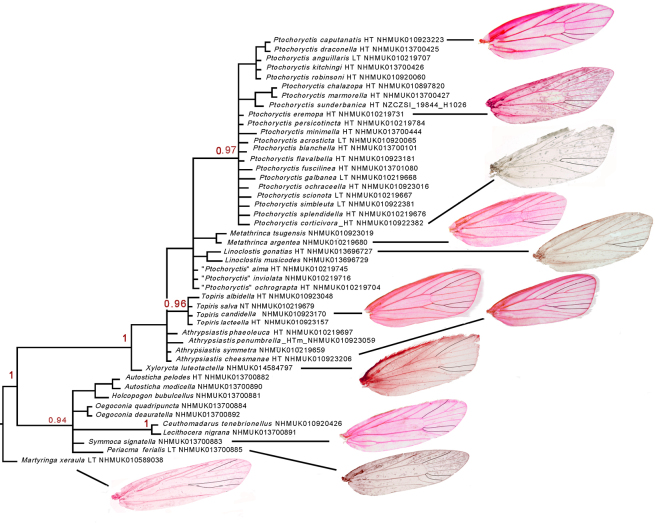
Phylogeny based on a consensus tree derived from 40 morphological characters for 47 taxa, from a Bayesian analysis in MrBayes. Posterior support values where significant are indicated. The tree is rooted on *Martyringa xeraula*. Forewing venation, an important diagnostic character, is indicated for exemplar taxa; R_3_ (where present), M_3_ + CuA_1_ are outlined in black.

**Figure 3.**
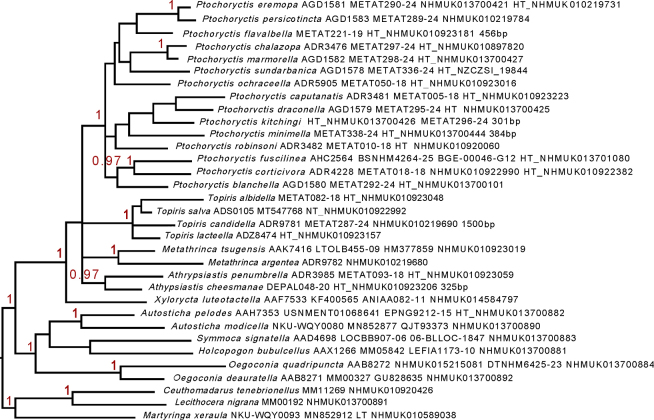
Phylogeny based on a common dataset of molecular and morphological data (6121 characters, 40 morphological) from a consensus tree analysed in MrBayes for 33 taxa (including *Ptochoryctis minimella*). Posterior support values for nodes are shown where significant. The tree is midpoint rooted on Lecithoceridae.

**Figure 4.**
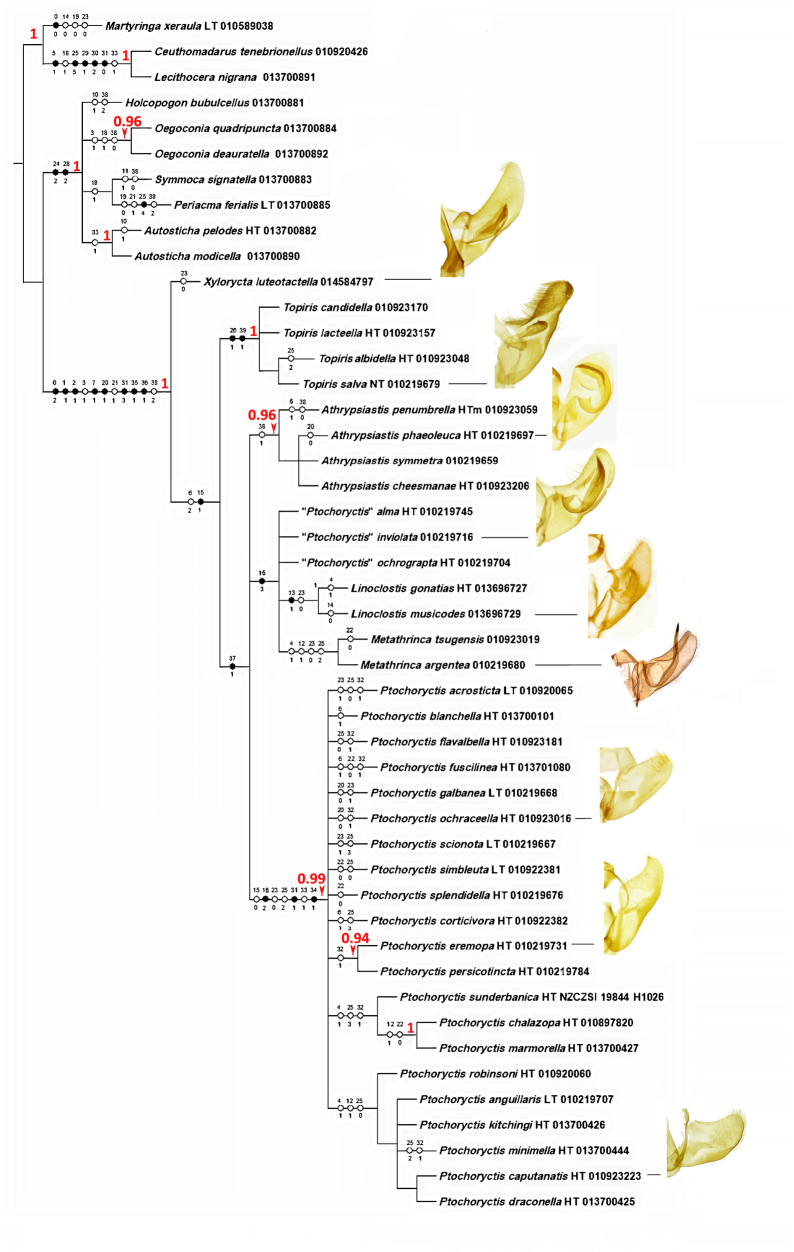
Phylogeny based on a mixed dataset for 47 taxa and 6121 characters (including 40 morphological characters) from a consensus tree analysed in MrBayes with posterior support values for nodes shown where significant. The combined matrix and this consensus tree were loaded into Winclada to allow the character mapping: filled circles show forward nonhomoplasious changes, which may be either synapomorphies or autapomorphies; unfilled circles show forward changes with homoplasy or reversal. Valvae of xyloryctid exemplars illustrate presence or absence (*Ptochoryctis* only) of a developed articulated saccular process on the male valva. The tree is midpoint rooted on Lecithoceridae.

The 6081 bp matrix was run in IQTree online (http://iqtree.cibiv.univie.ac.at/) under a GTR+F+I+G4 model, as automatically selected under BIC, with support values as SH-ARLT, ABayes and UFboot (run 1000 times), and all options otherwise as default. Further analyses (outputs shown in Figs 2, 3, 4) were run in MrBayes v. 3.2.7, where Bayesian support is displayed where over 0.94 from the consensus trees.

Bioedit in conservation plot mode against a reference sequence (following the same procedure as in Sterling et al. 2023) was used to check possible shared characters in the amino acids of the DNA barcodes, which are numbered from the first complete codon, after first ordering the species ordered according to molecular phylogenetic results (below).

Mega X (Kumar et al. 2018) was used with default options (including pairwise deletion of missing data) under the invertebrate mt code to produce a pairwise distance matrix for DNA barcodes for the 12 species that included full BINs (Table 1).

**Table 1.** Pairwise distances between the 12 species of *Ptochoryctis* for which full DNA barcodes (658 bp) are available with their BINs. Pairwise distances are highlighted in red for the two most closely related species pairs.

	***P. eremopa*BOLD:AGD1581METAT290-24 013700421**	***P. persicotincta* HT BOLD:AGD1583METAT289-24**	***P. caputanatis* HT M BOLD:ADR3481METAT005-18 010923223**	***P. draconella* HT M BOLD:AGD1579METAT295-24**	***P. robinsoni* HT M BOLD:ADR3482METAT010-18**	***P. chalazopa* HT M BOLD:ADR3476METAT297-24**	***P. marmorella* HT M BOLD:AGD1582METAT298-24**	***P. blanchella* HT M BOLD:AGD1580METAT292-24**	***P. sundarbanica* HT M BOLD:AGD1578METAT336-24**	***P. ochraceella* HT M BOLD:ADR5905METAT050-18**	***P. corticivora* M BOLD:ADR4228METAT018-18 010922990**	***P. fuscilinea*BOLD:AHC2564BSNHM1690-25 BGE 00019 F11**
***P. eremopa*BOLD:AGD1581METAT290-24 013700421**												
***P. persicotincta* HT BOLD:AGD1583METAT289-24**	0.029											
***P. caputanatis* HT M BOLD:ADR3481METAT005-18 010923223**	0.103	0.100										
***P. draconella* HT M BOLD:AGD1579METAT295-24**	0.105	0.103	0.073									
***P. robinsoni* HT M BOLD:ADR3482METAT010-18**	0.102	0.103	0.100	0.103								
***P. chalazopa* HT M BOLD:ADR3476METAT297-24**	0.090	0.094	0.100	0.091	0.090							
***P. marmorella* HT M BOLD:AGD1582METAT298-24**	0.087	0.091	0.099	0.085	0.090	0.027						
***P. blanchella* HT M BOLD:AGD1580METAT292-24**	0.105	0.097	0.105	0.105	0.093	0.094	0.090					
***P. sundarbanica* HT M BOLD:AGD1578METAT336-24**	0.091	0.087	0.112	0.106	0.097	0.090	0.082	0.106				
***P. ochraceella* HT M BOLD:ADR5905METAT050-18**	0.090	0.097	0.100	0.088	0.090	0.082	0.079	0.087	0.094			
***P. corticivora* M BOLD:ADR4228METAT018-18 010922990**	0.108	0.117	0.105	0.112	0.091	0.096	0.093	0.091	0.120	0.100		
***P. fuscilinea*BOLD:AHC2564BSNHM1690-25 BGE 00019 F11**	0.114	0.114	0.108	0.107	0.098	0.102	0.096	0.095	0.110	0.107	0.091	

### Morphological analysis and exemplar sampling

The following criteria were used in selecting taxa for a morphological character matrix: (i) all taxa previously described in *Ptochoryctis* (with the exception of *P. perigramma*), (ii) all new taxa proposed to be described in *Ptochoryctis*, (iii) the type species of *Xylorycta*, *Xylorycta luteotactella* (Walker, 1864); (iv) representatives of *Athrypsiastis*, *Linoclostis*, *Metathrinca* and *Topiris*; (v) representatives of the type genus of each old world sub-family of Autostichidae*sensu*Wang and Li 2020; (vi) representatives of the type genera of Lecithocerinae and Ceuthomadarinae; and (vii) *Martyringa xeraula* as an outgroup (although it is included in Lecithoceridae as its basalmost taxon in Wang and Li 2020).

A single male (where known) of each taxon was examined. Females were not sampled (except in the case of *P. galbanea* where the male is unknown) as these taxa are not externally sexually dimorphic and their female genitalia, particularly in the xyloryctids and the genus *Ptochoryctis*, are poorly known and (with the exception of *Topiris*) the characters are not distinctive at the generic level.

Morphological data was generated for 47 taxa. These included 33 taxa for which molecular data was also obtained and an additional 14 taxa for which the only known specimens originate from Meyrick’s own collection (NHMUK accession B.M. 1938-290). No molecular data was sought from these specimens.

Where practicable, the examined specimen was a primary type. This was the case for (i) all species of *Ptochoryctis* except for *Ptochoryctis inviolata* (Meyrick, 1925), for which taxon the type has not yet been definitively identified, *Ptochoryctis alma* Meyrick, 1908 for which the male specimen was used; and *P. perigramma*, for which the type is lost, (ii) all xyloryctid exemplars except for *M. tsugensis*; and (iii) for *Automola pelodes* Meyrick, 1883 (now *Autosticha pelodes*), *Periacma ferialis* Meyrick, 1894, and *M. xeraula*.

Also, where practicable, the molecular and morphological data used for each taxon was obtained from the same specimen; in most cases, the specimen which is here designated as the holotype was used. Where different specimens of a taxon were used for molecular and morphological data, we have annotated the label for the relevant taxon in the morphological matrix with ch (= chimaeric) (indicated in Suppl. material 1).

The morphological matrix included 40 characters for 47 taxa and was created in and transposed using MS Excel (Suppl. material 2) and formatted for MrBayes input as a nexus file, in which both missing and inapplicable data were treated as missing (question marks). These morphological data were run in MrBayes v. 3.2.7 with command line execution using a single partition under a gamma rate model (lset rates = gamma). All variables (state frequencies, shape, revmat, and pinvar) were unlinked, a variable rate prior was used, and the run was set at 10,000,000 generations in two independent runs of four chains to ensure convergence (until average standard deviation of split frequencies approximated 0.005, potential scale reduction factor = 1.000), using a burn-in fraction of 0.25. The resulting consensus tree was plotted in FigTree v. 1.4.3 (http://tree.bio.ed.ac.uk) visually rooted by *Martyringa xeraula* and with posterior values of at least 0.94 displayed.

### Combined analysis

A combined analysis was run as a nexus file in MrBayes v. 3.2.7 for the 33 taxa for which we had DNA (COI-5P + nuclear gene data) as well as morphology (thus a total of 6121 characters including 40 morphological characters). This time we included the partial sequence of *P. minimella*, relying on the additional morphological data to place it. We used two partitions (morphology under gamma rate model as above, molecular data with an invgamma rate model (nst = 6); state frequencies, shape, revmat and pinvar variables all unlinked, run as before for 10 million generations in four chains with a burn-in of 0.25. The resulting consensus tree file was again plotted in FigTree v. 1.4.3 to show posterior support values with at least 0.94 posterior credibility. In FigTree the branches were then transformed to a cladogram and the tree exported in Newick format. The tree was then simplified in a text editor in order to remove branch lengths and taxon names were edited down to the taxon numbers that had been tagged to the end of each taxon label (e.g. in this case 0-33). The parenthetical tree was then duplicated to allow import into WinClada v. 1.00.08 (Nixon 1999–2002), after first inputting the 33-terminal morphological matrix and the tree was exported from WinClada for plotting as an emf file.

The combined morphological and molecular matrix was analysed in exactly the same way for all taxa used in the morphological analysis and the molecular analysis (again for a total of 6121 characters; 40 characters for 47 taxa formatted in TNT format) and in this case with terminals numbered 0-46.

All morphological characters and their states were checked using the character diagnoser in WinClada v. 1.00.08 and forward nonhomoplasious changes and forward changes with homoplasy or reversals are plotted in Fig. 4.

## Results

### Molecular data

Successful COI-5P sequences were obtained from a total of 28 exemplars within our *Ptochoryctis* materials (of which 15 were used in our analysis: Suppl. material 1). Among those 28 sequences, 24 sequences were obtained by Sanger sequencing with “failure tracking” at BOLD from specimens collected in or after 1982, 17 full DNA barcodes of 658 bp being obtained, while the remaining seven sequences represented partial fragments of 301 to 595 bp (BOLD Project METAT). Two full DNA barcodes were obtained from next generation sequencing from specimens collected in 1994 and another two full DNA barcodes through genome skimming from specimens collected in 1986.

For the purposes of Fig. 1 the following levels of support are considered significant: SHaLRT 80%; ABayes 0.95; UFBoot 95% (Minh et al. 2013; https://iqtree.github.io/doc/Frequently-Asked-Questions).

The output tree based on molecular data for 32 taxa (Fig. 1), recovers a *Ptochoryctis* clade comprising 14 taxa, of which four species, *P. eremopa* (type species of *Ptochoryctis*), *P. chalazopa*, *P. sundarbanica* and *P. corticivora* were previously described (defined here as the molecular *Ptochoryctis* clade) (SHaLRT = 93.4%; pp = 1; UFBoot = [92%]). Within this clade (*P. persicotincta* + *P. eremopa*), (*P. marmorella* + *P. chalazopa*) and (*P. corticivora* + *P. fuscilinea*) each form supported subclades, and there is limited support for subclades of (*P. flavalbella* + *P. persicotincta* + *P. eremopa*) and (*P. kitchingi + P. draconella* + *P. caputanatis*). The support for the molecular *Ptochoryctis* clade is submarginal in UFBoot only.

Fig. 1 also recovers the following clades as supported:

1. *Topiris candidella* Walker, 1863 + *T. lacteella* Sterling & Lees, 2025 + *T. salva* (Meyrick, 1932) + *T. albidella* Sterling & Lees, 2025 (defined here as the *Topiris* clade);
2. *Metathrinca argentea* Wang & Li, 2000 + *M. tsugensis* (defined here as the *Metathrinca* clade);
3. (*Oegoconia deauratella* + *O. quadripuncta*) + (*Holcopogon bubulcellus* + *Symmoca signatella* Herrich-Schäffer, 1854) + (*Autosticha modicella* + *A. pelodes*) (defined here as the molecular Autostichidae clade) (supported only by SHaLRT and ABayes);
4. *M. xeraula* + (*Lecithocera nigrana* + *Ceuthomadarus tenebrionellus*) (defined here as the molecular Lecithoceridae clade);
5. *X. luteotactella* + the *Topiris* clade + *Athrypsiastis penumbrella* Sterling & Lees, 2025 + *Athrypsiastis cheesmanae* Sterling & Lees, 2025 + the *Metathrinca* clade + the molecular *Ptochoryctis* clade (defined here as the molecular Xyloryctidae clade) (SHaLRT = 93.3%; ABayes pp = 0.996; UFBoot = 99%); and
6. the molecular Autostichidae clade + the molecular Xyloryctidae clade.

Within the molecular Autostichidae clade, (*O. quadripuncta* + *O. deauratella*) are recovered as a supported subclade and there is limited support for (*Symmoca signatella* + *Holcopogon bubulcellus*) and (*A. modicella* + *A. pelodes*).

### Molecular characters

Some potentially informative molecular characters are apparent for the DNA barcodes of the genus *Ptochoryctis* when the nucleotides are translated and the sequences are ordered against the nucleotide derived tree (Fig. 1).

*Ptochoryctis corticivora* + *P. fuscilinea* share a Lysine (K)/(primitively Arginine (R)) in complete amino acid position 156, representing a first and second position nucleotide CG->AA, and a Leucine (L)/primitively Phenylalanine (F), representing a third nucleotide position T->A in codon position 168. The first pair of nucleotide changes is unique in our dataset, the second recurs in *P. robinsoni*, *P. corticivora* and *P. fuscilinea*; the last two species are 9.1% pairwise divergent (Table 1). Also, for amino acid position 156, *P. corticivora* and *P. fuscilinea* show the nucleotide triplet AAA rather than CGA found in other *Ptochoryctis* and a few other taxa.

*Ptochoryctis corticivora*, *P. chalazopa*, and *P. marmorella* share a Threonine (T) rather than Alanine (A) in complete amino acid position 130, underlain by a first position G->A. This appears to be an example of conflict in our dataset, although only in *P. chalazopa* + *P. marmorella* is the underlying triplet ACT, which is consistent with them being sisters (at 2.7% pairwise divergence). In the light of the tree this would represent a codon convergence in *P. corticivora*.

*Ptochoryctis eremopa* + *P. persicotincta* exhibit another unique change, namely I (Isoleucine) to M (Methionine) in complete codon 152, representing a third position nucleotide T->A. This pair also share a Threonine (T) and not Alanine (A) in the 176^th^ complete codon, representing a first position G->A. They also share a Leucine (L) to Methionine (M) in complete position 19, underlain by a first position C->T and a third position T->G. Both these codon changes are unique for our dataset. Given that this pair are 2.9% pairwise divergent for the DNA barcode, these three synapomorphies are unlikely to be due to convergence.

Not only *P. eremopa* + *P. persicotincta*, but *P. flavalbella*, *P. minimella* [not used in the molecular only trees due to partial nucleotides] and *P. robinsoni*, share a Valine (V) rather than Alanine (A) in the 106^th^ complete codon, representing a second position C-T, states convergently shown by only *Topiris lacteella*. According to the molecular trees, this character only provides supporting data for the first pair, as *P. flavalbella* is submarginally supported as sister to *P. eremopa* + *P. persicotincta* (Fig. 1). Also, *P. flavalbella* has an underlying GTT in that position rather than GTA in the others.

Lastly, all *Ptochoryctis* (but unobserved in *P. minimella*: no data over relevant position) share an Alanine (A) -> Serine (S) in complete codon position 10. This is a local synapomorphy with an underlying 1st position G->T that is rather rare in Gelechioidea. The amino acid state S is only otherwise seen in our dataset in *Holcopogon bubulcellus*, but in that case the S is underlain by the triplet TCT and not TCA via the redundancy of the genetic code.

### Morphological data

The output tree based on morphological data from 47 taxa (Fig. 2), recovers the molecular Autostichidae clade with the inclusion also of *Periacma ferialis* and two lecithocerids, *C. tenebrionellus* and *L. nigrana*, as a submarginally supported grouping (pp = 0.94). Within that grouping, (*C. tenebrionellus* + *L. nigrana*) is recovered as a supported sub-clade (pp = 1), with a very long branch. There is thus no support morphologically for a separate Autostichidae clade.

All Xyloryctidae, i.e. *X. luteotactella*, *Athrypsiastis* (*A. phaeoleuca* Meyrick, 1910 + *A. penumbrella* + *A. symmetra* Meyrick, 1915 + *A. cheesmanae*), the *Topiris* clade, three taxa misplaced in *Ptochoryctis* (“*P.*” *alma* + “*P.*” *inviolata* + “*P.*“ *ochrograpta*), (*Linoclostis gonatias* + *L. musicodes* Meyrick, 1910), the *Metathrinca* clade and the *Ptochoryctis* clade together form a clade (defined here as the morphological Xyloryctidae clade) (pp = 1).

Fig. 2 recovers a *Ptochoryctis* clade comprising 21 taxa (defined here as the *Ptochoryctis* clade) (pp = 0.97). It also recovers the *Topiris* clade (pp = 0.96) as supported and the *Metathrinca* clade (pp = 0.92) as submarginally supported. Within the *Ptochoryctis* clade, there were no sub-clades, although (*P. robinsoni* + *P. kitchingi* + *P. anguillaris* + *P. draconella* + *P. caputanatis*), and (*P. sundarbanica* + (*P. chalazopa* + *P. marmorella*)) are recovered as unsupported groupings.

### Combined data

The output tree from the concatenation of the morphological data and the molecular data for those 33 taxa, including *P. minimella*, for which both datasets are available is shown in Fig. 3. This figure shows a supported *Ptochoryctis* clade of 15 taxa (pp = 1). The *Topiris* clade and the *Metathrinca* clade are also supported (both pp = 1). *A. penumbrella* + *A. cheesmanae* form a supported clade (defined here as the common data set *Athrypsiastis* clade) (pp = 0.97).

Within this *Ptochoryctis* clade, *P. persicotincta* + *P. eremopa* (pp = 1), *P. corticivora* + *P. fuscilinea* (pp = 1), *P. marmorella* + *P. chalazopa* (pp = 1), and *P. blanchella* + *P. corticivora* + *P. fuscilinea* (pp = 0.97) are recovered as sub-clades.

*Xylorycta luteotactella* + the common data set *Athrypsiastis* clade + the *Metathrinca* clade + the *Topiris* clade + this *Ptochoryctis* clade are recovered as a supported clade (defined here as the common data set Xyloryctidae clade) (pp = 1).

The molecular Autostichidae clade and the Lecithoceridae clade are both supported (pp = 1). The common data set Xyloryctidae clade + the molecular Autostichidae clade are recovered as a clade (pp = 1).

Within the molecular Autostichidae clade, *A. pelodes* + *A. modicella* (pp = 1) and *O. quadripuncta* + *O. deauratella* (pp = 1) and within the Lecithoceridae clade *C. tenebrionellus* + *L. nigrana* (pp = 1) are all recovered as sub-clades.

### Extended combined data

Fig. 4 is the output from the concatenation of the morphological data for the 47 taxa used for the purposes of Fig. 2 with the molecular data for the 33 taxa used for the purposes of Fig. 3.

In Fig. 4 the *Ptochoryctis* clade (pp = 0.99), the *Topiris* clade (pp = 1) and *Athrypsiastis penumbrella* + *A. cheesmanae* + *A. symmetra* + *A. phaeoleuca* (described here as the *Athrypsiastis* clade) (pp = 0.96) are recovered as supported clades. A grouping of (*M. argentea* + *M. tsugensis*) is unsupported. (*L. musicodes* + *L. gonatias*) is similarly unsupported.

The Xyloryctidae clade is a supported clade (pp = 1). The molecular Autostichidae clade + *P. ferialis* (the Autostichidae clade) is a supported clade (pp = 1). The Lecithoceridae clade is also supported (pp = 1).

Within the *Ptochoryctis* clade, (*P. draconella* + *P. caputanatis* + *P. minimella* + *P. kitchingi* + *P. anguillaris* + *P. robinsoni*) is an unsupported grouping*. P. chalazopa* + *P. marmorella* is a supported sub-clade and there is slightly submarginal support (pp = 0.94) for *P. persicotincta* + *P. eremopa*.

### Morphological characters

Informative morphological characters for the genus *Ptochoryctis* are shown in Fig. 4. Filled circles show forward nonhomoplasious change, whereas unfilled circles show forward changes with homoplasy or reversal. These characters are: R_3_ absent, M_3_ and CuA_1_ separate or connate in forewing (Fig. 4: character 16 state 2, Figs 43, 84), ventral surface of valva with short sparse setae (Fig. 4: character 31 state 1, Fig. 101); and membrane of sacculus with postero-distal thickening appressed to valva (Fig. 4: character 34 state 1, Figs 101, 104–110).

The Xyloryctidae clade is recovered with a number of informative characters. These are: larva on living plant matter (Fig. 4: character 0 state 2), adult resting posture highly tectiform (Fig. 4: character 1 state 1, Figs 32–34), forewing length < 3× forewing breadth (Fig. 4: character 2 state 1), antenna of male with dense white sensillae (Fig. 4: character 7 state 1, Fig. 83), forewing with iridescent scaling (Fig. 4: character 20 state 1), ventral surface of valva with setose membrane from costa (Fig. 4: character 31 state 3, Fig. 103), saccular process only from postero-distal margin of sacculus (Fig. 4: character 35 state 1, Fig. 112; and saccular process with setae and/or bristles (Fig. 4: character 36 state 1, Fig. 112).

The informative characters recovered for the Autostichidae clade are: uncus elongate and narrow (Fig. 4: character 24 state 2, Fig. 88) and lateral extensions of tegumen short compared to tegumen band (Fig. 4: character 28 state 2, Fig. 96).

The informative characters recovered for the Lecithoceridae clade were: antenna same length as forewing (Fig. 4: character 5 state 1), gnathos with hooked downward mesial projection (Fig. 4: character 25 state 5, Fig. 94), tegumen bridge to valva present (Fig. 4: character 29 state 1, Fig. 98) anellus lobes absent (Fig. 4: character 30 state 2); and ventral surface of valva with short dense setae (Fig. 4: character 31 state 0, Fig. 98).

### Pairwise distances

Pairwise distances are shown in Table 1. With the exception of *P. eremopa* and *P. persicotincta* (pairwise distance = 2.9%) and *P. chalazopa* and *P. marmorella* (pairwise distance = 2.7%), the pairwise distances between the 12 species of *Ptochoryctis* for which full DNA barcodes are available range from 8.4–11.7% (Table 1). In general, parts of the topology with greater pairwise distances are not supported.

Support for the two species pairs above is shown in Figs 1, 3. Support for (*P. chalazopa* + *P. marmorella*) is also shown in Fig. 4 whereas support for (*P. persicotincta* + *P. eremopa*) in Fig. 4 is submarginal. Support is also shown in Figs 1, 3 for (*P. fuscilinea* + *P. corticivora*), although in this case the pairwise distance is 9.1%. This pair is not supported in Fig. 4.

### Checklist of treated species

#### *Ptochoryctis* Meyrick, 1894

*Deloryctis* Meyrick, 1934, syn. nov.

*eremopa* group

*Ptochoryctis eremopa* Meyrick, 1894 (Type species)

*Ptochoryctis persicotincta* sp. nov.

*anguillaris* group

*Ptochoryctis anguillaris* Meyrick, 1914

*Ptochoryctis caputanatis* sp. nov.

*Ptochoryctis draconella* sp. nov.

*Ptochoryctis kitchingi* sp. nov.

*Ptochoryctis minimella* sp. nov.

*Ptochoryctis robinsoni* sp. nov.

*Ptochoryctis sundarbanica* Sterling & Singh, 2025

*chalazopa* group

*Ptochoryctis chalazopa* Meyrick, 1920

*Ptochoryctis marmorella* sp. nov.

*corticivora* group

*Ptochoryctis corticivora* (Meyrick, 1934; *Deloryctis*) comb. nov.

*Ptochoryctis blanchella* sp. nov.

*Ptochoryctis fuscilinea* sp. nov.

*Ptochoryctis* sp. BOLD:ADJ0350

Ungrouped

*Ptochoryctis acrosticta* Meyrick, 1906

*Ptochoryctis flavalbella* sp. nov.

*Ptochoryctis galbanea* (Meyrick, 1914)

*Amorbaea galbanea* Meyrick, 1914

*Ptochoryctis ochraceella* sp. nov.

*Ptochoryctis scionota* Meyrick, 1906

*Ptochoryctis simbleuta* Meyrick, 1907

*Ptochoryctis splendidella* sp. nov.

#### Genus *Metathrinca* Meyrick, 1908

*Metathrinca alma* (Meyrick, 1908), comb. nov.

*Ptochoryctis alma* Meyrick, 1908

*Metathrinca inviolata* (Meyrick, 1925), comb. nov.

*Ptochoryctis inviolata* Meyrick, 1925

*Metathrinca ochrograpta* (Meyrick, 1923), comb. nov.

*Ptochoryctis ochrograpta* Meyrick, 1923

*Metathrinca perigramma* (Meyrick, 1926), comb. nov.

*Ptochoryctis perigramma* Meyrick, 1926

### Morphological systematics

#### Key to differentiate genera

**Table:** 

1	Sacculus with thickened postero-distal membrane appressed to valva	***Ptochoryctis*** (Figs 101, 104–110)
–	Sacculus with articulated postero-distal saccular process	**2**
2	R_3_ present in forewing	**3**
–	R_3_ absent in forewing	**4**
3	Medial plate of gnathos lightly sclerotised and weakly projecting posteriorly from lateral arms, or absent	*** Topiris ***
–	Medial plate of gnathos strongly sclerotised and strongly projecting posteriorly from the lateral arms	*** Athrypsiastis ***
4	M_1_ absent in forewing	*** Linoclostis ***
–	M_1_ present in forewing	*** Metathrinca ***

#### Key to groups of *Ptochoryctis*

The male of *P. galbanea* (an ungrouped species) is unknown

**Table:** 

1	Labial palps short (< 2× diameter of eye)	**2**
–	Labial palps long (2.5× or more than diameter of eye)	**3**
2	Dark markings covering most of forewing	***chalazopa* group**
–	Forewing iridescent white except for dark irregular dorso-medial blotch and dark postmedial streak	***anguillaris* group**
3	Antenna of male serrate	***corticivora* group**
–	Antenna of male (if known) bipectinate	**4**
4	Posterior margin of gnathos with deep V-shaped emargination	***eremopa* group**
–	Posterior margin of gnathos without deep V-shaped emargination	**ungrouped**

#### Key to species of *Ptochoryctis* based on external features

**Table:** 

1	Hindwings uniformly dark fuscous	***P. galbanea*** (Fig. 24)
–	Hindwings not uniformly dark fuscous	**2**
2	Labial palps long (length 2.5× or more than diameter of eye)	**3**
–	Labial palps short (length 2× or less than diameter of eye)	**13**
3	Antenna of male serrate	**4**
–	Antenna of male bipectinate	**6**
4	Distal half of forewing principally chocolate brown	***P. corticivora*** (Fig. 19)
–	Distal half of forewing not principally chocolate brown	**5**
5	Forewing with large, dark sub-basal streak	***P. fuscilinea*** (Fig. 21)
–	Forewings without large, dark sub-basal streak	***P. blanchella*** (Fig. 20)
6	Basal flagellomeres of antenna without white scaling	**7**
–	Basal flagellomeres of antenna with white scaling	**8**
7	Forewing colour brown	***P. ochraceella*** (Fig. 25)
–	Forewing colour pale yellow	***P. flavalbella*** (Fig. 23)
8	Forewing with contrasting brown markings towards dorsum	**9**
–	Forewing without contrasting brown markings towards dorsum	**10**
9	Forewing with two well-defined brown markings towards dorsum	***P. splendidella*** (Fig. 28)
–	Forewing with diffuse brown marking towards dorsum	***P. simbleuta*** (Fig. 27)
10	Forewing ground colour white	**11**
–	Forewing ground colour off-white	**12**
11	Forewing with small dark sub-apical spot	***P. acrosticta*** (Fig. 22)
–	Forewing without small dark sub-apical spot	***P. scionota*** (Fig. 26)
12	Hindwing white	***P. persicotincta*** (Fig. 7)
–	Hindwing dull pale brown	***P. eremopa*** (Fig. 5)
13	Forewing with large, blackish rectangular blotch covering most of distal third	**14**
–	Forewing without large, blackish rectangular blotch covering most of distal third	**15**
14	Costa of forewing strongly angulated at base	***P. marmorella*** (Fig. 17)
–	Costa of forewing smoothly curved at base	***P. chalazopa*** (Fig. 15)
15	Forewing with brown postmedial blotch not interrupted with white	**16**
–	Forewing with brown postmedial blotch interrupted with white	**17**
16	Forewing with small apical streak from costa	***P. caputanatis*** (Fig. 36)
–	Forewing without small apical streak from costa	***P. anguillaris*** (Fig. 35)
17	Antenna serrate	**18**
–	Antenna bipectinate	**19**
18	Postmedial blotch strongly interrupted white	***P. minimella*** (Fig. 39)
–	Postmedial blotch slightly interrupted white	***P. draconella*** (Fig. 37)
19	Flagellomeres of antenna black and white banded	***P. sundarbanica*** (Fig. 41)
–	Flagellomeres of antenna not black and white banded	**20**
20	Forewing strongly marked brown medio-dorsally	***P. robinsoni*** (Fig. 40)
–	Forewing not strongly marked brown medio-dorsally	***P. kitchingi*** (Fig. 38)

#### Key to species of *Ptochoryctis* based on male genitalia

**Table:** 

1	Gnathos with single medial posterior projection	**2**
–	Gnathos otherwise	**7**
2	Medial projection of gnathos long, straight, pointed downwards	***P. kitchingi*** (Fig. 51A)
–	Medial projection of gnathos otherwise	**3**
3	Distal margin of valva straight	***P. robinsoni*** (Fig. 53A)
–	Distal margin of valva curved	**4**
4	Costal margin of valva strongly concave	***P. draconella*** (Fig. 50A)
–	Costal margin of valva not strongly concave	**5**
5	Length of valva approximately the same as width	***P. anguillaris*** (Fig. 48A)
–	Valva almost 2× as long as wide	**6**
6	Valva pointed at apex	***P. flavalbella*** (Fig. 61A)
–	Valva rounded at apex	***P. caputanatis*** (Fig. 49A)
7	Gnathos with two broad lateral posterior projections	**8**
–	Gnathos otherwise	**13**
8	Uncus posteriorly bifid	**9**
–	Uncus not posteriorly bifid	**10**
9	Posterior apex of uncus deeply emarginate	***P. minimella*** (Fig. 52A)
–	Posterior apex of uncus slightly emarginate	***P. splendidella*** (Fig. 65A)
10	Postero-distal membrane of sacculus short and broad	***P. ochraceella*** (Fig. 62A)
–	Postero-distal membrane of sacculus long and narrow	**11**
11	Apical process of valva present	***P. fuscilinea*** (Fig. 59A)
–	Apical process of valva absent	**12**
12	Uncus narrow except towards base	***P. blanchella*** (Fig. 58A)
–	Uncus broad except towards posterior apex	***P. simbleuta*** (Fig. 64A)
13	Gnathos with two narrow lateral posterior projections	**14**
–	Gnathos with medial band without projection(s)	**15**
14	Costal margin of valva concave	***P. eremopa*** (Fig. 44A)
–	Costal margin of valva straight	***P. persicotincta*** (Fig. 47A)
15	Apex of valva with long process	**16**
–	Apex of valva without long process	**17**
16	Posterior apex of uncus tapering	***P. sundarbanica*** (Fig. 54A)
–	Posterior apex of apex not tapering	***P. acrosticta*** (Fig. 60A)
17	Distal margin of valva straight	***P. scionota*** (Fig. 63A)
–	Distal margin of valva not straight	**18**
18	Valva strongly tapering	***P. corticivora*** (Fig. 57A)
–	Valva not strongly tapering	**19**
19	Valva broad towards apex	***P. chalazopa*** (Fig. 55A)
–	Valva narrowing towards apex	***P. marmorella*** (Fig. 56A)

##### Genus Ptochoryctis

Taxon classificationAnimaliaLepidopteraAutostichidae

Meyrick, 1894

796B4675-6344-5BDA-9254-20A03753A0B0

Ptochoryctis Meyrick, 1894: 19. Type species Ptochoryctis eremopa Meyrick by original designation.
Deloryctis Meyrick, 1934: 464. Type species Deloryctis corticivora Meyrick by original designation. syn. nov.

###### Diagnosis.

Very small to medium sized xylorycytid species. Adults have a strongly tectiform resting posture (Figs 32–34) and the antenna in the male is bipectinate or serrate (Figs 82, 83). The combination in the forewing of R_3_ absent and M_3_ and CuA_1_ separate (or in a single case connate) is diagnostic (Figs 43, 84). In the male genitalia, the thickened membranous postero-distal extension of the sacculus, which is unarticulated, appressed to the valva and without setae or bristles (Figs 101, 104–110), is diagnostic, as is the valva with short sparse setae (Fig. 101). Also, the absence of an articulated saccular process distinguishes the *Ptochoryctis* from other Xyloryctidae. In addition, in many species, there is a small apical process on the valva. The female genitalia, insofar as they are known, are diagnostic at the species level but there are currently no characters which are diagnostic at the generic level.

###### Description.

**Adult**. ***Head***: External ocelli absent. Frons with appressed scales, vertex with tufts of long scales projecting laterally from sides of occiput, a ruff of long thin scales on posterior margin of occiput projecting posteriorly, overlaying a collar of broad lamellate scales on anterior margin of prothorax, pointing posteriorly. Pilifers small with short bristle tufts. Maxillary palps small. Labial palps short (< 2× diameter of eye) in *anguillaris* and *chalazopa* groups, otherwise long (exceeding 2.5× diameter of eye), strongly recurved, second segment longer than third. Haustellum with basal portion scaled, usually silver white. Antenna ¾ length of forewing, scape without pecten, pedicel short and broad. Flagellum of male antenna bipectinate or serrate for over ½ to ¾ of length, pectinations/serrations covered in short sensillae, apical portion filiform, dark but with but with white or coloured scaling usually present on at least basal portion and sometimes for most of length. Flagellum of female antenna filiform throughout. ***Thorax***: Thorax with appressed lamellate scales, tegulae short. Tibial spurs 0–2–4. Mid and hind legs generally white, hind legs with a thin tuft of long scales. Frenulum of male a single bristle from base of hindwing coupling with retinaculum under a scaled flap of variable length towards the base of Sc on the forewing. Forewing venation: R_1_ from just prior to ½ to 2/3 discal cell, R_3_ absent, R_4_ and R_5_ stalked, R_4_ pre-apical (in one case apical), R_5_ post-apical (in three cases apical), M_1_, M_2_ and M_3_ present, M_3_ and CuA_1_ separate (or in one instance connate), CuP present. Hindwing venation: Rs and M_1_ originating from a common stem, M_3_ and CuA_1_ stalked. Forewing broad, sometimes slightly elongate, costa of forewing straight or gently arched, apex rounded to somewhat pointed, termen often angled substantially inwards, tornus obtuse, cilia long, often very long towards tornus, sometimes producing a dorsal crest (Fig. 34). Forewing generally without line of dark scaling at edge of base of costa, strongly patterned in *anguillaris* and *chalazopa* groups and in *P. corticivora*, unpatterned or weakly patterned otherwise.

***Male genitalia***. Uncus short and broad anteriorly, posterior apex variable, anterior margin of dorsal surface usually strongly emarginated. Gnathos with lateral arms, medial plate of gnathos with lateral or medial posterior projection(s) or sometimes a curved band. Tegumen band broad, arched, lateral extensions of tegumen approximately same length as width of tegumen band. Vinculum generally U-shaped, robust, not projecting substantially beyond base of valvae. Saccus small. Base plate of juxta variable, anellus lobes broad, generally extending as far as costal margin of valva. Valva short and broad, ventral surface generally with patch of sparse fine setae in distal half, weak sclerite ventrally, small apical process or nodule often present. Sacculus indistinct with thickened membranous postero-distal extension (visible in preparations towards centre of valva) which is unarticulated, appressed to the valva, and without setae or bristles (Figs 101, 104–110). Aedeagus small and simple, generally slightly curved, often with small posterior projection. Bulbus ejaculatorius slightly longer than aedeagus, not coiled, hood rounded.

***Female genitalia***. Papillae anales generally short and broad, apophyses posteriores slightly longer than apophyses anteriores, ostium generally small and circular, sclerotised lamella sometimes present, antrum generally short, lightly sclerotised, ductus bursae membranous, corpus bursae often with single signum.

###### Early stages and biology.

Larvae of the four species for which data is available feed on bark, making webs which incorporate pieces of refuse and bark. They have been found on *Hevea* (Euphorbiaceae), *Schleichera* (Sapindaceae), *Thea* (now *Camellia*) (Theaceae) and *Coffea* (Rubiaceae). There is a further record of a *Ptochoryctis* sp. on leaves of *Phyllanthus pectinatus* (Phyllanthaceae) (Robinson et al. 2001: 345). The larva of *P. simbleuta* is recorded as feeding on the bark of shoots, eating right through to the cambium and thus killing the branch or plant. *P. chalazopa* and *P. corticivora* have been found in association with rubber plantations, the latter recorded as feeding on the outer bark of rubber trees (Bradley, 1959). *P. caputanatis* and *P. sundarbanica* have been found in degraded mangrove and secondary forest habitats. Adults of a number of species have been found at light at night.

###### Distribution.

The genus *Ptochoryctis* is known from India, Bangladesh, Sri Lanka, Myanmar, China (Hong Kong), Thailand, Brunei, Indonesia (Java), Peninsular Malaysia and Malaysian Borneo (see Fig. 113).

###### Status and conservation.

The only verifiable records of described species of this genus post-2000 are of *P. caputanatis* and *P. sundarbanica*. Anon (1958) describes an infestation of larvae of the outer bark of rubber trees in Selangor, Malaysia identified by Bradley (1959) as *P. corticivora*. *Ptochoryctis* sp. BOLD:ADJ0350 has been identified by a neighbor-joining search using *P. fuscilinea* on BOLD and a small number of images of specimens posted on iNaturalist can be properly identified to this genus or, in one or two cases, to the *chalazopa* group. *P. caputanatis* has been found commonly in one restricted area of mangrove forest in the World Wildlife Fund Reserve at Mai Po Marshes in Hong Kong, China and has been sporadically recorded elsewhere in Hong Kong. The quality of the mangrove forest in the Mai Po Marshes, which is clearly a stronghold for this species, has deteriorated and management is needed to improve the quality of the mangrove at this reserve. Further work is required to determine the status and conservation needs of other species.

###### Note on Meyrick’s etymology.

*Ptochoryctis*Meyrick 1894 – *ptochos* (Gr.), strictly means a beggar but is sometimes also used to mean poor. In modern Greek it is the legal term for a bankrupt. *oryctes* (gr.) is one who digs. This is a reference to Meyrick’s recently created family Xyloryctidae, the diggers of wood. The somewhat plain genotype, *P. eremopa*, is certainly a poor relation to some of the strikingly marked and iconic Australian species which Meyrick placed in his newly established Xyloryctidae (Meyrick, 1890). It is evident from Meyrick’s endings that he intended its gender to be female.

#### The *eremopa* group

This is a supported sub-clade of two species, known from Myanmar and Thailand. The adults are pale and plain in appearance. In the male genitalia of both species there is a small apical process on the valva and the posterior margin of the gnathos is strongly emarginate.

##### Ptochoryctis eremopa

Taxon classificationAnimaliaLepidopteraAutostichidae

Meyrick, 1894

19965525-D480-57C6-87EE-FFDBBBE6F72F

Figs 5, 6, 42–46B, 106

Ptochoryctis eremopa Meyrick, 1894: 19.

###### DNA barcodes.

BIN, BOLD:AGD1581 (Process IDs METAT290-24, METAT291-24).

###### Type material.

Myanmar: ***Holotype*** • ♂, Koni, Burma, NM. 88, fwl 7.5 mm., specimen no. NHMUK010219731; slide no. JFGC 7683. Holotype by monotypy.

###### Additional material examined.

• 2♂, N. Thailand, Chiang Mai, Doi Suthep-Pui NP, 1490 m, 24.v.1989, IJ Kitching and AM Cotton leg., specimen no. NHMUK013700421, slide no. NHMUK014332874; specimen no. NHMUK013700422; • 1♂, NW Thailand, Chiang Mai, Doi Suthep-Pui NP, 1460 m, 26.iv-10.v.1989 AM Cotton leg., specimen no. NHMUK013700126, slide no. NHMUK014332875; • 1♂, NW Thailand, Chiang Mai, Doi Suthep-Pui NP, 1440 m, 29.iv-4.v.1988, GS Robinson leg., specimen no. NHMUK013700857.

###### Diagnosis.

The ochreous whitish forewings with scattered ochreous scaling, an obscure dorsal patch and, in fresh specimens, an obscure medial spot, distinguish this species from other *Ptochoryctis*. *P. flavalbella* and *P. persicotincta* are externally somewhat similar but are brighter in appearance and lack the scattered ochreous scaling and the obscure medial spot present in fresh specimens. The male genitalia of *P. eremopa* are somewhat similar to those of *P. fuscilinea* and *P. persicotincta*. However, in *P. fuscilinea* the lateral posterior projections of the medial plate of the gnathos are broadly rounded, the emargination of the posterior margin of the gnathos is shallower and the postero-distal thickened membranous extension of the sacculus is more elongate. In *P. persicotincta*, the posterior apex of the uncus is more rounded and the apical area of the valva is broader.

**Figures 5–22.**
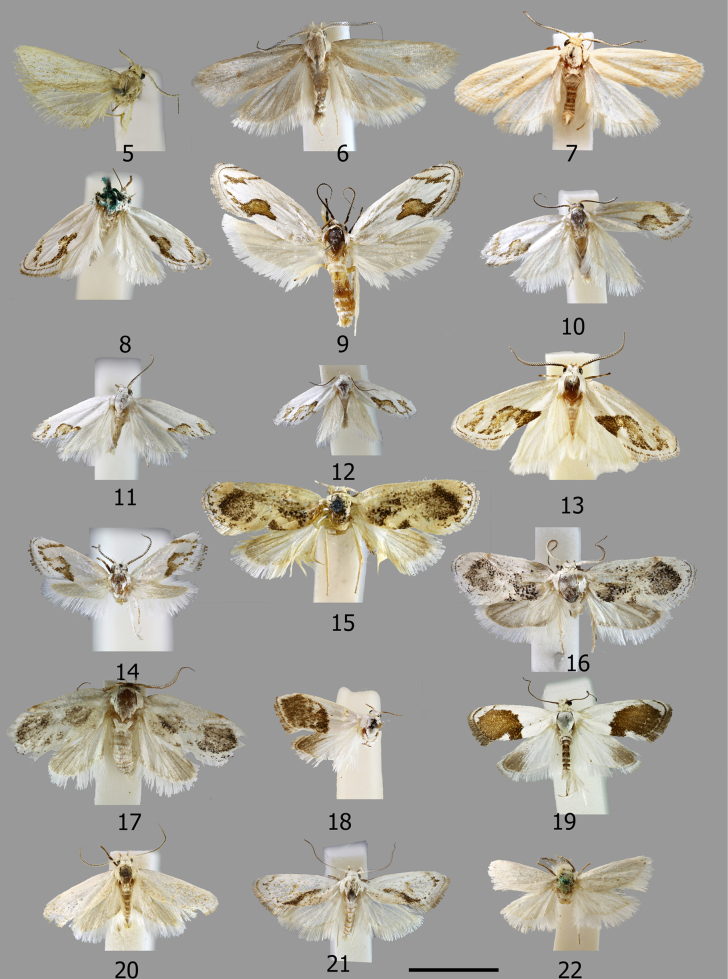
Dorsal images of *Ptochoryctis* spp. **5**. *Ptochoryctis eremopa* Meyrick, 1894, holotype • ♂; **6**. *P. eremopa* Meyrick, 1894 • ♂; **7**. *P. persicotincta* sp. nov., holotype • ♂; **8**. *P. anguillaris* Meyrick, 1914, paralectotype ♀; **9**. *P. caputanatis* sp. nov., holotype • ♂; **10**. *P. draconella* sp. nov., holotype • ♂; **11**. *P. kitchingi* sp. nov., holotype • ♂; **12**. *P. minimella* sp. nov. holotype • ♂; **13**. *P. robinsoni* sp. nov. holotype • ♂; **14**. *P. sundarbanica* Sterling & Singh, 2025 holotype • ♂; **15**. *P. chalazopa* Meyrick, 1920 holotype ♀; **16**. *P. chalazopa* Meyrick, 1920 • ♂; **17**. *P. marmorella* sp. nov. holotype • ♂; **18**. *P. corticivora* (Meyrick, 1934) holotype • ♂; **19**. *P. corticivora* (Meyrick, 1934) • ♂; **20**. *P. blanchella* sp. nov. paratype • ♂; **21**. *P. fuscilinea* sp. nov. holotype • ♂; **22**. *P. acrosticta* Meyrick, 1906 lectotype • ♂. Scale bar: 5 mm.

**Male** (Figs 5, 6). Forewing length 7.0–8.0 mm. ***Head***: frons with pale ochreous white appressed scales, vertex with tufts of long cream white scales from sides of occiput pointing inwards, pale ochreous scales on posterior margin of occiput pointing posteriorly, partly overlaying collar of flat almost white scales pointing posteriorly from anterior margin of prothorax. Pilifers with small tufts of bristles. Maxillary palps ochreous. Labial palps long (at least 2.5× diameter of eye), strongly recurved, second segment pale ochreous, strongly curved, approximately same length as third segment, third segment thin and straight with whitish appressed scales. Haustellum with silver scaling on basal portion. Antenna ~ ¾ length of forewing, bipectinate, scape whitish ochreous, flagellum with dark pectinations covered in short white sensillae for ~ ¾ of length, reducing at ¾, apical portion of flagellum filiform, flagellum whitish ochreous apart from dark apical portion. ***Thorax***: whitish ochreous, tegulae whitish ochreous; foreleg with femur pale ochreous, tibia and tarsus brown, narrow tibial epiphysis, mid and hindlegs whitish ochreous, long thin whitish scale tufts on femur of mid and hind legs. Forewing broad, costa slightly arched towards termen, otherwise straight, apex broadly pointed, termen angled substantially inwards, tornus obtusely rounded, forewing off white, iridescent, unmarked except for a line of dark brown scales at edge of base of costa to ~ 1/5 and scattered scales tipped darker ochreous, a thicker patch of darker tipped scales on dorsum, and, in fresh specimens, darker tipped scales forming an obscure medial spot, cilia with a faint ochreous line at ~ 1/3 and a very faint ochreous terminal band. Hindwing as broad as forewing, apex broadly pointed and slightly projecting, white, unmarked apart from a small patch of ochreous scaling at apex. Ventrally: forewing pale ochreous with darker scaling between costa and Sc of forewing and along veins of forewing; hindwing silver.

**Female**. Unknown.

***Pre-genital abdomen*** (Fig. 42). Pale ochreous with a small pale ochreous anal tuft. Tergal spines on posterior parts of T2–T7, T8 and sternites weakly sclerotised. Apodemes straight, venulae sinuate.

***Male genitalia*** (Figs 44–46, 106). Uncus short and broad anteriorly, tapering to a narrow point at posterior apex, anterior margin of dorsal surface strongly emarginated. Gnathos with short lateral arms, medial plate strongly emarginated with two long sclerotised digitate projections laterally (Fig. 45). Tegumen with broad band dorsally, lateral extensions of tegumen same length as width of tegumen band. Vinculum broad, robust, U-shaped, base projecting beyond base of valvae. Saccus broad, projecting slightly beyond base of valvae. Juxta with hexagonally shaped basal plate, anellus lobes broad, not extending as far as costal margin of valva. Valva broad medially, tapering towards apex, apex rounded, small sclerotised apical process, costal margin concave, ventral margin slightly sinuate, ventral surface with short sparse setae, triangular ventral sclerite medially. Sacculus broad, indistinct, postero-distal membranous extension narrow, terminating near apex of ventral sclerite. Aedeagus almost straight, tapering posteriorly, with distal projection.

###### Biology and early stages.

Unknown.

###### Distribution.

Myanmar, northern Thailand.

###### Note on Meyrick’s etymology.

*eremopa* Meyrick, 1894 – from *eremos* (Gr.), empty, barren, or desert; and *ops* (gr.), face. The unmarked, off white, wings sprinkled with ochreous scales give an impression of a sandy surface and a desert is likely to have been what Meyrick had in mind.

##### Ptochoryctis persicotincta sp. nov.

Taxon classificationAnimaliaLepidopteraAutostichidae

6A2FA0A4-6452-5237-9326-2E3A975990B2

https://zoobank.org/DEE6FEDB-75D0-47C2-808E-44B5B8882E21

Figs 7, 47 A, B

###### DNA barcodes.

BIN, BOLD:AGD1583 (Process IDs METAT288-24, 289-24).

###### Type material.

Thailand: ***Holotype*** • ♂, W. Thailand, Uthai Thani Dist., Khao Nang Rum, 400 m, 6–8.vi.1986, Col. M.G. Allen leg., fwl 7 mm, specimen no. NHMUK010219784, slide no. NHMUK010316869; ***Paratype*** • ♂, same collection details as holotype, specimen no. NHMUK010219781, slide no. NHMUK010316868.

###### Diagnosis.

Externally, the unmarked peach white forewings distinguish this species from others in the genus. The forewings of *P. flavalbella* are similar, but *P. flavalbella* is slightly larger and the forewing colour is darker and more yellowish. The male genitalia of *P. eremopa* are similar but in *P. persicotincta* the valva is broader towards the apex and the posterior apex of the uncus is more rounded (Figs 44A, 46A, 47A).

###### Description.

**Male** (Fig. 7). Forewing length 6.0–6.5 mm, wingspan 13.0–14.0 mm. ***Head***: Frons worn but with pale ochreous scaling laterally, vertex with two tufts of long pale ochreous scales laterally from occiput and collar of long pale ochreous scales from anterior margin of prothorax, projecting posteriorly. Maxillary palps and pilifers not visible. Labial palps long (3× diameter of eye), recurved, basal segment with small pale ochreous tuft of scales, second segment longer than third, strongly recurved, both segments with appressed pale ochreous scaling. Haustellum scaled at base. Antenna ¾ length of forewing, bipectinate, scape pale ochreous, blackish pectinations to ¾, apically filiform, pectinations covered in small white sensillae, thick pale ochreous scaling on dorsal surface of flagellomeres. ***Thorax***: thorax and tegulae peach white, foreleg, middle leg and hindleg pale ochreous, foreleg with small tibial epiphysis, hindleg with tuft of long whitish scales. Forewing moderately broad, costa almost straight, apex rounded, termen angled inwards, tornus very obtusely angled; peach white, scaling iridescent, completely unmarked, a patch of thicker scaling on dorsum, cilia peach white with faint darker lines at 1/3 and terminally. Hindwing silvery white, tinged peach white at apex, otherwise unmarked. Ventrally, forewing with ochreous scaling, darker towards costa, hindwing silvery white, unmarked.

**Female**. Unknown.

***Pre-genital abdomen***. Peach white, long peach white anal tuft. Robust tergal spines on posterior part of T2 to T7, strongly visible in dried specimens, T8 and sternites weakly sclerotised. Apodemes and venulae almost straight.

***Male genitalia*** (Fig. 47). Uncus short, broad at base, anterior margin of dorsal surface strongly emarginated, tapering to a rounded point at posterior apex. Gnathos with short lateral arms and broad plate with strongly emarginate posterior margin and two well sclerotised lateral digitate posterior projections. Tegumen with broad band dorsally, lateral extensions of tegumen slightly shorter than width of tegumen band. Vinculum robust, broad, U-shaped, base projecting slightly beyond base of valvae. Saccus very short. Juxta with circular basal plate, anellus lobes not extending as far as costal margin of valva. Valva broad throughout, costal and ventral margins almost straight, distal margin and apex rounded, a small sclerotised nodule at apex, indistinct ventral sclerite, short sparse setae on ventral surface of valva. Sacculus ovate, broad, postero-distal membranous extension short and broad throughout. Aedeagus slightly curved, tapering to a broad point, with distal projection.

###### Biology and early stages.

Early stages unknown. Adults found in June at 400 m.

###### Distribution.

Western Thailand.

###### Etymology.

*Ptochoryctis persicotincta* – from *persicum* (Lat.), a peach and *tinctus* (Lat.) dyed or tinted, a reference to the slight yellowish tinge to the forewings which are reminiscent of the colour of peach flesh. The epithet is an adjective in the nominative singular.

#### The *anguillaris* group

**Diagnosis**. Species in this group can be recognised by their small (< 2× diameter of eye), recurved labial palps which are very closely appressed to the eye, iridescent silver white ground colour and unusual dark postmedial markings on the forewing. There is no support for this group as a sub-clade. This grouping is for convenience only and is not intended to have any taxonomic significance, although the analysis in Fig. 4 shows an unsupported topology for the members of the group apart from *P. sundarbanica*.

##### Ptochoryctis anguillaris

Taxon classificationAnimaliaLepidopteraAutostichidae

Meyrick, 1914

AF172697-F32F-5C01-B3E9-C469D1667EE2

Figs 8, 35, 48 A, B, 68

Ptochoryctis anguillaris Meyrick, 1914: 778.

###### DNA barcode.

N/A.

###### Type material.

Sri Lanka: ***Lectotype*** • ♂ Maskeliya, Ceylon. Alston. 12.06, specimen no. NHMUK010219707, slide no. JFGC 7697, fwl 6.5 mm. Lectotype designated by Gates Clarke (Clarke (1955b: 493). ***Paralectotype*** • ♀ Hambantota, Ceylon, JBF, 18.10.08, specimen no. NHMUK010219706, slide no. NHMUK014333410.

###### Diagnosis.

Externally, forewing markings similar to *P. caputanatis*. However, the basad projection of the medio-dorsal blotch is more rounded in this species than in *P. caputanatis*, the postmedial streak in *P. anguillaris* lacks the strong lateral projections present in *P. caputanatis* and *P. anguillaris* lacks the dark apical streak present in *P. caputanatis* (Figs 35, 36). In the male genitalia, the valva of *P. anguillaris* is much shorter laterally than that of *P. caputanatis*. Also, the costa of the valva is slightly concave in *P. caputanatis* whereas it is straight in *P. anguillaris* (Figs 48A, 49A).

###### Description.

**Male**. Forewing length 5.0 mm, wingspan 12.0 mm. ***Head***: frons with a few remaining white scales, vertex worn or destroyed by verdigris. Pilifers and maxillary palps not visible. Labial palps very small (1.5× diameter of eye), recurved, white, second segment strongly curved with appressed white scales, third segment almost straight, thinly covered in appressed white scales. Haustellum with base covered in silver white scales. Antenna with only scape and part of one flagellum remaining, basal part without pectinations. ***Thorax***: with silver scales, tegulae missing; forelegs with femur whitish, tibia and tarsus brown, small tibial epiphysis, mid and hind legs white, hind legs with thin tuft of long white scales. Forewing moderately broad, elongate, costa gently arched, apex obtusely rounded, termen angled substantially inwards, tornus very obtusely rounded, iridescent silver white, basal half unmarked, medio-dorsal blotch dark brown above and below, paler brown centrally, a broad rounded projection basad along medial fold and a dark brown projection along the termen not interrupted with white, postmedial streak without strong lateral projections, cilia white with dark brown internal and terminal lines. Hindwings broader than forewings, shining silver white, unmarked. Ventrally: forewing with some brown scaling; hindwing silver.

**Female** (Figs 8, 35). Forewing length 6.5 mm, wingspan 15.0 mm. Otherwise similar to male.

***Pre-genital abdomen***. White. patches of tergal spines on posterior parts of T2–T7; T8 and sternites unsclerotised. Apodemes short, straight; venulae short, almost straight.

***Male genitalia*** (Fig. 48A, B). Uncus short and broad, substantially tapering towards posterior apex. Gnathos with broad lateral arms, medial plate with upturned, strongly sclerotised medial posterior projection. Tegumen band broad, lateral extensions of tegumen same length as width of tegumen band. Vinculum robust, U-shaped, base not projecting beyond base of valvae. Juxta with sclerotised U-shaped basal plate, anellus lobes curved and broad, almost reaching as far as costal margin of valva. Valva broad and very short, costal margin straight, ventral margin gently convex, apex rounded, a patch of fine sparse setae on distal half, small weak ventral sclerite. Sacculus indistinct and small, postero-distal extension membranous long, broad basally, tapering distally. Aedeagus simple, moderate length, slightly tapering distally.

***Female genitalia*** (Fig. 68). Papillae anales short and broad. Apophyses short, apophyses posteriores slightly longer than apophyses anteriores. Eighth tergite and eighth sternite rectangular. Ostium circular. Antrum short, somewhat funnelled, with small lateral sclerites, otherwise membranous. Ductus bursae short, membranous. Corpus bursae ovate, with small lateral, almost linear, signum, medially.

###### Biology and early stages.

Early stages unknown. Adults found in October and December.

###### Distribution.

Sri Lanka.

###### Note on Meyrick’s etymology.

In our view Meyrick’s original etymology is as follows: *anguillaris* Meyrick, 1914 – from *anguilla* (Lat.), eel. The complex postmedial marking on the forewing of the *anguillaris* group of *Ptochoryctis* species can be described in a number of ways. To Meyrick, those of *P. anguillaris* resembled an eel.

##### Ptochoryctis caputanatis sp. nov.

Taxon classificationAnimaliaLepidopteraAutostichidae

2AEB16DD-197E-54A5-B564-8CBD185056E6

https://zoobank.org/21A4E3AF-B337-4B65-9FB5-E110C9925588

Figs 9, 33, 36, 49 A, B, C, 69, 80, 84, 99, 101

###### DNA barcodes.

BIN, BOLD:ADR3481 (Process IDs METAT005-18, METAT006-18).

###### Type material.

Hong Kong, China: ***Holotype*** • ♂ Hong Kong, China, Mai Po NR, 22.4889°N, 114.0420°E, 2 m, 30 May 2016, M.J. Sterling leg., fwl 6 mm, specimen no. NHMUK010923223; slide no. NHMUK010316363, BIN, BOLD:ADR3481. ***Paratypes*** (10♂, 3♀) • 1♂ Hong Kong, China, Mai Po NR, 22.48°N, 114.04°E, 2 m, 12 May 2017, M.J. Sterling leg. specimen no. NHMUK010219710, slide no. NHMUK010316453; • 1♂, Hong Kong, China, Mai Po NR, 22.48°N, 114.04°E, 2 m, 12 May 2017, M.J. Sterling leg. specimen no. NHMUK010219764, slide no. NHMUK010316863; • 1♂, Hong Kong, China, Tai Yeung Che, Lam Tsuen, Tai Po, New Territories, 22.447°N, 114.128°E, 65 m, 7.6.2018, M.J. Sterling leg. specimen no. NHMUK010219765, slide no. NHMUK010316862; • 1♂, Hong Kong, China, Mai Po NR, 22.48°N, 114.04°E, 2 m, 12 May 2017, M.J. Sterling leg. specimen no. NHMUK013700855, slide nos. NHMUK010316861 (genitalia), NHMUK014333419 (wings); • 6♂, same collection data as holotype, specimen nos. NHMUK010219766; NHMUK010219767; NHMUK013701072; NHMUK013701073; NHMUK013701074; NHMUK010219768; • 1♀; Hong Kong, China, Tai Yeung Che, Lam Tsuen, Tai Po, New Territories, 22.447°N, 114.128°E, 65 m, 8.6.2018; M.J. Sterling leg. specimen no. NHMUK010219717; slide no. NHMUK010316454; • 1♀; Hong Kong, China, Mai Po NR, 22.48°N, 114.04°E, 2 m, 30 May 2016, M.J. Sterling leg. specimen no. NHMUK010923244, slide no. NHMUK010316455; • 1♀ Hong Kong, China, Mai Po NR, 22.48°N, 114.04°E, 2 m, 12 May 2017, M.J. Sterling leg. specimen no. NHMUK013696931, slide no. NHMUK014333413.

###### Diagnosis.

See Diagnosis for *P. anguillaris* above.

###### Description.

**Male** (Figs 9, 33, 36, 80, 84, 99, 101). Forewing length 5.5–7.0 mm, wingspan 12.5–15.0 mm. ***Head***: frons white, vertex with short tufts of white lamellate scales from sides of occiput and further tufts of long white scales on posterior margin of occiput projecting posteriorly. Pilifers small with tufts of short bristles. Maxillary palps white. Labial palps (Fig. 80) very small (1.5× diameter of eye), recurved, small scale tuft on basal segment, second segment much longer than third, upwardly curved, white with some darker scaling towards base, third segment short, straight, thinly covered in white appressed scales. Haustellum with silver scaling on basal portion. Antenna slightly < ¾ length of forewing, scape pale brown, flagellum with broad, dark serrations bearing short white sensillae for 2/3 length, apical third filiform (Fig. 33). ***Thorax***: silvery white, tegulae short, silver white; foreleg dark brown, thin tibial epiphysis, mid legs, and hind legs white, thin tuft of long white scales on hindlegs. Forewing (Fig. 36) moderately broad, elongate, costa gently arched, apex obtusely rounded, termen angled substantially inwards, tornus very obtusely rounded, iridescent silver white, basal half unmarked, medio-dorsal blotch dark brown with a slightly pointed projection basad along medial fold and a projection along termen interrupted by two small patches of white, postmedial streak with lateral projections medially and postmedially, small terminal streak close to apex, cilia silvery white with narrow dark brown internal line at 1/3 and broader terminal line. Hindwings broader than forewings, apex slightly projecting, dorsal margin and termen slightly angled, silver white, unmarked. Ventrally: forewing brown, darker towards costa, hindwing silver.

**Female**. Similar. Forewing length 7.0–8.0 mm, wingspan 15.5–17.0 mm. Antennae filiform throughout.

***Pre-genital abdomen***. White, anal tuft small, white. Tergal spines on posterior part of T2–T7; T8 and sternites weakly sclerotised. Apodemes almost straight; venulae sinuate.

***Male genitalia*** (Fig. 49A, B, C). Uncus short, anteriorly broad, substantially tapering towards posterior apex. Gnathos with broad lateral arms, medial plate with strongly sclerotised, upturned, medial posterior projection. Tegumen band broad, arched, lateral extensions of tegumen slightly shorter than width of tegumen band. Vinculum robust, U-shaped, barely projecting beyond base of valvae. Saccus short and broad. Juxta with U-shaped basal plate, anellus lobes long and tapering, extending slightly beyond costal margin of valvae. Valva broad, costal margin slightly concave, ventral margin obtusely angled medially, apex rounded, a sparse patch of short fine setae on ventral surface of distal half of valva, weak ventral sclerite medially. Sacculus small and indistinct, postero-distal membranous extension large, basally broad, tapering distally. Aedeagus small and thin, tapered distally.

***Female genitalia*** (Fig. 69). Papillae anales short and broad. Apophyses posteriores longer than apophyses anteriores. Ostium broad, almost circular; strongly sclerotised and melanised lamella present. Antrum short and narrow with lateral sclerites. Ductus bursae membranous, broadening towards corpus bursae. Corpus bursae broad ovate with large, lateral, elliptical signum posteriorly.

###### Biology and early stages.

Early stages unknown. Adults found in May and June. Common at mercury vapour light in *Kandelia* (Rhizophoraceae) dominated mangrove forest in the Mai Po Marshes, Hong Kong, China with scattered records at MV light some distance from mangroves in a number of places in Hong Kong including the Lam Kam Valley, Fanling, and Fung Yuen (New Territories), Victoria Peak (Hong Kong Island at 700 m) and records from Lamma and Lantau Islands.

###### Distribution.

China (Hong Kong).

###### Etymology.

*Ptochoryctis caputanatis* sp. nov. – from *caput* (Lat.), head; and *anas* (Lat.), *gen. anatis*, duck. The complex brown postmedial blotch on the forewing of this species resembles to us the profile of the head of a duck. The epithet is a noun in apposition.

##### Ptochoryctis draconella sp. nov.

Taxon classificationAnimaliaLepidopteraAutostichidae

51F3A098-17E5-5F00-ABA7-8D192EF35EF8

https://zoobank.org/BA31539F-1ABA-4ADD-A73B-9C3C34861E97

Figs 10, 37, 50 A, B

###### DNA barcodes.

BIN, BOLD:AGD1579 (Process IDs METAT294-24, METAT295-24).

###### Type material.

Thailand. ***Holotype*** • ♂, NE Thailand: 800 m, Chaiyapumh Dist., Phu Khiao, 2–4.v.1986, Col. M.G. allen leg., fwl 5 mm, specimen no. NHMUK013700425, slide no. NHMUK014332878. ***Paratypes*** (2♂). • 1♂ same collection data as holotype, specimen no. NHMUK013700424; • 1♂, N. Thailand: 1100 m, km 24 on Mae Rim to Samoeng Road, 1.vi.1989, Col. M.G. Allen leg., specimen no. NHMUK010922983, slide no. NHMUK014332879.

###### Diagnosis.

Similar to other *anguillaris* group species but, externally, the postmedial blotch is only narrowly interrupted with white towards the termen (Fig. 37), a character shared with *P. kitchingi*, but the postmedial markings are stronger and more pronounced than in *P. kitchingi*. The male genitalia are similar to those of *P. caputanatis* but the valva of *P. draconella* is narrower distally and the costal margin of the valva is more strongly concave. Additionally, the posterior medial projection of the gnathos points slightly dorsad (Figs 49A, 50A). In *P. kitchingi* the posterior medial projection of the gnathos is substantially longer and points diagonally ventrad (Fig. 51A).

###### Description.

**Male** (Figs 10, 37). Forewing length 5.0 mm, wingspan 11.0 mm. ***Head***: frons with shining white appressed scales, vertex with short tufts of shining white scales laterally from occiput and further tuft of shining white scales on posterior margin of occiput projecting posteriorly. Maxillary palps white. Labial palps small (<2× diameter of eye), closely appressed to eye, small scale tuft on basal segment, second segment much longer than third, upwardly curved, white with some brown scaling towards base, third segment short, straight, thinly covered with white appressed scales. Haustellum with silver scaling on basal portion. Antenna ¾ length of forewing, serrate, scape pale brown, flagellum with broad dark serrations bearing thick white sensillae for 2/3 length, apical part with very small serrations and some sensillae. ***Thorax***: shining white, tegulae short, silver white; foreleg dark brown, thin tibial epiphysis, mid legs and hindlegs white. Forewing (Fig. 37) moderately broad, elongate, costa almost straight, apex obtusely rounded, termen angled inwards, tornus very obtusely rounded; iridescent silver white, basal half unmarked, medio-dorsal blotch with a broad projection pointed basad along medial fold, narrowly interrupted white at termen with a long mediad projection along termen with two white interruptions; postmedial streak narrow and pale brown towards costa, forming a dark brown lateral streak medially which extends to termen. Cilia silver white with a narrow dark brown internal line at 1/3 and broader variegated terminal band. Hindwing broader than forewing, apex projecting, dorsal margin and termen slightly angled, shining white, unmarked. Ventrally, forewing brown, darker towards costa, hindwing silver.

**Female**. Unknown.

***Pre-genital abdomen***. White. Apodemes and venulae almost straight. Tergal spines posteriorly on T2 to T7, slight sclerotisation posteriorly on T8 and on sternites.

***Male genitalia*** (Fig. 50A, B). Uncus short, anteriorly broad, strongly tapered towards posterior apex, anterior margin of dorsal surface strongly emarginated. Gnathos with broad, strongly sclerotised lateral arms, medial plate with strongly sclerotised medial posterior projection, slightly pointing dorsally. Tegumen band broad, lateral extensions of tegumen slightly shorter than width of tegumen band. Vinculum broad, U-shaped, base slightly projecting beyond base of valvae. Saccus small and short. Juxta with small basal plate, anellus lobes broad, extending as far as costal margin of valvae. Valva shorter than distance between apex of uncus and base of vinculum, basally broad, tapering distally, costal margin concave, saccular margin uniformly convex, apex rounded, fine setae distally towards ventral margin. Sacculus indistinct with elongate postero-distal membranous extension. Aedeagus short and comparatively broad with small distal projection.

###### Biology and early stages.

Early stages unknown. Adults have been found in May and June at 800 m and 1100 m.

###### Distribution.

Northern Thailand.

###### Etymology.

*Ptochoryctis draconella* – from draco (Lat.), a dragon, and *ella*, a diminutive, a little dragon. The post-medial markings of this species resemble an oriental dragon. The epithet is a noun in apposition.

##### Ptochoryctis kitchingi sp. nov.

Taxon classificationAnimaliaLepidopteraAutostichidae

FF87BA7D-89F8-570E-BCCA-8312CCE4E7FB

https://zoobank.org/F150FD09-6CB0-47BC-880F-FCC755A79EBA

Figs 11, 38, 51 A, B

###### DNA barcode.

The sequence fragment obtained (Process ID METAT296-24) was too short (301 bp) to qualify for a BIN.

###### Type material.

Thailand: ***Holotype*** • ♂, N. Thailand: 600 m, Phayao Province, km 12.5 Wang Neua to Phayao Road, 22.vii.1990, I.J. Kitching and A.M. Cotton leg., fwl 5 mm, specimen no. NHMUK013700426, slide no. NHMUK014332880, Process ID METAT 296-24.

###### Diagnosis.

Externally this species has the smallest and least strong postmedial markings of any member of the *P. anguillaris* group. In the male genitalia the gnathos has a very long medial projection which extends postero-ventrally at a steep angle (Figs 38, 51A).

###### Description.

**Male** (Figs 11, 38). Forewing length 5.0 mm, wingspan 10.0 mm. ***Head***: Frons worn, vertex with iridescent white scales, occiput with tufts of iridescent white scales laterally, projecting inwards, a collar of broad white scales projecting posteriorly from anterior margin of prothorax. Pilifers with tufts of small bristles, maxillary palps not visible. Labial palps short (<2× length of diameter of eye), closely appressed to eye, basal segment with small tuft of ochreous scales, second and third segments of equal length, with iridescent white scales, second segment strongly recurved, third segment straight. Haustellum with silver scales on basal portion. Antenna ¾ length of forewing, shortly bipectinate, scape pale brown with brassy reflections, flagellum dark brown with short pectinations bearing thick white sensillae for ¾ length. ***Thorax***: with appressed iridescent silver white scales, tegulae short, iridescent silver white. Foreleg with femur and tibia pale brown, short broad tibial epiphysis, tarsus pale brown, distally tipped dark brown, mid legs and hind legs silver white. Forewing elongate, costa almost straight, apex obtusely rounded, termen sharply angled inwards, tornus very obtusely rounded; iridescent silver white, basal half unmarked, medio-dorsal blotch with a rounded projection basad along dorsum, slightly interrupted white along termen, a small, independent, dark spot at termen, postmedial streak very faint at costa with a broad oblique streak beneath which extends to termen. Hindwing strongly projecting at apex, silver white, unmarked. Ventrally, forewings pale brown, darker brown in costal region towards base, hindwings silver white.

**Female**. Unknown

***Pre-genital abdomen***. Silver white. Apodemes curved anteriorly, venulae straight. Tergal spines posteriorly on T2–T7. T8 unsclerotised. Sternites weakly sclerotised.

***Male genitalia*** (Fig. 51A, B) (laterally mounted). Uncus short, broad at base, tapering to a sclerotised point at posterior apex. Gnathos with small, weakly sclerotised lateral arms and a very long, strongly sclerotised, medial projection which extends postero-ventrally at an angle of > 45°. Tegumen band broad, lateral extensions of tegumen shorter than width of tegumen band. Vinculum broad, U-shaped, not projecting beyond base of valvae. Saccus small and short. Juxta with indistinct basal plate, anellus lobes broad, same length as valva. Valva shorter than distance between apex of uncus and base of tegumen, broad throughout, costal margin slightly concave, ventral margin straight, apex rounded, a patch of fine sparse setae on ventral surface towards apex. Sacculus indistinct with elongate postero-distal thickened membranous extension. Aedeagus slightly curved, tapering posteriorly to a broad point.

###### Biology and early stages.

Early stages unknown. The adult was found in July at 600 m.

###### Distribution.

Northern Thailand.

###### Etymology.

This species is named in honour of Dr. Ian Kitching, who is best known for his worldwide expertise in Sphingidae, but who collected a substantial number of species of white Asian xyloryctids in Thailand in the 1980’s and 1990’s, many of which remain undescribed. The epithet is a noun in the genitive case.

##### Ptochoryctis minimella sp. nov.

Taxon classificationAnimaliaLepidopteraAutostichidae

15A4241C-D450-5885-847E-B95CD3796F83

https://zoobank.org/2F5EEA7A-3D25-4575-8467-03F1DE77681B

Figs 12, 39, 52 A, B, 105

###### DNA barcodes.

The sequence fragment obtained (Process ID METAT338-24) was too short (384 bp) to qualify for a BIN.

###### Type material.

Brunei and Malaysian Borneo. ***Holotype*** • ♂, Brunei, Telisai, kerangas forest, 30 m, 13.ii.1982, G.S. Robinson leg., fwl 4.5 mm, specimen no. NHMUK013700444, slide no. NHMUK014332882, process ID METAT338-24. ***Paratypes*** (4♂), • 3♂, Malaysia, Sabah, Gunong Monkobo, 5.84°N, 116.56°E, dipterocarp forest, 945 m, 14–23.viii.1987, K.R. Tuck leg., specimen no. NHMUK010923153, slide no. NHMUK010316403; specimen no. NHMUK010923154, slide no. NHMUK010316860; specimen no. NHMUK010920059, slide no. NHMUK010316859; • 1♂ Brunei, Badas, swamp forest and secondary vegetation, 13.ii.1982, G.S. Robinson leg., specimen no. NHMUK010922984, slide no. NHMUK014332881.

###### Diagnosis.

Externally, the dark postmedial blotch substantially interrupted by white together with the subterminal line with substantial lateral projections (Fig. 39) distinguishes this species. In the male genitalia, the valva is very small and broader than long, the medial plate of the gnathos has two large rounded lateral projections and the uncus is strongly bifid (Fig. 52A).

###### Description.

**Male** (Figs 12, 39). Forewing length 4.5 mm, wingspan 10.0 mm. ***Head***: frons with white appressed scales, vertex with tufts of shortish white scales at sides of occiput pointing inwards, a ruff of fairly short white thin scales on posterior part of occiput pointing posteriorly and some broader white scales also pointing posteriorly from anterior margin of prothorax. Pilifers with small tufts of bristles. Maxillary palps whitish. Labial palps very small (barely longer than diameter of eye), recurved, closely appressed to head, basal segment pale brown, second segment much longer than third, upwardly curved, thinly covered in whitish scales, third segment straight, thinly covered in white appressed scales. Haustellum with silver white scaling on basal portion. Antenna ¾ length of forewing, scape reasonably long with remains of whitish ochreous scaling, flagellum dark, serrate, serrations covered in short sensillae for ~ ¾ of length, apical portion filiform. ***Thorax***: white, tegulae short, white; forelegs brown, thin tibial epiphysis, mid legs and hind legs white, hind legs with sparse tuft of long white scales. Forewing elongate, moderately broad but appearing broader due to long cilia in tornal area, costa gently arched, apex slightly pointed, termen angled substantially inwards, tornus very obtusely rounded, silver white with some iridescence, basal half unmarked, dark medio-dorsal blotch strongly interrupted white, large pointed projection basad and short lateral extension towards termen, joined to a broad, irregular, subterminal line with substantial lateral projections; cilia long and white with a thin dark brown internal line at ~ ½ and a broader brownish grey terminal line. Hindwing as broad as forewing, projecting at apex, greyish white, slightly reticulated pale greyish brown. Ventrally: forewing brown, darker towards costa; hindwing silver.

**Female**. Unknown.

***Pre-genital abdomen***. White, anal tuft small, white. Patches of tergal spines on posterior parts of T2–T7, T8 and sternites unsclerotised. Apodemes straight; venulae slightly curved.

***Male genitalia*** (Fig. 52A, B). Uncus anteriorly broad, strongly bifid towards posterior apex, anterior margin of dorsal surface strongly emarginated. Gnathos with short lateral arms and two broad, rounded, strongly sclerotised lateral posterior projections. Tegumen band broad, lateral extensions of tegumen slightly shorter than width of tegumen band. Vinculum broad, robust, U-shaped, base slightly projecting anteriorly beyond base of valvae. Saccus short and broad. Juxta with broad circular plate, anellus lobes very broad, extending slightly above costal margin of valvae. Valva broad, oval, costal and ventral margins slightly convex, apex rounded with a small sclerotised nodule, weak ventral sclerite extending from ventral margin to apex, ventral surface of valva slightly setose towards apex. Sacculus indistinct, postero-distal thickened membranous extension broad at base, tapering to a blunt point towards inner margin of ventral sclerite. Aedeagus short and curved, tapering to a broad point.

###### Biology and early stages.

Early stages unknown. Adults found in kerangas forest, swamp forest, and dipterocarp forest.

###### Distribution.

Brunei; Sabah, Malaysia.

###### Etymology.

*Ptochoryctis minimella* sp. nov. – from *minimus* (Lat.) meaning very small or smallest. This is one of the smallest species in the genus. The epithet is an adjective in the nominative singular.

##### Ptochoryctis robinsoni sp. nov.

Taxon classificationAnimaliaLepidopteraAutostichidae

163BB0DB-9488-53A2-AB1D-BD9C08518BA7

https://zoobank.org/FD12EEDA-2843-4661-A95C-24D73E311D25

Figs 13, 40, 53 A, B, 86,

###### DNA barcode.

BIN, BOLD:ADR3482 (Process ID METAT010-18).

###### Type material.

Brunei: ***Holotype*** • ♂ Bt Bedawan, LP263, GR343958, ridge dipterocarp forest, 20–24.iv.1988, 520 m, G.S. Robinson leg., fwl 6.5 mm specimen no. NHMUK010920060, slide no. NHMUK010316364, BIN, BOLD:ADR3482. ***Paratype*** • ♂ Sarawak, Gunong Mulu NP, 400 m, 25.v-1.vii.1978, leg. J.E. Marshall, specimen no. NHMUK010922985, slide no. NHMUK010316457.

###### Diagnosis.

This species has the largest dorsal blotch of the members of the *anguillaris* group (Fig. 40). In the male genitalia the straight, sharply angulated, distal margin of the valva is diagnostic (Fig. 53A).

###### Description.

**Male** (Figs 13, 40). Forewing length 5.5–6.5 mm, wingspan 12.0–14.5 mm. ***Head***: frons worn but traces of white scaling, vertex with traces of long white scaling towards base of antenna and traces of tufts of long white scales projecting inwards from sides of occiput, a thin ruff of long white scales projecting posteriorly from posterior margin of occiput overlaying a collar of broad white lamellate scales projecting posteriorly from anterior margin of prothorax. Pilifers small with moderate tufts of bristles, maxillary palps white. Labial palps short (< 1.5× diameter of eye), closely appressed to head, recurved, basal segment with small tuft of whitish scales, second segment substantially longer than third, white, third segment straight, white appressed scales. Haustellum with silver scaling on basal half. Antenna ¾ length of forewing, bipectinate, scape damaged by grease, flagellum dark brown, pectinations covered with white sensillae for ~ ¾ of length, apical portion filiform. ***Thorax***: with remains of lamellate white scaling, tegulae short, white; forelegs dark brown, moderate tibial epiphysis, mid and hind legs white, hind legs with thin tuft of long white scales. Forewing broad and elongate, costa rounded at base, thereafter straight but rounding at apex, apex rounded, termen pointing substantially inwards although the appearance of this is reduced by the long cilia towards the tornus, tornus obtusely rounded, silver white, iridescent, basal third unmarked, large, dark brown and sharply angulated medio-dorsal blotch from dorsum to middle, continuing from middle to termen, subterminal line narrow at costa, substantially broadening towards and interrupted with white at termen; cilia long, very long towards tornus, white, a dark brown internal line and a broad, greyish terminal line. Hindwing as broad as forewing, projecting at apex, iridescent silvery white, unmarked. Ventrally: forewings silver with some brown scaling, veins ochreous; hindwings silver, veins ochreous.

**Female**. Unknown.

***Pre-genital abdomen***. White, anal tuft white. Patches of tergal spines on posterior parts of T2–T7; T8 and sternites weakly sclerotised. Apodemes curved; venulae straight.

***Male genitalia*** (Figs 53A, 53B, 86). Uncus anteriorly broad, strongly tapering towards posterior apex, anterior margin of dorsal surface strongly emarginated. Gnathos with long broad lateral arms, medial plate with a single, strongly sclerotised, medial posterior projection. Tegumen band broad, lateral extensions of tegumen same length as width of tegumen band. Vinculum narrow, barely projecting beyond base of valvae. Saccus small. Juxta with U-shaped basal plate, anellus lobes widely spaced apically, not extending as far as costal margin of valva. Valva broad apically, tapering towards apex. Costal, ventral and distal margins almost straight, distal margin angled between costal and ventral margins, elongate ventral sclerite. Sacculus broad with elongate postero-distal membranous thickening. Aedeagus slightly curved.

###### Biology and early stages.

Early stages unknown. Adults found in April and July from 400–520 m. One specimen recorded in ridge dipterocarp forest.

###### Distribution.

Brunei, Malaysian Borneo (Sarawak).

###### Etymology.

*Ptochoryctis robinsoni* sp. nov. – named in honour of the late Gaden Robinson, an eminent microlepidopterist who was for many years a curator of microlepidoptera at the NHMUK and, among many other things, a specialist on the microlepidoptera of South East Asia. The epithet is a noun in the genitive case.

##### Ptochoryctis sundarbanica

Taxon classificationAnimaliaLepidopteraAutostichidae

Sterling & Singh, 2025

1368749A-D26A-578B-B9A1-83F703D374B0

Figs 14, 41, 54 A, B

###### DNA barcodes.

BIN, BOLD:AGD1578 (Process IDs ZSILE001-25, ZSILE002-25)

###### Note.

This species was described in Sterling and Singh (2025). Since publication, a further adult specimen has been recorded, (Jahir Rayhan pers. comm., det. Jahir Rayhan, identity confirmed by MJS (Fig. 32)). The collection details of this specimen are: Boiddamari, Mongla, Sundarbans, Bangladesh, 22.399 89.655, 13.vii.2025, at light, secondary forest, leg. Jahir Rayhan.

The holotype and paratype have been DNA barcoded and this data has been included in our results.

#### The *chalazopa* group

This group is a supported sub-clade. It consists of two closely related species, one known from Java, Brunei and Malaysia and the other known from Thailand. The former species is associated with rubber (*Hevea brasiliensis* Müll. Arg.).

##### Ptochoryctis chalazopa

Taxon classificationAnimaliaLepidopteraAutostichidae

Meyrick, 1920

7343E1E8-BDCC-546D-8060-AFE831D86269

Figs 15, 16, 55 A, B, 70, 71, 92, 109.

Ptochoryctis chalazopa Meyrick, 1920: 321.

###### DNA barcodes.

BIN, BOLD:ADR3476 (Process IDs METAT041-18, METAT297-24).

###### Type material.

Indonesia: ***Holotype*** • ♀ Java, Buitenzorg [now Bogor], ex Hevea, January; fwl 7 mm, specimen no. NHMUK010219735, B. M. genitalia slide No. 4880.

###### Additional material examined.

• 1♂ Malaysia, Sabah, Kipandi Butterfly Park, 36 Km from KK via the Penampang – Tambunan Rd., 5.87°N, 116.25°E ca. 700 m, 13.iv.2013, Light Trap UV, Leg. A. Giusti, M.V.L. Barclay, B.H. Garner & H. Mendel, specimen no. NHMUK010897820, slide no. NHMUK014332876, Process ID METAT297-24; • 1♂ Brunei: 300’ Rampayoh R., LP 195, GR 960785, lowland dipterocarp forest, 21–24.ix.1992, GS Robinson leg., specimen no. NHMUK010923010, slide no. NHMUK010316459; • 2♂ Brunei, 60 m, Lamunin, Sg Burong water tanks, disturbed lowland forest, 17–30.ix.1992, GS Robinson leg., specimen nos. NHMUK010923009, slide no. NHMUK010316404, Process ID METAT041-18; NHMUK013700859; • 1♀, 2♂ Brunei, Seria, Swamp Forest, 10’ 18.iv.1988, GS Robinson leg., specimen nos. NHMUK013700860; NHMUK013700861; NHMUK013700863; • 1♂ Brunei, Sungai Burong, 11.xii.1993, G. Ping leg., specimen no. NHMUK013700864; Brunei, Panaga, secondary veg., 5.i.1980, R. Fairclough leg., specimen no. NHMUK013700865; • 1♀ 13 km w of Bandar SB, Kampong Katimanar, secondary forest, 24.vi.1991, leg. G.S. Robinson, specimen no. NHMUK010219793, slide no. NHMUK010316877; • 1♀ Brunei, Seria, Swamp Forest, 10’ 18.iv.1988, GS Robinson leg., specimen no. NHMUK013700862, slide no., NHMUK014333417.

###### Diagnosis.

*P. chalazopa* and *P. marmorella* are similar but in *P. marmorella* the base of the costa is strongly angulated whereas in *P. chalazopa* it is smoothlycurved. In the male genitalia, the valva of *P. marmorella* is longer and more pointed than the valva of *P. chalazopa* (Figs 55A, 56A).

**Male** (Figs 15, 16). Forewing length 7.0–7.5 mm, wingspan 16.0–16.5 mm. ***Head***: frons with white, slightly shaggy scales, vertex with metallic white appressed scales, a tuft of longer cream scales at each side, a ruff of long cream scales from posterior part of occiput pointing posteriorly, overlaying a collar of broad flat metallic white scales on anterior margin of prothorax also pointing posteriorly. Pilifers with small tufts of bristles; maxillary palps white. Labial palps short (1.5× diameter of eye), recurved, tightly appressed to head, basal segment ochreous brown, second segment long and thin, substantially longer than third segment, ochreous, third segment thin and pointed, silver metallic. Haustellum with basal portion thinly scaled metallic white. Antenna ¾ length of forewing, scape thickly scaled metallic white, basal half of flagellum thickly scaled cream on dorsal surface, remainder brown, pectinations dark, thickly covered with short white sensillae, reducing at ¾, apical portion filiform. ***Thorax***: anterior third iridescent white, remainder bluish black, tegulae white with some darker speckling; foreleg dark fuscous, tarsus intermixed with brown, moderate tibial epiphysis, mid and hind legs white with longer white scales. Forewing broad, costa smoothly arched at base, straight thereafter, apex rounded, termen slightly angled inwards, tornus obliquely rounded (without cilia) but slightly rounded including cilia, forewing cilia very long, longer towards tornus forming dorsal crest in adult resting posture (see Fig. 34); white, a broad band of black irroration suffused pale grey rising obliquely from dorsum near base and running through disc above middle to a large roundish blotch occupying most of wing beyond cell but not extending to margins, posteriorly suffused blackish, a triangular blackish spot on dorsum approx. middle, and two rather inwards-oblique streaks of blackish irroration between this and posterior blotch, a terminal series of small groups of black scales: cilia white, a black basal line, dark grey median and subapical lines. Hindwing as broad as forewing, apex rounded, basally white, distally grey, cilia white. Ventral surface with forewings scaled dark brown, hindwings white.

**Female**. Similar to male.

***Pre-genital abdomen***. Greyish white. Patches of tergal spines on T2–T7 (patch on T7 somewhat reduced); T8 and sternites unsclerotised. Apodemes almost straight; venulae straight.

***Male genitalia*** (Figs 55A, 55B, 92, 109). Uncus short, broad throughout, slightly emarginate medially, anterior margin of dorsal surface strongly emarginate, posterior apex spatulate. Gnathos with broad lateral arms, medial plate an evenly curved band, projecting posteriorly. Tegumen band arched and broad, lateral extensions of tegumen slightly shorter than width of tegumen band. Vinculum robust, short, and broad, posteriorly narrowing slightly, U-shaped, slightly projecting beyond base of valvae. Saccus short and broad. Juxta with almost circular basal plate, anellus lobes basally broad, uniformly tapering, extending above costal margin of valva. Valva broad throughout, costal margin slightly sinuate, ventral margin slightly curved, distal margin angled inwards, apex bluntly pointed, small sclerotised nodule at apex of valva, weak ventral sclerite medially and postmedially. Sacculus indistinct, thickened postero-distal membranous extension short and fairly narrow (Fig. 109). Aedeagus almost straight with small distal projection.

***Female genitalia*** (Figs 70, 71). Papillae anales short and broad. Apophyses posteriores longer than apophyses anteriores. S8 with posterior medial emargination with a row of bristles on emargination. Ostium circular and narrow. Antrum sclerotised posteriorly, sclerotised section same length as apophyses posteriores. Ductus bursae membranous, broadening towards corpus bursae. Corpus bursae pyriform, elongate, without signum.

###### Early stages and biology.

Bred in January from larva feeding in bark of *Hevea* (Euphorbiaceae) making curious webs (Meyrick 1920: 321). Adults found in January, April–June, September, and December.

###### Distribution.

Java (Indonesia), Brunei, Sabah, and Selangor (Malaysia).

###### Note on Meyrick’s etymology.

*chalazopa* Meyrick, 1920 – from *chalaza* (Gr.), hail, but also any small round bump, wart, pimple, or knot; and *ops* (gr.), face. This name is a reference to the blueish-black pimple like irroration which covers a significant part of the forewings of this species.

##### Ptochoryctis marmorella sp. nov.

Taxon classificationAnimaliaLepidopteraAutostichidae

B251D7EF-D203-59DF-AFFD-E3CF1729AE47

https://zoobank.org/3A01DECD-982A-4758-B348-062DF4023E55

Figs 17, 56 A, B, 95

###### DNA barcodes.

BIN, BOLD:AGD1582 (Process ID METAT298-24).

###### Type material.

Thailand: ***Holotype*** • ♂, Central Thailand, Khao Yai NP, 800 m, 28.v.1988, leg. Col. M.G. Allen, fwl 6 mm, specimen no. NHMUK013700427, slide no. NHMUK014332877.

###### Diagnosis.

See diagnosis for *P. chalazopa*.

**Male** (Fig. 17). Forewing length 6.0 mm, wingspan 14.0 mm. ***Head***: frons with remains of white scales, vertex completely worn apart from collar of broad, flat, rich white scales from anterior margin of prothorax pointing posteriorly. Pilifers small with small tufts of bristles; maxillary palps white. Labial palps very small (1.5× diameter of eye), closely appressed to head, recurved, without scales, second segment slightly longer than third, curved, third segment straight. Haustellum small, base covered in silver white scales. Antenna < ¾ length of forewing, bipectinate, scape with traces of white scales remaining, flagellum pale brown, traces of white scales, bipectinate for more than 1/2 length, pectinations fairly long, pale brown, covered in short white sensillae, apical portion filiform. ***Thorax***: with some white scales peripherally, tegulae short, white; forelegs brown, broad tibial epiphysis, mid and hind legs with some white scaling. Forewing broad, costa substantially arched at base, apex gently rounded, termen angled inwards slightly, tornus obtusely angled, dorsum rounded at base, forewing white and slightly iridescent, a large sub-medial dark blotch and large dark blotch postmedially extending to termen, remains of dark scaling in cilia. Hindwings narrower than forewings, apex rounded, termen angled slightly inwards, greyish white distally, white basally, cilia very long, brilliant white. Ventrally, costal half of forewing with dark scaling, whitish dorsally, hindwings white.

**Female**. Unknown

***Pre-genital abdomen***. White, anal tuft small, white. Patches of tergal spines on posterior parts of T2–T7; T8 and sternites unsclerotised. Apodemes straight.

***Male genitalia*** (Fig. 56A, B). Uncus short, broad throughout, emarginate medially, posterior apex spatulate, anterior margin of dorsal surface strongly emarginate. Gnathos with long broad lateral arms, medial plate an evenly curved band, projecting posteriorly. Tegumen band arched and broad, lateral extensions of tegumen approximately same length as width of tegumen band (Fig. 95). Vinculum robust, short, and broad, U-shaped, significantly projecting beyond base of valvae. Saccus short and broad. Juxta with circular basal plate, anellus lobes broad, extending well above base of costal margin of valva. Valva broad, costal margin almost straight, ventral margin slightly curved, distal margin curved, apically pointed, small sclerotised nodule at apex of valva, very weak ventral sclerite medially and postmedially. Sacculus indistinct, thickened postero-distal membranous extension short and fairly narrow. Aedeagus almost straight with a small distal projection.

***Female genitalia***. Unknown.

###### Biology and early stages.

Early stages unknown. Adult recorded in May at 800 m elevation.

###### Distribution.

Central Thailand.

###### Etymology.

*Ptochoryctis marmorella* – from *marmoreus* (Lat.) meaning marbled, from the marbled appearance of the forewing. The epithet is an adjective in the nominative singular.

#### The *corticivora* group

This group of three species is recovered as a supported subclade in Fig. 3 but not in the other analyses. The antennae of these species are serrate and in the male genitalia they share a long, thin, thickened postero-distal membranous extension of the sacculus.

##### Ptochoryctis corticivora

Taxon classificationAnimaliaLepidopteraAutostichidae

(Meyrick, 1934) comb. nov.

0E0A506F-5DC1-5631-A662-C18753C780ED

Figs 18, 19, 57 A, B, 72, 110

Deloryctis corticivora Meyrick 1934: 464.

###### DNA barcode.

BIN, BOLD:ADR4228 (Process ID METAT018-18).

###### Type material.

Indonesia: ***Lectotype*** • ♂ specimen no. NHMUK010922382, slide no. JFGC 7691; Java, Telawa, ex *Schleichera trijuga*, L.G.E. Kalshoven leg., fwl 5 mm. Lectotype designated by Gates Clarke (Clarke 1955b: 421) who notes that Meyrick’s description of the type specimens as being from Telawa is an apparent lapsus, since all three of the five original specimens in the Meyrick collection are labelled Seneng, Java, K. bred 8.32 although Kalshoven bred much material from Telawa so the specimens may be mislabelled. ***Paralectotype*** • 1♀, Java, Telawa, ex *Schleichera trijuga*, L.G.E. Kalshoven leg., specimen no. NHMUK013700901, BM genitalia slide no. 5544.

###### Additional material examined.

• 1♂ Brunei, KG. Kapok, 22.xi.1992, G. Ping leg., specimen no. NHMUK010922990, slide no. NHMUK010316460, BIN, BOLD:ADR4228; • 1♂ Brunei, KG. Kapok, 7.vi.1992, G. Ping leg., specimen no. NHMUK010922991; slide no. NHMUK010316461, BIN, BOLD:ADR4228; • 1♂ E. Sabah, Lahad Datu, Danum Valley Field Centre, 10–11.v.1989, K.R. Tuck leg., specimen no. NHMUK013700897 (Robinson et al. 1994:65, Plate 9 Fig. 10); • 1♂ Brunei: 60 m Lamunin, Sg Burong water tanks, disturbed lowland forest, 15–20.iv.1993, G.S. Robinson leg., specimen no. NHMUK013697437, slide no. NHMUK014333407; • 1♂ Brunei, KG. Kapok, 6.ix.1991, leg. G. Ping, det. J-C Sohn, specimen no. NHMUK013700899, BM Genitalia slide no. 32934, • 1♂ Brunei, Sungai Burong, 18.ix.1993 leg. G. Ping, specimen no. NHMUK013700900; • 1♂ Brunei, 3 km WSW of Muara, Kampong Kapok, edge of mangrove forest, 1 m, i–ii. 1992 E.W. Classey leg., specimen no. NHMUK013700905; • 1♂ Sarawak, Kuching, Semongok, 3–9.ii.1976, E.W. Classey leg., specimen no. NHMUK013700906; • 1♀ Malaya, Selangor, 3/9/–12-57, larva eating bark of rubber, C.I.E Coll. No. 15792, specimen no. NHMUK010219791, slide no. NHMUK014333408; • 1♀ Malaya, Selangor, 3/9/–12-57, larva eating bark of rubber, C.I.E Coll. No. 15792, specimen no. NHMUK013700898, BM Genitalia slide no. 5021; • 1♀ Rubber Plantation, Selangor, 3/9/–12-57, R.R.I. Malaya, C.I.E. Coll. No. 15792, bark eating larva, BM Genitalia slide no. 4881; • 1♀ Malaya, Selangor, 3/9/–12-57, larva eating bark of rubber, C.I.E. Coll. No. 15792, specimen no. NHMUK010219791, slide no. NHMUK014333408; • 1♀ Brunei, 3 km WSW of Muara, Kampong Kapok, edge of mangrove forest, 19–21.vii. 1991, G.S. Robinson leg., specimen no. NHMUK013700907, slide no. NHMUK014333414; • 1♀ Brunei, Telisai, kerangas forest, 13.ii.1982, G.S. Robinson leg., specimen no. NHMUK013700909; Rubber Plantation, Selangor, 3/9/–11–57, R.R.I. Malaya, C.I.E. coll. No 17580; 4 specimens, antennae and abdomens missing, rubber plantation, Selangor, R.R. I. Malaya, 3/9/–11.57, 2× C.I.E. Coll. No. 16489, 2× C.I.E. Coll. No.17580.

###### Diagnosis.

The large brown marking covering most of the distal half of the wing against the intense white ground colour serves to distinguish this species from all other members of *Ptochoryctis*. In the male genitalia, the apical part of the valva is long and tapering and a narrow transtilla is present. In the female genitalia a flat, diamond shaped signum is present. BOLD, BIN: BOLD:ADR4228.

###### Description.

**Male** (Figs 18, 19). Wingspan 11.5–15.0 mm, forewing length 5.0–6.5 mm. ***Head***: Frons with appressed white scales, vertex with appressed white scales overlaid by two tufts of long yellowish white scales from sides, pointing inwards and posteriorly, themselves overlaying a collar of broad flat metallic white scales from anterior margin of prothorax, pointing posteriorly. Pilifers small and cylindrical with a few bristles; maxillary palps white. Labial palps long (> 2.5× diameter of eye), strongly recurved, tightly appressed to head, white, basal segment with small scale tuft, second segment strongly curved, thin, substantially longer than third segment, third segment thin, pointed. Haustellum with thin silver scaling on basal portion. Antenna ¾ length of forewing, scape dark brown, flagellum brown, with small serrations covered in small white sensillae for most of length, apical portion filiform. ***Thorax***: white, tegulae short, white; foreleg with femur white, tibia and tarsus dark brown, moderate tibial epiphysis, mid legs and hind legs white, both with small tufts of long white scales. Forewing broad, costa gently arched, apex obtusely rounded, termen very slightly angled inwards, tornus angled at almost 90°, basal half enamel white, dark brown marking commencing medially, extending from dorsum almost to costa and covering much of distal half of wing, narrow angulated subterminal line; cilia mostly brown, but white towards apex with dark brown internal line near base and brown terminal line continuing to apex. Hindwing almost as broad as forewing, apex slightly projecting, rounded, white with a large rich brown postmedial patch, brown internal and terminal lines in apical half of cilia. Ventrally: forewings dark brown apart from silver white area near dorsum, hindwings similar to dorsal surface.

**Female**. Similar to male. Wingspan 17.0–19.0 mm, forewing length 8.5 mm.

***Pre-genital abdomen***. Silvery white, anal tuft silvery white. Patches of tergal spines on posterior parts of T2–T7; T2–T7 also sclerotised anteriorly; sternites well sclerotised. Apodemes and venulae straight.

***Male genitalia*** (Figs 57A, 57B, 110). Uncus anteriorly broad, tapering slightly towards posterior apex, posterior apex rounded, slightly bilobed, dorsal surface strongly emarginated. Gnathos with long lateral arms and rounded, slightly indented, medial plate projecting posteriorly. Tegumen band arched and broad, lateral extensions of tegumen same length as width of tegumen band. Vinculum V-shaped, robust, projecting substantially beyond base of valvae. Saccus narrow. Juxta with small circular plate, anellus lobes projecting level with top of vinculum. Narrow transtilla present. Valva broad basally, strongly tapering towards apex, costal margin slightly concave, ventral margin curved basally, otherwise straight, small thorn-like apical process present. Sacculus ½ width of valva, long, indistinct, with narrow elongate postero-distal membranous thickening. Aedeagus almost straight with slight distal projection.

***Female genitalia*** (Fig. 72, see also Bradley, 1959). Papillae anales short and broad. Apophyses posteriores slightly longer than apophyses anteriores. S8 with posterior medial emargination with a row of bristles on emargination. Ostium broad and circular. Antrum short, sclerotised posteriorly, sclerotised section shorter than apophyses posteriores, tapering anteriorly. Ductus bursae short, membranous, broadening towards corpus bursae. Corpus bursae small, pyriform, with large, weakly sclerotised diamond-shaped signum, sometimes with a lateral ridge.

###### Biology and early stages.

Bred in August and September from larvae feeding on bark of *Schleichera trijuga* (Sapindaceae) (Meyrick 1934: 464) (*S. trijuga* Willd is a synonym of *Schleichera oleosa* (Lour.) Oken) and from bark of *Hevea brasiliensis* (Euphorbiaceae) (Bradley 1959; Robinson et al. 2001). Adults found in lowland forest, edge of mangrove forest, kerangas forest and rubber plantations at elevations of 0–100 m in January, February, April, June, July, September, and November.

###### Distribution.

Java, Indonesia; Brunei; Malaysia (Sarawak, Sabah, and Selangor).

###### Note on Meyrick’s etymology.

*corticivora* (Meyrick, 1934) – from *cortex* (Lat.), bark; and *voro* (Lat.), devour. The type material was found as larvae feeding on the bark of *Schleichera*.

###### Remarks.

In each of the analyses presented in Figs 1, 2, 3, 4 above, *Deloryctis corticivora* Meyrick, 1934 fell within the *Ptochoryctis* clade. Accordingly, we combine this taxon as *Ptochoryctis corticivora* (Meyrick, 1934), comb. nov. and it is transferred from the Depressariidae to the Xyloryctidae. Bradley (1959) correctly notes the superficial similarities between this species and *P. chalazopa*. However, these two species are 9.6% pairwise divergent by DNA barcode and they do not form a sub-clade within *Ptochoryctis*, so there is currently no basis to suggest that they are closely related species.

##### Ptochoryctis blanchella sp. nov.

Taxon classificationAnimaliaLepidopteraAutostichidae

D35F4EDB-99C1-540B-B446-8EEBC2DC16FC

https://zoobank.org/D9742824-FC02-482C-9D84-B0A454DF113A

Figs 20, 58 A, B, 82, 108

###### DNA barcodes.

BIN, BOLD:AGD1580 (Process IDs METAT292-24, METAT293-24, METAT002-18).

###### Type material.

Thailand**. *Holotype*** • ♂ Thailand, Chiang Mai, Chiang Dao, San Pakia RFD Watershed Station, 1450 m, 28.iv-1.v.1994, I.J. Kitching et al. leg., fwl 4.5 mm, specimen no. NHMUK013700101, slide no. NHMUK014331356, Process ID METAT292-24; ***Paratypes*** (3♂): • 1♂ same collection data as holotype, specimen no. NHMUK013700423, Process ID METAT293-24; • 2♂ NE Thailand, Phu Khieo, Chaiyapumh District, 800 m, 2–4.v.1986, M.G. Allen leg., specimen no. NHMUK010922980; slide no. NHMUK010316402, Process ID METAT002-18; specimen no. NHMUK010922979, slide no. NHMUK010316452.

###### Diagnosis.

This is the only species of white, unmarked *Ptochoryctis* with serrate rather than bipectinate antennae in the male. In the male genitalia, the combination of the strongly melanised lateral posterior projections of the medial plate of the gnathos coupled with the strongly tapering, almost rectangular uncus is diagnostic.

**Male** (Figs 20, 82). Forewing length 4.5–5 mm, wingspan 11.0–11.5 mm. ***Head***: frons with appressed white scales, vertex with small tuft of long white scales pointing away from base of antennae, two crests of long white scales pointing inwards and posteriorly from posterior margin of occiput, towards a collar of flat broad white scales pointing posteriorly from anterior margin of prothorax. Pilifers very small with small tufts of bristles; maxillary palps whitish. Labial palps long (over 2.5 x diameter of eye), strongly recurved, basal segment with small tuft of ochreous scales, second segment longer than third, strongly curved, ochreous mixed with some white, third segment slightly curved, thinly covered in white appressed scales. Haustellum with silver scaling on basal half. Antenna just > ¾ length of forewing, scape white, flagellum cream and ochreous banded throughout with short broad brown serrations bearing short sensillae for ~ ¾ of length, apical portion filiform (Fig. 82). ***Thorax***: off white, tegulae short, off white; foreleg brown, long thin tibial epiphysis, mid and hind legs beige white, hind legs with thin tuft of long cream scales. Forewing moderately broad, costa almost straight, curving inwards just before termen, apex pointed, termen sharply angled inwards, tornus obtusely rounded, apex of hindwing pointed; dull white, unmarked, scattered ochreous tipped scales postmedially and particularly towards tornus and a patch of thicker white scaling on dorsum also containing some ochreous tipped scales, cilia white with a faint line of ochreous scales at ~1/2 and a greyish ochreous terminal line. Hindwing slightly less broad than forewing, off white, unmarked except for an indistinct patch of brown scales towards the margin between base and CuA_2_. Ventrally: small patch of dark brown scales towards base of forewing between costa and Sc, forewing veins lined with ochreous scales, hindwing white.

**Female**. Unknown.

***Pre-genital abdomen***. White, small white anal tuft. Large patches of tergal spines on posterior half of T2–T7; T8 and sternites weakly sclerotised. Apodemes curved, venulae straight.

***Male genitalia*** (Figs 58A, 58B, 108). Uncus short, anterior margin of dorsal surface weakly emarginate, broad at base, posteriorly almost rectangular, barely tapering towards posterior apex. Gnathos with moderately sclerotised lateral arms, medial plate with two large, curved, strongly melanised lateral posterior projections. Tegumen band broad, lateral extensions of tegumen slightly shorter than width of tegumen band. Vinculum robust, V-shaped, projecting anteriorly beyond base of valvae. Saccus short. Juxta with rectangular basal plate, anellus lobes broad, not reaching as far as top of vinculum. Valva broad at base, tapering distally to a pointed apex, costal margin straight, ventral margin convex, sparse fine setae on distal half of ventral surface of valva, thin ventral sclerite medially towards ventral margin. Sacculus large but indistinct, long thin postero-distal thickened membranous extension reaching to apex of ventral sclerite (Fig. 108). Aedeagus almost straight, tapering posteriorly to a broad point.

###### Biology and early stages.

Early stages unknown. Adults recorded in late April/early May at 800 and 1450 m.

###### Distribution.

Northern Thailand.

###### Etymology.

*Ptochoryctis blanchella* sp. nov. – from *blanche*, an old English or old German word meaning white or fair. This is an almost unmarked white species.

##### Ptochoryctis fuscilinea sp. nov.

Taxon classificationAnimaliaLepidopteraAutostichidae

F09B0F69-C504-5985-86EF-63D393658761

https://zoobank.org/54328B27-A49B-47BA-AEED-4F3F6AC1FEA9

Figs 21, 59 A, B, 73, 91

###### DNA barcodes.

BIN, BOLD:AHC2564 (Process IDs BSNHM1690-25, BSNHM1785-25, BSNHM4264-25).

###### Type material.

Malaysia: ***Holotype*** • ♂, West Malaysia, Fraser’s Hill, Semantan Bungalow, 1250 m, 4–9.viii.1986, G.S. Robinson leg., fwl 6 mm, specimen no. NHMUK013701080, slide no. NHMUK014332884; ***Paratypes*** (1♂, 4♀), • 1♂, same collection data as holotype, specimen no. NHMUK013701078, slide no. NHMUK014332676; • 2♀ West Malaysia, Fraser’s Hill, Jeriau Road, 1140 m, 5–12.viii.1986, G.S. Robinson leg., specimen no. NHMUK013701076, slide no. NHMUK014332885; specimen no. NHMUK013700856; • 2♀ West Malaysia, Fraser’s Hill, Gap Road, 1190 m, 8–10.viii.1986, G.S. Robinson leg., specimen no. NHMUK013700850, slide no. NHMUK014333406; specimen no. NHMUK013700808.

###### Diagnosis.

Externally the broad submedial fuscous streak on the forewing which terminates close to the tornus is diagnostic. Also, this is one of only three species apart from some members of the *anguillaris* group in which the male antenna is serrate as opposed to bipectinate. The male genitalia are similar to those of *P. eremopa*, but in *P. fuscilinea* the costal margin of the valva is almost straight and the apex of the valva is pointed whereas in *P. eremopa* the costal margin of the valva is concave and the apex is rounded. Also, the lateral posterior projections of the gnathos are broader in *P. fuscilinea* than in *P. eremopa* and the emargination of the posterior margin of the gnathos is shallower in *P. fuscilinea* (Figs 44A, 45, 46A, 59A).

###### Description.

**Male** (Fig. 21). 13.5 mm. ***Head***: Frons iridescent white with brassy scaling laterally, vertex with tufts of iridescent white scales laterally on occiput and two further broad tufts of white scales, some tipped dark brown, from posterior margin occiput pointing posteriorly, overlaying a collar of white scales on anterior margin of prothorax, pointing posteriorly. Pilifers with thick whitish bristles. Maxillary palps white. Labial palps long (> 2.5× diameter of eye), strongly recurved, basal segment golden brown with a slight tuft, second segment curved, outer lateral and ventral surfaces golden brown, inner lateral and dorsal surfaces white, third segment white, second segment longer than third. Haustellum with silver scaling at base. Antenna > ¾ length of forewing, serrate, scape and pedicel white, flagellum with white scaling on dorsum to ¼, finely serrate for ¾ length, serrations covered in white sensillae, apical part filiform. ***Thorax***: white, mottled dark brown, tegulae short, white, foreleg brown, small tibial epiphysis, mid legs and hind legs white with long white scale tufts. Forewing broad, costa gently arched, apex rounded, termen angled inwards, tornus obtusely angled, iridescent white with scattered dark brown scaling, a broad, dark brown, sub-dorsal streak from ¼ almost to termen, an interrupted dark brown subterminal line from costa almost to dorsum, cilia white with dark brown line at 1/3 and broader grey brown terminal band. Hindwings with rounded, slightly projecting, apex, white. Ventrally: forewings pale brown, dark brown towards costa, hindwings white, unmarked.

**Female**. Similar to male, slightly larger, 14.0–14.5 mm, antenna filiform throughout.

***Pre-genital abdomen***. White. Anal tuft white. Tergal spines on posterior parts of T2 to T7. T8 and sternites not sclerotised.

***Male genitalia*** (Figs 59A, 59B, 91). Uncus short, anteriorly broad, tapering substantially towards posterior apex, anterior margin of dorsal surface strongly emarginate. Gnathos with strongly sclerotised lateral arms, medial plate with two broad, strongly sclerotised, lateral posterior projections. Tegumen band broad, lateral extensions of tegumen slightly shorter than width of tegumen band. Vinculum robust U-shaped, somewhat projecting beyond base of valvae. Saccus short and broad. Juxta with broad basal plate and broad anellus lobes extending almost to costal margin of valva. Valva with costal margin almost straight, ventral margin gently convex, pointed apically and with small process at apex, short fine setae on ventral surface towards apex, thin ventral sclerite medially towards ventral margin. Sacculus broad with long thin postero-distal thickened membranous extension which extends beyond ventral sclerite. Aedeagus small, almost straight.

***Female genitalia*** (Fig. 73). Papillae anales short and broad. Apophyses posteriores same length as apophyses anteriores, both short. Posterior margin of S8 emarginate. Thin, pointed lamella postvaginalis above ostium. Ostium small and circular. Antrum short and narrow, lightly sclerotised posteriorly. Ductus bursae thin and membranous throughout. Corpus bursae round, membranous, without signum.

###### Biology and early stages.

Early stages unknown. Adults have been found in August at between 1140 and 1250 m elevation.

###### Distribution.

Peninsular Malaysia.

###### Etymology.

*Ptochoryctis fuscilinea* from *fuscus* (lat.) dusky and *linea* (Lat.) a line. From the dark submedial line on the forewing of this species. The epithet is an adjective in the nominative singular.

##### Ptochoryctis sp.

Taxon classificationAnimaliaLepidopteraAutostichidae

7C09018F-AAB7-51AC-BF91-E3141BF4E16B

###### DNA barcode.

BIN, BOLD:ADJ0350

###### Note.

INDOBIOSYS-CCDB29857-B07 (Process ID INFCO10279-17) is a specimen from Indonesia, Java Barat, Cidahu, Halimun-Salak NP, Sg. Cibojang, LF Tower, -6.732, 106.71, 1100 m, collected by Wolfram Mey and deposited at the Museum Zoologicum Bogoriense, Bogor. The species is within the *P. corticivora* group (*P. corticivora* is also known from Java). We examined the image of the specimen on BOLD. It is similarly marked to *P. fuscilinea*, but the markings are stronger, with a dark brown metathorax dorsally, being irregularly marked dark brown in the dorsal area and having a row of dark brown subterminal blotches. On BOLD (03/12/2025) the DNA barcode is 3.43% p-distant to that of *P. fuscilinea* (BOLD:AHC2564; NHMUK013701080; BSNHM4264-25).

#### Ungrouped species

##### Ptochoryctis acrosticta

Taxon classificationAnimaliaLepidopteraAutostichidae

Meyrick, 1906

B22BEC64-74AB-59E7-8F4F-EE1D02F6243D

Figs 22, 60 A, B, 74

Ptochoryctis acrosticta Meyrick, 1906: 403.

###### DNA barcode.

N/A.

###### Type material.

***Lectotype*** Sri Lanka • ♂ specimen no. NHMUK010920065; slide no. JFGC 7690; Hambantota, Ceylon. JP. 04; fwl 4 mm. Lectotype designated by Gates Clarke (Clarke 1955b: 493). • 2♂, ***paralectotypes***, Puttalam, Ceylon, Pole .10.04, specimen nos. NHMUK013700902; NHMUK013700982 (both specimens without abdomens); • 1♂, ***paralectotype*** Ceylon, JP .04 specimen no. NHMUK013700983; • 1♀, ***paralectotype***, Puttalam, Ceylon, Pole, .2.04 specimen no. NHMUK013700904, slide no. NHMUK014333409.

###### Additional material examined.

• 2♂, Rambukkhana [more commonly spelt Rambukkana], Ceylon, A., 6.06, specimen nos. NHMUK013700984; NHMUK013700985; • 1♂, Palgahaucla [Polgahawela], Ceylon, JP, .02, specimen no. NHMUK013700987; • 2♂, Poyuhawelle [likely to be a different spelling of Polgahawela], Ceylon, GCA, .4.05, specimen nos. NHMUK013700988; NHMUK013700989; • 1♂, Putlam 14, Pole, 1894, through Hampson, specimen no. NHMUK013700903; • 1♂, Puttalam, Ceylon, Walsingham Coll., specimen no. NHMUK010219705; • 1♀, Rambukkhana, Ceylon, A., 6.06, specimen no. NHMUK013700986.

###### Diagnosis.

The small dark sub-apical spot on the forewing distinguishes this species from the other substantially white species of *Ptochoryctis*. In the male genitalia, this is the only substantially white species of *Ptochoryctis* with a large process projecting from the tip of the valva.

###### Description.

**Male** (Fig. 22). Forewing length 4.0 mm, wingspan 9.0 mm. ***Head***: frons with pure white appressed scales, vertex with long white scales from sides of occiput curving over occiput, further tufts of long white scales from sides of posterior part of occiput pointing posteriorly and inwards, overlaying collar of broad white appressed scales projecting posteriorly from anterior margin of prothorax. Pilifers small with small tufts of bristles; maxillary palps white. Labial palps long (> 2.5× diameter of eye), recurved, second segment substantially longer than third, strongly curved, white, thinly scaled, third segment almost straight, with thin covering of white appressed scales. Haustellum with flat silver scaling on basal portion. Antenna > ¾ length of forewing, bipectinate, scape thickly scaled, white, flagellum with first few flagellomeres white, thereafter dark brown, long dark pectinations covered in white sensillae for over ½ length, reducing at ¾, apical portion filiform. ***Thorax***: white with some iridescence, tegulae long, white; foreleg with femur white, tibia and tarsus brown, moderate tibial epiphysis, mid and hind legs white, moderately broad tuft of long white scales on hind leg. Forewing broad, costa almost straight for most of length, curving inwards towards apex, termen angled sharply inwards, tornus obtusely rounded, white, unmarked except for a very small sub-apical spot consisting of a few broad dark brown scales. Hindwing slightly less broad than forewing, apex pointed, slightly projecting, white, unmarked. Ventrally: forewing with dark brown scaling between costa and Sc on forewing, otherwise with greyish ochreous scaling, particularly along veins, hindwing white.

**Female**. Similar to male. Wingspan 11.0 mm.

***Pre-genital abdomen***. White with small white anal tuft. Narrow patches of tergal spines on T2–T7; T8 and sternites unsclerotised. Apodemes slightly curved; venulae straight.

***Male genitalia***. Uncus short, broad throughout, rectangular, posterior apex slightly bilobed. Gnathos with medial band projecting posteriorly, uniformly curved. Tegumen band broad, strongly arched, lateral extensions of tegumen same length as width of tegumen band. Vinculum long, robust, broad, U-shaped, base projecting some distance beyond base of valvae. Saccus short and broad. Juxta with rounded basal plate, anellus lobes broad and long, reaching beyond costal margin of valva. Valva broad at base, tapering slightly towards posterior apex, costal margin almost straight but angled sharply towards apex, long, curved, sclerotised process at apex of valva, ventral surface with scattered setae near costal margin, ventral margin curved, weak ventral sclerite medially beyond sacculus. Sacculus indistinct, long, and narrow, thickened postero-distal membranous extension small and almost linear. Aedeagus thin and slightly curved.

***Female genitalia*** (Fig. 74). Papillae anales short and very broad. Apophyses anteriores slightly longer than apophyses posteriores. S8 medially emarginate with a row of bristles on emarginate surface. Ostium small and rounded. Antrum short, sclerotised, parallel sided, with small semicircular sclerite medially. Ductus bursae thin, membranous, narrow. Corpus bursae very small, pyriform, without signum.

###### Biology and early stages.

Early stages unknown. Adults found in January, February, and October.

###### Note on Meyrick’s etymology.

Meyrick’s original etymology is as follows: *acrosticta* Meyrick, 1906 – from *acron* (Gr.), the top; and *stiktos* (Gr.), spotted; this species is all white except for a dark pre-apical spot on the forewing.

###### Remarks.

We examined 11 specimens of *P. acrosticta* which were acquired by the NHMUK from Meyrick’s collection. Meyrick’s original description mentions seven specimens, Puttalam and Hambantota, Ceylon in January, February, and October (Pole). Five of the specimens we have examined from Meyrick’s collection, including the lectotype, fit Meyrick’s description of the type material, whereas none of the other specimens we examined fit that description.

##### Ptochoryctis flavalbella sp. nov.

Taxon classificationAnimaliaLepidopteraAutostichidae

4DFC39DE-6236-5211-863F-3AA380B51CD8

https://zoobank.org/50F35F44-EACC-478B-A434-C00D53E685E1

Figs 23, 61 A, B

###### DNA barcode.

BIN: N/A. The sequence fragment obtained of 456 bp (Process ID METAT221-19) was too short for a BIN to be allocated.

###### Type material.

Thailand. ***Holotype*** • ♂, N. Thailand, Chiang Mai, km 24 on Mae Rim to Samoeng Rd, 1100 m, 01.vi.1989, I.J. Kitching leg., fwl 9 mm, specimen no. NHMUK010923181, slide no. NHMUK010316405. ***Paratype***: 1 • ♂ W. Thailand, Uthai Thani Dist., Khao Nang Rum, 400 m, 6–8.vi.1986, Col. M.G. Allen leg., specimen no. NHMUK010219780, slide no. NHMUK010316867.

###### Diagnosis.

Externally, this the only species of *Ptochoryctis* which has uniformly pale yellowish white forewings. It slightly resembles *P. persicotincta*, but that species is paler in colour and slightly smaller. In the male genitalia, the medial plate of the gnathos of *P. flavalbella* has a strongly sclerotised single medial posterior projection. It is the only member of the genus, other than some members of the *anguillaris* group, to which it has little external resemblance, to have this character.

**Figures 23–34.**
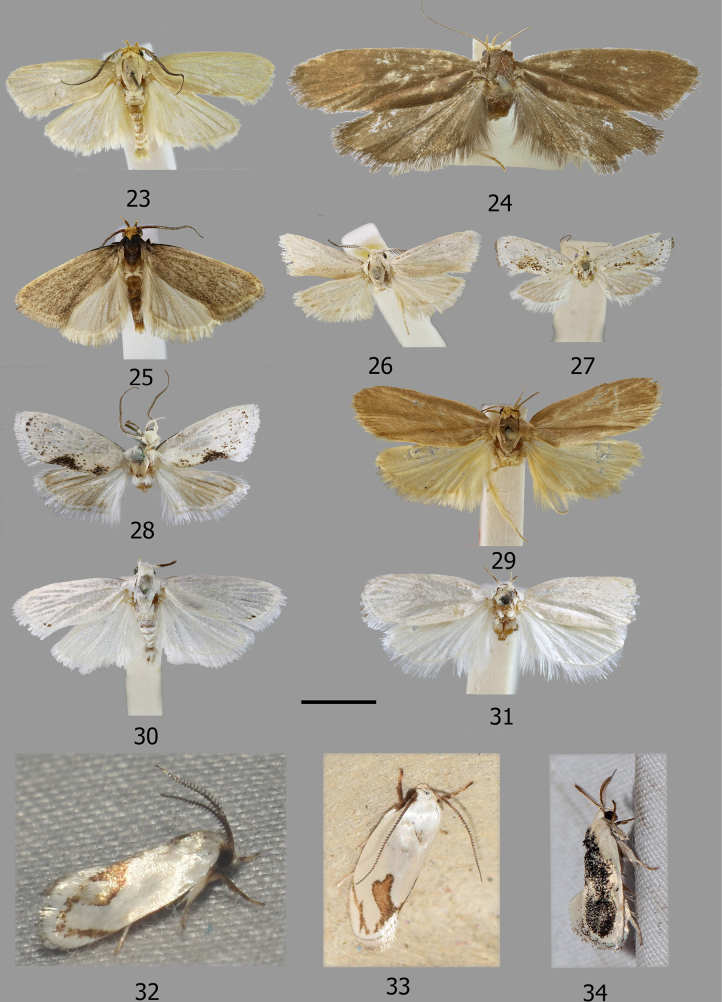
Dorsal and habitus images of *Ptochoryctis* and *Metathrinca* spp. **23**. *Ptochoryctis flavalbella* sp. nov. holotype • ♂; **24**. *P. galbanea* (Meyrick, 1914) lectotype ♀; **25**. *P. ochraceella* sp. nov. holotype • ♂; **26**. *P. scionota* Meyrick, 1906, lectotype • ♂; **27**. *P. simbleuta* Meyrick, 1907, lectotype • ♂; **28**. *P. splendidella* sp. nov., holotype • ♂; **29**. *Metathrinca alma* (Meyrick, 1908), comb. nov., holotype ♀; **30**. *M. inviolata* (Meyrick, 1925), comb. nov., • ♂; **31**. *M. ochrograpta* (Meyrick, 1923), comb. nov., holotype ♀; **32**. *P. sundarbanica* Sterling & Singh, 2025 • ♂; **33**. *P. caputanatis* sp. nov. • ♂; **34**. *P. aff. chalazopa*. Scale bar: 5 mm (**23–31**).

**Male** (Fig. 23). Forewing length 9.0 mm, wingspan 20.0 mm. ***Head***: frons with pale ochreous appressed scales, vertex with tufts of long yellowish brown scales from sides pointing inwards, ruff of long pale ochreous scales from posterior margin of occiput pointing posteriorly overlaying a collar of broad pale ochreous scales on anterior margin of prothorax pointing posteriorly. Pilifers cylindrical with moderate tufts of bristles; maxillary palps not visible. Labial palps long (2.5× diameter of eye), strongly recurved, basal segment with appressed ochreous scales, second segment substantially longer than third, strongly curved, thinly covered in pale ochreous scales, third segment long, almost straight, with appressed pale ochreous scales. Haustellum with base covered in silver scales. Antenna just over ¾ length of forewing, scape yellow ochreous, flagellum dark with dorsal surface ochreous on basal half, bipectinate, pectinations moderately long, covered in short white sensillae for ¾ length, apical portion filiform. ***Thorax***: pale ochreous, tegulae short, pale ochreous; forelegs and mid legs ochreous, broad tibial epiphysis; hind legs with large tuft of long yellowish white scales. Forewing broad, costa slightly arched, apex obtusely rounded, termen slightly angled inwards, tornus obtusely angled, pale yellowish white, wholly unmarked although with thicker patch of dorsal scaling concolorous with rest of forewing. Hindwing as broad as forewing, rounded, pale yellowish white, unmarked. Ventrally: forewing pale ochreous with ochreous scaling between costa and Sc on forewing and along veins; hindwings silver with ochreous scaling along veins.

**Female**. Unknown.

***Pre-genital abdomen***. Pale ochreous, anal tuft pale ochreous. Large patches of tergal spines on posterior half of T2–T7; T8 unsclerotised; sternites weakly sclerotised. Apodemes slightly curved; venulae straight.

***Male genitalia*** (Fig. 61A, B). Uncus short and anteriorly broad, tapering to a narrow point at posterior apex, anterior margin of dorsal surface strongly emarginate. Gnathos with long broad lateral arms, medial plate with large well sclerotised single medial posterior projection. Tegumen band broad, lateral extensions of tegumen same length as width of tegumen band. Vinculum long and robust, U-shaped. Saccus long and broad, projecting significantly beyond base of valvae. Juxta with basal plate U-shaped, anellus lobes broad and curved, terminating level with base of costal margin of valva. Valva broad, tapering postmedially to a pointed apex, costal margin almost straight, ventral margin curved, ventral surface with short, fine, fairly sparse setae, small, weak, ventral sclerite medially, apex of valva with small node. Sacculus indistinct, longer than broad, thickened postero-distal membranous extension broad. Aedeagus slightly curved.

###### Biology and early stages.

Early stages unknown. Adult found in June at 400 and 1100 m.

###### Distribution.

Thailand.

###### Etymology.

*Ptochoryctis flavalbella* sp. nov. – from *flavus* (Lat.), yellow; and *albus* (Lat.), white. This is another unicolourous species but is relatively distinctive as its white ground colour is tinted yellow. The epithet is an adjective in the nominative singular.

##### Ptochoryctis galbanea

Taxon classificationAnimaliaLepidopteraAutostichidae

(Meyrick, 1914)

DC54B5AC-6BF0-54A5-830E-F32FD1CC4E70

Figs 24, 75

Amorbaea galbanea Meyrick, 1914: 778

###### DNA barcode.

N/A

###### Type material.

***Lectotype*** Sri Lanka • ♀, Maskeliya, Ceylon. Green leg. .4.05, specimen no. NHMUK010219668; slide no. JFGC 7703, lectotype designated by Gates Clarke (Clarke 1955b: 493). ***Paralectotype***, • 1♀, Maskeliya, Ceylon. Alston. 2.06., specimen no. NHMUK013700908.

###### Diagnosis.

The large size and uniform dull brown colouration of this species distinguish it from other species of *Ptochoryctis*.

**Male**. Unknown.

**Female** (Fig. 24). Forewing length 14.0–15.0 mm, wingspan 30.0–32.0 mm. ***Head***: Frons (worn) with some evidence of white scaling, vertex (worn) with a few white appressed scales remaining, remains of tufts of long ochreous scales at sides overlaying a few broad flat brown scales on anterior margin of prothorax. Pilifers with one or two bristles visible; maxillary palps not visible. Labial palps long, almost 2.5× diameter of eye, strongly recurved, basal segment ochreous, second segment long, strongly curved, substantially longer than third, thinly scaled yellowish ochreous, third segment long, thin, with appressed ochreous yellow scales. Haustellum with whitish scales on basal portion. Antenna ¾ length of forewing, dark brown, filiform, scape thinly scaled dark brown. ***Thorax***: dark brown, tegulae short, dark brown; foreleg pale greyish brown, tibial epiphysis small, mid and hind legs ochreous, hind legs with remains of tuft of long ochreous scales. Forewing broad, costa gently arched at base, otherwise straight, apex a broad rounded point, termen slightly angled inwards, tornus obtusely angled, uniformly dull brown, unmarked, cilia long, dull brown. Hindwing broad, rounded, same colour as forewing. Ventrally: forewing and hindwing scaled dull brown.

***Pre-genital abdomen***. Patches of tergal spines on posterior parts of T2–T7 (patch on T7 thin and rectangular). Apodemes curved; venulae sinuate.

***Female genitalia*** (Fig. 75). Papillae anales long and strongly setose; apophyses posteriores 1½ × length of apophyses anteriores. S8 with two rounded sclerites, long robust bristles on posterior margin. Ostium narrow and circular. Antrum slightly longer than apophyses posteriores, lightly sclerotised, narrowing anteriorly. Ductus bursae broad, membranous, not clearly differentiated from corpus bursae. Corpus bursae rounded with small triangular signum, strongly sclerotised laterally.

###### Biology and early stages.

Early stages unknown. Three adults found in February to April.

###### Distribution.

Sri Lanka.

###### Note on Meyrick’s etymology.

*galbanea* (Meyrick, 1914) – Galbanum is a gum resin produced from the sap of various species of Apiaceae, particularly from Iran. Galbanum resin can be various colours, but the wing colour of this species resembles its darker forms.

##### Ptochoryctis ochraceella sp. nov.

Taxon classificationAnimaliaLepidopteraAutostichidae

04B91555-DE14-55DF-9965-B96E4D19048B

https://zoobank.org/939A7BA3-AA71-4055-8BBB-C615CC4736B5

Figs 25, 62 A, B, 76, 79, 107

###### DNA barcodes.

BIN, BOLD:ADR5905 (Process IDs METAT050-18, METAT329-24, DEPAL057-20, DEPAL058-20).

###### Type material.

Thailand. ***Holotype*** • ♂; Chiang Mai, Chiang Dao, San Pakia RFD Watershed Station, 1450 m, 28.iv–1.v.1994, I.J. Kitching et al. leg., fwl 9 mm specimen no. NHMUK010923016, slide no. NHMUK010316406, BIN, BOLD:ADR5905. ***Paratypes***: (10♂, 1♀) • 6♂, same collection data as holotype, specimen no. NHMUK010923215, slide no. NHMUK010316462, BIN, BOLD:ADR5905; specimen no. NHMUK010923086, slide no. NHMUK014333411; specimen no. NHMUK013700866; specimen no. NHMUK013700867; specimen no. NHMUK013700868; specimen no. NHMUK013700869; • 3♂ Thailand, Chiang Mai, Doi Chiang Dao, Den Yaa substation, 1450 m, 12-14.iv.1994, I.J. Kitching et al. leg., specimen no. NHMUK010923216, slide no. NHMUK010316463, BIN, BOLD:ADR5905; specimen no. NHMUK013700870; specimen no. NHMUK013700871; • 1♂ NW Thailand Chiang Mai, Doi Suthep-Pui NP, 1440 m, 29.iv-04.v.1988, G.S. Robinson leg., • 1♀, NW Thailand, Chiang Mai, Doi Suthep-Pui NP, 1570 m, GS Robinson leg., specimen no. NHMUK013700442, slide no. NHMUK014332883, BIN, BOLD:ADR5905.

###### Diagnosis.

The unicolourous ochreous brown forewings and pale hindwings distinguish this species from all other species of *Ptochoryctis*.

**Male** (Figs 25, 79). Forewing length 8.0–9.5 mm. ***Head***: frons iridescent gold, vertex with a few long pale orange scales pointing forward from anterior margin, two thick tufts of long dark orange scales from sides of occiput pointing inwards and posteriorly, overlaying in part a collar of flat, narrow, dark brown scales on anterior margin of prothorax pointing posteriorly. Pilifers with moderate tufts of bristles; maxillary palps ochreous. Labial palps long (2.5× diameter of eye), strongly recurved, basal segment brown, second segment same length as third, strongly curved, golden brown with dark brown scaling on basal half of outer surface, third segment straight, with golden brown appressed scales (Fig. 79). Haustellum with silver scaling on basal third. Antennae < ¾ length of forewing, scape with appressed golden brown scales, flagellum with dorsal surface golden brown from base to more than half, otherwise dark, bipectinate, moderate black pectinations covered in short sensillae for ¾ of flagellum, apical portion filiform. ***Thorax***: anterior margin dark brown, otherwise pale brown, tegulae short, anteriorly blackish brown becoming progressively paler, posterior half pale brown; foreleg with femur dark brown, tibia and tarsus black and brown, moderate tibial epiphysis, mid legs brown, hind legs pale ochreous with thin tuft of long ochreous scales. Forewing broad, costa slightly rounded at base, thereafter straight, apex obtusely rounded, termen angled slightly inwards, tornus obtuse, cilia long, very long in tornal area, forewings pale brown, appearing speckled under magnification due to presence of dark tips to many of the scales, unmarked except for a small line of black scales from base of costa to ~ 1/6, cilia concolorous with forewings containing two broad, indistinct lines. Hindwing as broad as forewing, apex rounded, greyish white with some brown scaling in dorsal portion, unmarked, indistinct line in cilia. Ventrally: forewing black along basal portion of costa, otherwise with light grey and brown scaling; hindwing silver with brown scaling on veins.

**Female**. Similar to male.

***Pre-genital abdomen***. Brown, anal tuft pale ochreous. Patches of tergal spines on posterior part of T2–T7; small patch of tergal spines on T8; sternites unsclerotised.

***Male genitalia*** (Figs 62A, 62B, 107). Uncus short, broad throughout, slightly emarginate medially, posterior apex spatulate, anterior margin of dorsal surface weakly emarginate. Gnathos with short broad lateral arms, medial plate with two large, rounded, lateral posterior projections. Tegumen band broad and strongly arched, lateral extensions of tegumen approximately same length as width of tegumen band. Vinculum short, robust, V-shaped, slightly projecting beyond base of valvae. Saccus small. Juxta with circular basal plate, anellus lobes very broad, extending almost as far as costal margin of valva. Valva broad at base, tapering slightly towards apex, costal margin straight, ventral margin gently convex, rounded apically, a patch of sparse setae distally on ventral surface, small weak ventral sclerite medially towards ventral margin. Sacculus indistinct, postero-distal membranous thickening short and broad, distally rounded (Fig. 107). Aedeagus short, slightly curved, Bulbus ejaculatorius over twice length of aedeagus, not coiled.

***Female genitalia*** (Fig. 76). Papillae anales broad with a ring of robust setae anteriorly, apophyses posteriores almost twice length of apophyses anteriores, S8 slightly emarginate posteriorly, emarginate surface strongly sclerotised. Antrum approximately same length as S8, lightly sclerotised, broader posteriorly than anteriorly. Ductus bursae long and membranous with a single coil anteriorly. Corpus bursae pyriform with a narrow signum, strongly sclerotised and pointed on one side.

###### Biology and early stages.

Early stages unknown. Adults found in April and May at or around 1450 m.

###### Distribution.

Northern Thailand.

###### Etymology.

*Ptochoryctis ochraceella* sp. nov. – from *ochraceus* (Lat.), ochreous yellow. This is a reference to the ochreous colour of the forewings of this species. The epithet is an adjective in the nominative singular.

###### Remarks.

Externally this species resembles *Topiris thunbergella* Sterling & Lees, 2025 and an undescribed species of xyloryctid close to *Metathrinca ceromorpha* (Meyrick, 1923), both of which are also known from Northern Thailand. It can be distinguished from the former by the absence of R_3_ in the forewing and the latter by the separation of M_3_ and CuA_1_ in the forewing. It can also be easily distinguished in the male genitalia from these two species by the lack of an articulated saccular process.

##### Ptochoryctis scionota

Taxon classificationAnimaliaLepidopteraAutostichidae

Meyrick, 1906

B7F629CF-2732-5FE2-B5E5-0E556E0E94CE

Figs 26, 63 A, B, 104

Ptochoryctis scionota Meyrick, 1906: 403.

###### DNA barcode.

N/A.

###### Type material.

Sri Lanka ***Lectotype*** • ♂, Puttalam, Ceylon, Pole, .8.04, fwl 6.5 mm, specimen no. NHMUK010219667, slide no. JFGC 7689. Lectotype designated by Gates Clarke (Clarke 1955b: 494). ***Paralectotype*** • ♂, Puttalam, Ceylon, Pole, .8.04 specimen no. NHMUK013700990.

###### Additional material examined.

; • 1♂, Kagalla, Ceylon, CCA, .09, E. Meyrick det., ex Meyrick Coll., specimen no. NHMUK013700991; • 1♂, Putlam 10.94, specimen no. NHMUK013700992.

###### Diagnosis.

Externally the plainest of all the unmarked whitish species of *Ptochoryctis*. It lacks the sprinkling of ochreous scales found in *P. eremopa* and *P. blanchella* and the small, dark pre-apical spot of *P. acrosticta*. In the male genitalia the straight distal margin and the setae at the apex of the valva are diagnostic.

**Male** (Fig. 26). Forewing length 6.5–8.0 mm, wingspan 14.0–17.0 mm. ***Head***: frons with appressed scales, white with some very pale ochreous scaling at outer margins, vertex with small tuft of short, white lamellate scales at base of antennae, moderately sized tuft of whitish cream scales from sides of occiput pointing inwards and the remains of some long white scaling on posterior margin of occiput pointing posteriorly, overlaying a collar of broader pale cream scales from anterior margin of prothorax pointing posteriorly. Pilifers with small bristle tufts. Labial palps long (2.5× length of diameter of eye), strongly recurved, basal segment with small tuft of ochreous scales, second segment longer than third, strongly curved, thinly covered in cream coloured scales, third segment reasonably long, almost straight, thinly covered in appressed white scales. Haustellum with silver scaling on basal portion. Antenna > ¾ length of forewing, scape thickly covered in white scaling reflecting some cream, flagellum dark but dorsal flagellomeres whitish cream for at least ½ length, interspersed with dark ochreous scaling, bipectinate for ~ ¾ of length, pectinations long, covered in short sensillae, apical portion filiform. ***Thorax***: cream coloured, tegulae short, cream coloured; foreleg with femur pale buff, tibia and tarsus brown, thin, moderately long tibial epiphysis, mid and hind legs pale ochreous, hind legs with tuft of fairly short white scales. Forewing broad, costa almost straight, apex slightly angulated, termen moderately angled inwards, tornus very obtuse, cream coloured, iridescent, unmarked except for faint brown line on costa to ~ 1/6, thicker patch of scales along dorsum tinged darker cream, cilia cream. Hindwing as broad as forewing, apex of hindwing slightly pointed, termen of hindwing angled, pale cream with patches of darker scaling around apex, on termen and on dorsal margin. Ventrally: forewings with golden ochreous scaling including along veins; hindwings silvery white.

**Female**. Unknown.

***Pre-genital abdomen***. Cream coloured. Patches of tergal spines on posterior parts of T2–T7; T8 and sternites unsclerotised. Venulae slightly sinuate.

***Male genitalia*** Figs 63A, 63B, 104. Uncus short, anteriorly broad, medially emarginate, posterior apex spatulate, anterior margin of dorsal surface strongly emarginate. Gnathos with short lateral arms, medial plate with concave band projecting posteriorly. Tegumen band broad, arched, lateral extensions of tegumen same length as width of tegumen band. Vinculum very robust, U-shaped, very broad. Saccus short and broad. Juxta with broad basal plate, anellus lobes broad, apically curved, extending almost to costal margin of valva. Valva short and broad throughout, costal and distal margins straight, ventral margin slightly curved, sparse thin setae towards distal margin and apex, weak triangular sclerite from ventral margin. Sacculus broad, indistinct, postero-distal thickened membranous extension long and thin, terminating near apex of ventral sclerite. Aedeagus slightly curved with distal projection.

###### Biology and early stages.

Recorded from *Coffea* (Rubiaceae) and *Camellia sinensis* (L.) Kuntze (Theaceae) (Yunus and Ho 1980). Adults have been found in April and August.

###### Distribution.

Sri Lanka.

###### Note on Meyrick’s Etymology.

*Ptochoryctis scionota* Meyrick, 1906 – from *skia* or *skio* (Gr.), shade; and *noton*, *nota* (Gr.), back. As with a number of species in this group, the dorsal area of the forewing is more thickly scaled than the rest of the forewings, giving an indistinct but slightly darker tinge to the dorsal area of the forewing.

##### Ptochoryctis simbleuta

Taxon classificationAnimaliaLepidopteraAutostichidae

Meyrick, 1907

3D8EC239-6B28-5F89-BA86-819CD96651E5

Figs 27, 64 A, B, 77

Ptochoryctis simbleuta Meyrick, 1907: 150.

###### DNA barcode.

N/A.

###### Type material.

India: ***Lectotype*** • ♂ Gazepore, Assam. A. bred. .3.07, designated as lectotype by Gates Clarke (Clarke 1955b: 494), fwl 5 mm, specimen no. NHMUK010922381, slide no. JFGC 7695. ***Paralectotype*** • ♀, Gazepore, Assam. A. bred. .3.07, specimen no. NHMUK013700888, slide no. NHMUK014333405.

###### Diagnosis.

Externally this species is somewhat similar to *P. splendidella* although the dark marking on the dorsum in this species is more diffuse and less distinct than the corresponding dorsal marking of *P. splendidella*. In the male genitalia, the distal part of the valva of *P. simbleuta* is triangular and pointed whereas the distal part of the valva of *P. splendidella* is rounded Figs 64A, 65A. In the female genitalia, the well-developed lamellae, almost perpendicular to the ostium, are diagnostic (Fig. 77).

**Male** (Fig. 27). ***Head***: frons with cream appressed scales, vertex with white appressed scales overlaid by tufts of long dark cream and white scales from sides, two further tufts of long dark cream and white scales on posterior part of occiput pointing inwards and posteriorly, overlaying collar of broad flat cream scales on anterior margin of prothorax, also pointing posteriorly. Pilifers short and cylindrical with a thick group of bristles; maxillary palps cream. Labial palps long (2.5× diameter of eye), strongly recurved, basal segment pale ochreous, second segment pale ochreous, strongly curved, longer than third segment, thinly scaled pale ochreous, third segment long, thin and pointed with white appressed scales. Haustellum thinly scaled pale ochreous at base. Antenna ¾ length of forewing, shortly bipectinate, scape thickly scaled cream, flagellum with dorsal surface mostly white, short pectinations for ¾ of length, apical portion filiform, short white sensillae on both pectinations and apical portion. ***Thorax***: dark cream with dark brown speckling, tegulae cream with dark brown speckling; foreleg with femur cream, tibia and tarsus brown, moderate tibial epiphysis, mid leg pale ochreous, hind leg with long white scale tuft. Forewing broad, costa gently arched, apex obtusely rounded, termen angled very slightly inwards, tornus very obtusely rounded. Hindwing almost as broad as forewing, apex slightly pointed, white with some irregularly scattered black scales, especially in the disc, a patch of cloudy fuscous suffusion extending from disc beyond middle to tornus, a premarginal series of black dots from 4/5 costa to tornus, cilia white, with fine black median line, apical third grey except above apex and on tornus. Hindwings pale grey, cilia white, with a faint grey median line. Ventrally: forewings scaled brown, hindwings white.

**Female**. Similar to male.

***Pre-genital abdomen***. Patches of tergal spines on posterior parts of T2–T7; T8 and sternites weakly sclerotised. Apodeme slightly curved.

***Male genitalia*** (Fig. 64A, B). (Laterally mounted). Uncus short and broad, substantially tapering towards posterior apex. Gnathos with broad lateral arms, medial plate with large rounded, slightly elongate, lateral posterior projections. Tegumen band broad, arched, lateral extensions of tegumen long, tapering towards vinculum. Vinculum robust, base of vinculum barely projecting beyond base of valvae. Saccus short. Juxta with U-shaped basal plate, anellus lobes broad, extending as far as costal margin of valva. Valva short and broad, tapering to a point at apex, a patch of fine setae distally, weak ventral sclerite medially. Sacculus indistinct, postero-distal thickened membranous extension long, broad at base, slightly tapering distally. Aedeagus almost straight with slight posterior projection.

***Female genitalia*** (Fig. 77). Papillae anales short and broad. Apophyses posteriores slightly longer than apophyses anteriores. Posterior margin of S8 straight with row of bristles posteriorly. Ostium small, circular. Two thin lateral lamellae running almost perpendicular to ostium for 1/3^rd^ length of sclerotised part of antrum. Antrum narrow, parallel sided, sclerotised section slightly shorter than apophyses posteriores. Ductus bursae thin, membranous. Corpus bursae elongate with large elliptical signum medially.

###### Biology and early stages.

According to Meyrick’s original description, the larva is brick red. It feeds beneath a web covered with refuse and pieces of bark on bark of shoots of tea-plant (*Thea* L., now *Camellia* L.), eating right through to the cambium and thus killing the branch or plant. Eight specimens bred. Found in March and April. It has also been recorded as feeding in branches of *Camellia sinensis* (Maxwell-Lefroy & Howlett, 1910).

###### Distribution.

Assam, India.

###### Note on Meyrick’s etymology.

*simbleuta* Meyrick, 1907 – from *simblos* (gr.), a beehive. The cloudy, fuscous suffusion referred to by Meyrick in his description together with the fuscous irroration on the forewings resembles a beehive with bees buzzing round it.

##### Ptochoryctis splendidella sp. nov.

Taxon classificationAnimaliaLepidopteraAutostichidae

8B4EC8FC-8713-5AF7-A788-74A6B3C440A9

https://zoobank.org/8B5FB31A-BF27-4FC3-B8AD-DDEC0B57ED12

Figs 28, 65 A, B

###### DNA barcode.

N/A.

###### Type material.

India. ***Holotype*** • ♂ S. India, Karwar, Castle Rock, 26.v.20, leg. T.R. Bell, specimen no. NHMUK010219676, slide no. NHMUK010316407.

###### Diagnosis.

The differences between this species and *Ptochoryctis simbleuta* Meyrick are set out in the diagnosis of *P. simbleuta* above.

**Male** (Fig. 28). Forewing length 8.0 mm, wingspan 17.5 mm. ***Head***: Frons with thick white broad appressed scaling, vertex with tuft of long white scales on posterior margin of occiput pointing forwards and remains of long white scales pointing posteriorly, overlaying remains of collar of broad flat white scales on anterior margin of prothorax. Pilifers with small tufts of bristles; maxillary palps greyish. Labial palps long (2.5× length of diameter of eye), strongly recurved, principally white, basal segment with a small tuft of golden ochreous scales, second segment substantially longer than third, strongly curved, thickly scaled white with some pale ochreous scaling near base, third segment long, thickly scaled white. Haustellum with silver white scaling on basal portion. Antenna ¾ length of forewing, bipectinate, mainly dark; scape thickly covered in white scales, dorsal surface of basal half of flagellum thickly scaled white, remainder brown, pectinations moderately short, brown, covered with short white sensillae, apical portion filiform. ***Thorax***: pale golden mixed white, tegulae largely missing, white at base; forelegs pale brown, mid legs and hind legs missing. Forewing broad, costa very slightly arched, apex rounded, termen angled inwards, tornus angle almost 90°; iridescent white, a line of dark fuscous scales at base of costa to ~ 1/5, dorsum with fuscous scaling for most of length with a small blotch sub-basally and a larger subquadrate marking post-medially to tornus, a few scattered brown tipped scales post-medially and an interrupted dark brown terminal line, cilia white, very long with a dark brown internal line and remains of a greyish terminal line. Hindwing slightly less broad than forewing, apex rounded, white, scaled brown along veins and with some thin brown scaling between veins, cilia long and white with an indistinct brown line at 1/3. Ventrally, forewings dark brown, hindwings white, scaled brown on veins.

**Female**. Unknown.

***Pre-genital abdomen***. White with white anal tuft. Patches of tergal spines on posterior parts of T2–T7; T8 and sternites unsclerotised. Apodemes slightly curved, venulae straight.

***Male genitalia*** (Fig. 65A, B). Uncus short and broad, medially slightly emarginate, posterior apex bifid, posterior margin with shallow emargination, anterior margin of dorsal surface weakly emarginate. Gnathos with short lateral arms, medial plate with two large rounded, slightly elongate, lateral posterior projections. Tegumen band broad, moderately arched, lateral extensions of tegumen longer than width of tegumen band. Vinculum short and robust, V-shaped, base projecting slightly beyond base of valvae. Saccus short and broad. Juxta with shallow V-shaped basal plate, anellus lobes broad, terminating level with base of costal margin of valva. Valva broad throughout, costal and ventral margins slightly sinuate, apex rounded, fairly sparse fine setae on ventral surface in costal region, extending to apex, long sclerite medially towards ventral margin. Sacculus indistinct, as broad as valva, extending to 1/3 valva, thickened postero-distal membranous extension short and broad. Aedeagus slightly curved, almost uniform width.

###### Biology and early stages.

Early stages unknown. The adult was found in May.

###### Distribution.

South India.

###### Material examined.

Type material.

###### Etymology.

*Ptochoryctis splendidella* sp. nov. – from *splendidus* (lat.), brilliant, magnificent. This is a reference to the richly contrasting markings of the forewings and comparatively large size, for a member of this genus, of this species. The epithet is an adjective in the nominative singular.

##### Metathrinca alma

Taxon classificationAnimaliaLepidopteraXyloryctidae

(Meyrick, 1908) comb. nov.

9450FAC9-51C2-5002-BF75-0ECD1DD47AD3

Figs 29, 66 A, B

Amorbaea alma Meyrick, 1908: 627.
Ptochoryctis alma (Meyrick, 1908).

###### DNA barcode.

N/A.

###### Note.

This species was described from a single female, specimen no. NHMUK010219745, slide no. JFGC 7699, illustrated as a Meyrick type in Clarke 1955b: 492, plate 245, figs 2–2c. There is a male of this species among Meyrick’s materials with the collection data Cuddapah, 4000 ft., .08, WHC. This specimen, NHMUK010929715, slide no. NHMUK010316519, was examined and is illustrated at Figs 29, 66A, B. No DNA sequence was sought from either specimen. In the analyses shown in Figs 2, 4, this taxon appeared within the Xyloryctidae clade but not within the *Ptochoryctis* clade. In this taxon, M_3_ and CuA_1_ are stalked in the forewing and in the male genitalia there is a large, basally curved, strongly setose saccular process and the valva has a setose costal ventral membrane. None of these characters are consistent with *Ptochoryctis*. All of these characters are consistent with the current concept of *Metathrinca*, as it has been expanded, and this taxon is combined accordingly.

##### Metathrinca inviolata

Taxon classificationAnimaliaLepidopteraXyloryctidae

(Meyrick, 1925) comb. nov.

5E627E1C-2A13-503C-9B13-6D4DA3341782

Figs 30, 67 A, B, 112

Ptochoryctis inviolata Meyrick, 1925: 152

###### DNA barcode.

N/A.

###### Note.

This species was described from a single male. However, the NHMUK collection contains six specimens, and the type specimen has not yet been definitively identified. No DNA sequence was sought from any of these specimens. One of the males, specimen no. NHMUK010923083, slide no. NHMUK010316416, was examined and is illustrated at Figs 30, 67A, B, 112. In the analyses shown in Figs 2, 4, this taxon appeared within the Xyloryctidae clade but not within the *Ptochoryctis* clade. In this species, M_3_ and CuA_1_ are stalked in the forewing and in the male genitalia there is a large, basally curved, strongly setose saccular process and the valva has a setose costal ventral membrane (Fig. 112). None of these characters are consistent with *Ptochoryctis*. All of these characters are consistent with the current concept of *Metathrinca*, as it has been expanded, and this taxon is combined accordingly.

##### Metathrinca ochrograpta

Taxon classificationAnimaliaLepidopteraXyloryctidae

(Meyrick, 1923) comb. nov.

42A153BA-60C9-5836-8C16-BE53F3D9E502

Figs 31, 78

Ptochoryctis ochrograpta Meyrick, 1923: 612

###### DNA barcode.

N/A.

###### Note.

This species was described from a single female, specimen no. NHMUK010219704, slide no. JFGC 7702, which was illustrated by Gates Clarke (Clarke 1955b: 495, plate 246, figs 1–1c). This specimen is shown at Figs 31, 78. In the analyses shown in figs 2d 4, this taxon appeared within the Xyloryctidae clade but not within *Ptochoryctis* clade. In the forewing of this specimen, M_3_ and CuA_1_ are stalked, a character which is not consistent with the genus *Ptochoryctis*. This character is, however, consistent with the current concept of *Metathrinca*, as it has been expanded, and this taxon is combined accordingly.

##### Metathrinca perigramma

Taxon classificationAnimaliaLepidopteraXyloryctidae

(Meyrick, 1926) comb. nov.

D7F0EA6C-9318-59FD-9956-A91D76125DAE

Ptochoryctis perigramma Meyrick, 1926: 160

###### DNA barcode.

N/A.

###### Note.

This species was described from a single female in the Sarawak Museum Journal in 1926 (Meyrick 1926: 160). Gates Clarke (Clarke 1955a: 32) stated that, according to the curator of the Sarawak Museum, there are no Meyrick types in that collection and he also failed to find the types of the species described in the Sarawak Museum Journal in Meyrick’s collection and could not give any information regarding their location. The missing Meyrick types from Sarawak were subsequently investigated by Gaden Robinson (Robinson 2004) and are lost. However, Meyrick’s original description notes that vein 3 and 4 (M_3_ and CuA_1_ in Comstock Needham notation) are stalked in the forewing. This character is inconsistent with the genus *Ptochoryctis* but is consistent with the current concept of *Metathrinca*, as it has been expanded, and this taxon is combined accordingly.

**Figures 35–43.**
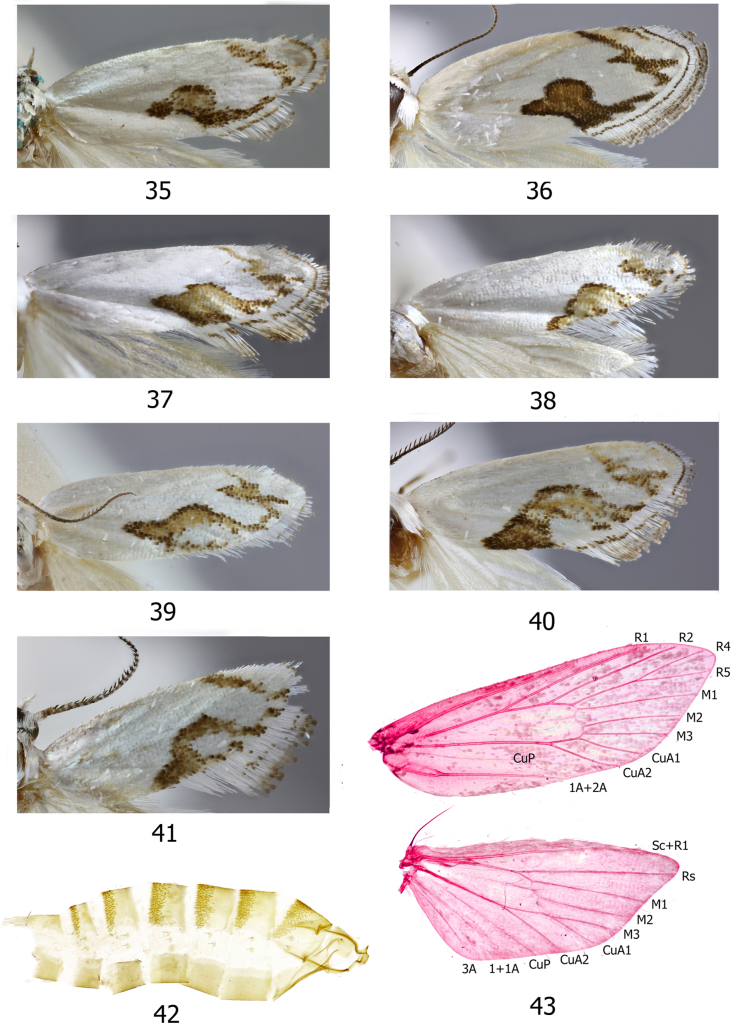
*Ptochoryctis* spp. *anguillaris* group forewings, *Ptochoryctis* pre-genital abdomen and wing preparation. **35**. *P. anguillaris* Meyrick, 1914, lectotype • ♂; **36**. *P. caputanatis* sp. nov., holotype • ♂; **37**. *P. draconella* sp. nov., holotype • ♂; **38**. *P. kitchingi* sp. nov., holotype • ♂; **39**. *P. minimella* sp. nov., holotype • ♂; **40**. *P. robinsoni* sp. nov., holotype • ♂; **41**. *P. sundarbanica* Sterling & Singh, 2025, holotype • ♂; **42**. *P. eremopa* Meyrick, 1894, slide no. NHMUK014332874; **43**. *P. eremopa* Meyrick, 1894, holotype • ♂ slide no. JFGC7683.

**Figures 44–54.**
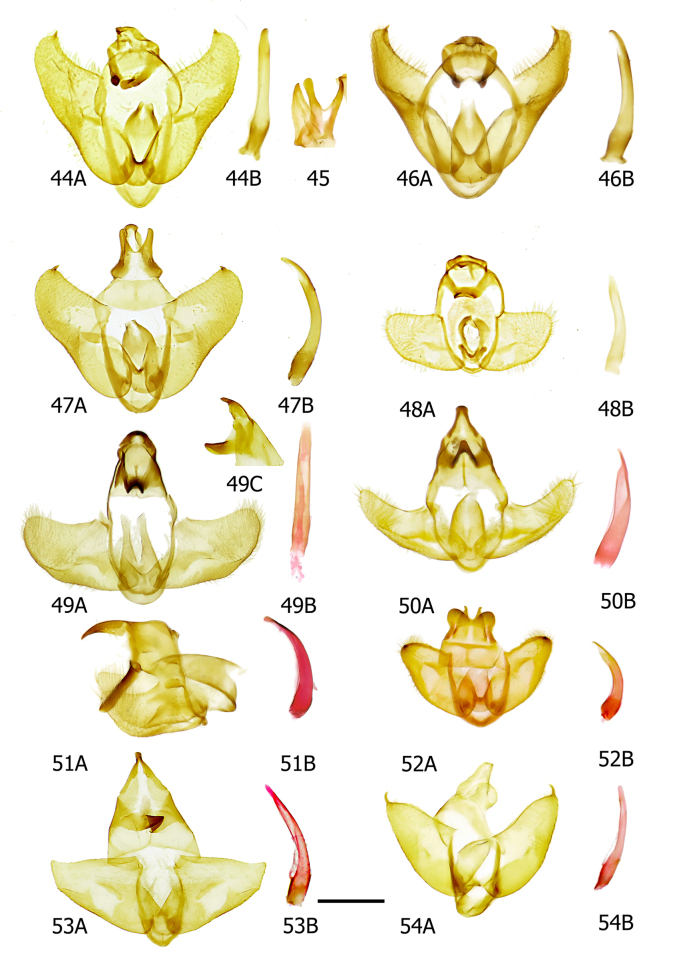
Male genitalia of *Ptochoryctis* spp. **44**. *Ptochoryctis eremopa* Meyrick, 1894, holotype, slide no. JFGC7683; **45**. *P. eremopa* Meyrick, 1894, uncus and gnathos flattened ventrally, slide no. NHMUK014332875; **46**. *P. eremopa* Meyrick, 1894, slide no. NHMUK014332874; **47**. *P. persicotincta* sp. nov., holotype, slide no. NHMUK010316869; **48**. *P. anguillaris* Meyrick, 1914, lectotype, slide no. JFGC 7697; **49**. *P. caputanatis* sp. nov. **A**, **C** holotype, slide no. NHMUK010316363, **B** paratype slide no. NHMUK010316453; **50**. *P. draconella* sp. nov., holotype, slide no. NHMUK014332878; **51**. *P. kitchingi* sp. nov., holotype, slide no. NHMUK014332880; **52**. *P. minimella* sp. nov., holotype, slide no. NHMUK014332882; **53**. *P. robinsoni* sp. nov., holotype, slide no. NHMUK010316364; **54**. *P. sundarbanica* Sterling & Singh, 2025, holotype, slide no. (NZCZSI, H10/Gen/237). Scale bar: 0.4 mm.

**Figures 55–64.**
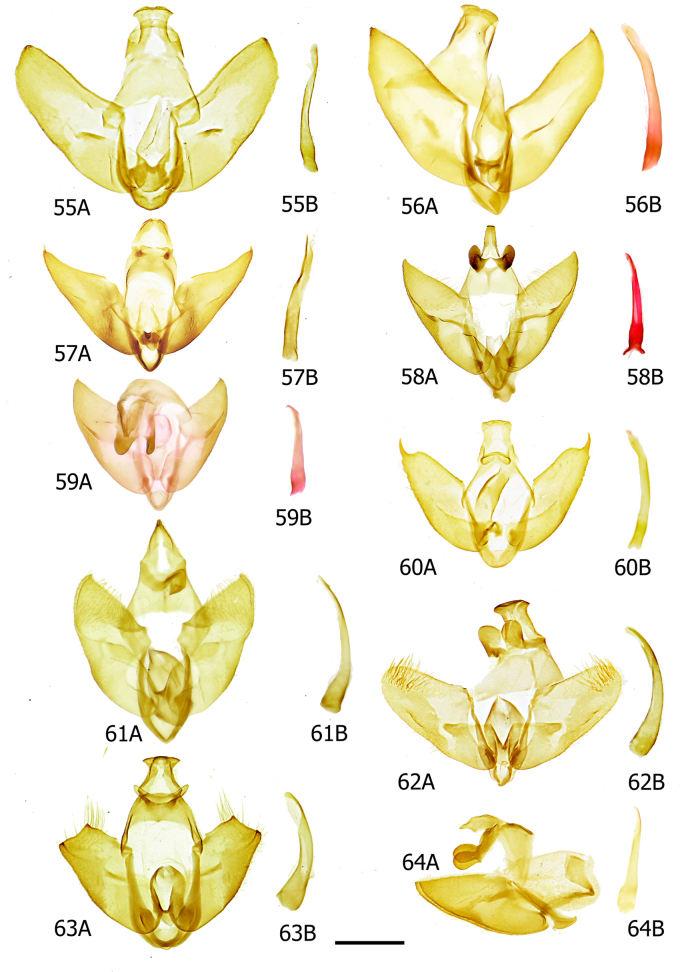
Male genitalia of *Ptochoryctis* spp. **55**. *Ptochoryctis chalazopa* Meyrick, 1920, slide no. NHMUK014332876; **56**. *P. marmorella* sp. nov., holotype, slide no. NHMUK014332877; **57**. *P. corticivora* (Meyrick, 1934), comb. nov., lectotype, slide no. JFGC 7691; **58**. *P. blanchella* sp. nov., holotype, slide no. NHMUK014331356; **59**. *P. fuscilinea* sp. nov., holotype, slide no. NHMUK014332884; **60**. *P. acrosticta* Meyrick, 1906, lectotype, slide no. JFGC 7690; **61**. *P. flavalbella* sp. nov., holotype, slide no. NHMUK010316405; **62**. *P. ochraceella* sp. nov. holotype, slide no. NHMUK010316406; **63**. *P. scionota* Meyrick, 1906 lectotype, slide no. JFGC 7689; **64**. *P. simbleuta* Meyrick, 1907 lectotype, slide no. JFGC 7695. Scale bar: 0.4 mm.

**Figures 65–78.**
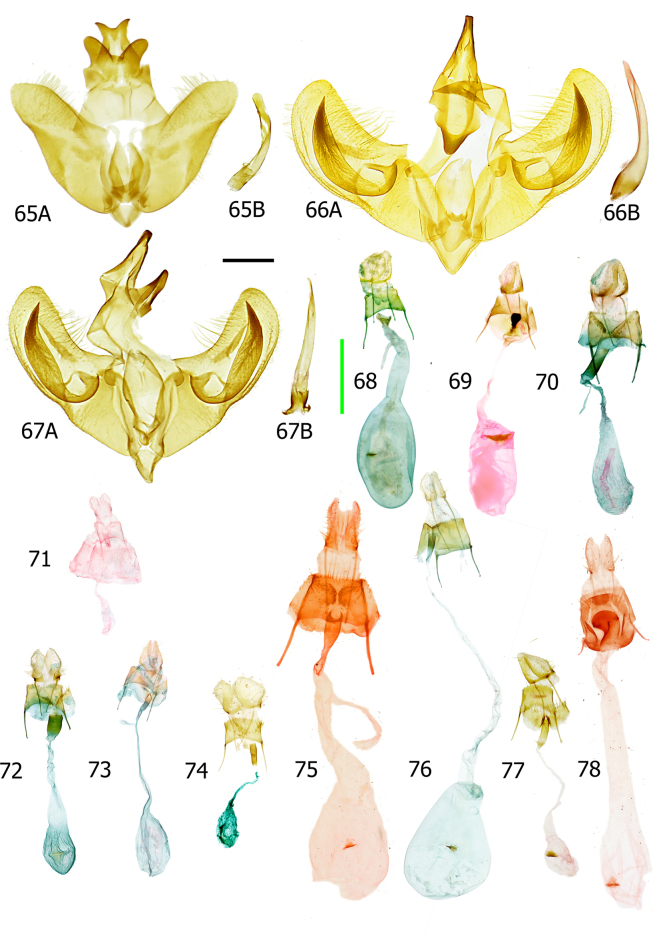
Male and female genitalia of *Ptochoryctis* spp. and *Metathrinca* spp. **65**. *Ptochoryctis splendidella* sp. nov., holotype, slide no. NHMUK010316407; **66**. *Metathrinca alma* (Meyrick, 1908), comb. nov., • ♂ slide no. NHMUK010316519; **67**. *M. inviolata* (Meyrick, 1925), comb. nov., • ♂ slide no. NHMUK010316416; **68**. *P. anguillaris* Meyrick, 1914, ♀ slide no. NHMUK014333410; **69**. *P. caputanatis* sp. nov., ♀ slide no. NHMUK014333413; **70**. *P. chalazopa* Meyrick, 1920, ♀ slide no., NHMUK014333417; **71**. *P. chalazopa* Meyrick, 1920, holotype, ♀ B.M. genitalia slide no. 4880; **72**. *P. corticivora* (Meyrick, 1934), comb. nov., slide no. NHMUK014333414; **73**. *P. fuscilinea* sp. nov., ♀ slide no. NHMUK014333406; **74**. *P. acrosticta* Meyrick, 1906 ♀ slide no. NHMUK014333409; **75**. *P. galbanea* (Meyrick, 1914), lectotype, ♀ slide no. JFGC 7703; **76**. *P. ochraceella* sp. nov., ♀ slide no. NHMUK014332883; **77**. *P. simbleuta* Meyrick, 1907, ♀ slide no. NHMUK014333405; **78**. *M. ochrograpta* (Meyrick, 1923), comb. nov., holotype, slide no. JFGC 7702. Scale bars: 0.4 mm (**65–67**, black), 1 mm (**68-78**, green).

**Figures 79–93.**
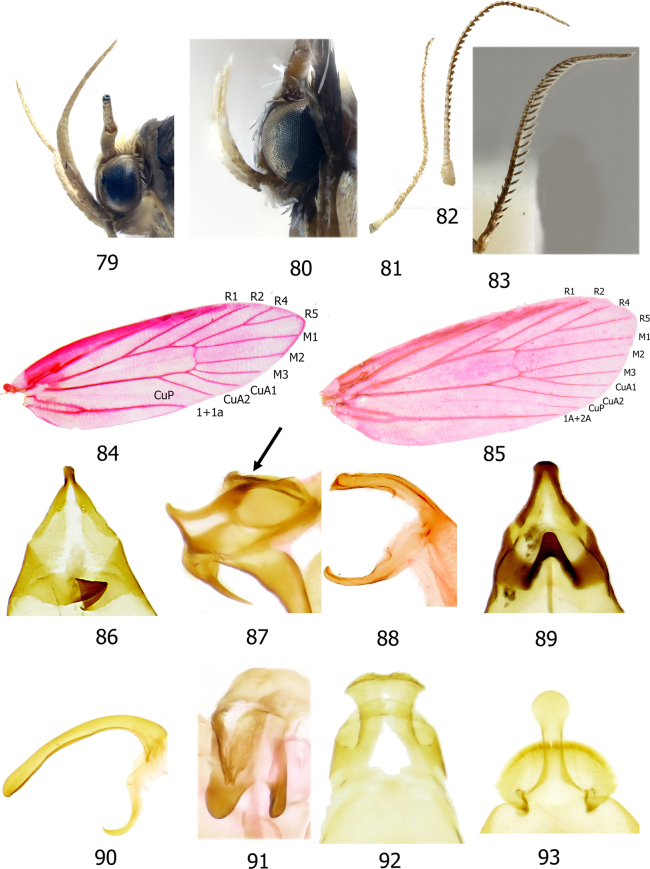
Selected morphological characters. n/n numbers refer to characters/states scored in the morphological analysis (Fig. 4, Suppl. material 1). The first n/n number (where >1) refers to the principal character/state sought to be illustrated. **79**. *Ptochoryctis ochraceella* sp. nov., head, 4/0; **80**. *P. caputanatis* sp. nov., head, 4/1; **81**. *Holcopogon bubulcellus* (Staudinger, 1859), antenna, 6/0; **82**. *P. blanchella* sp. nov., antenna, 6/1; **83**. *P. robinsoni* sp. nov., holotype, antenna, 6/2, 7/1; **84**. *P. caputanatis* wing preparation slide no. NHMUK014333419; **85**. *Metathrinca argentea* Wang et al., 2000, wing preparation, slide no. NHMUK014333418; **86**. *P. robinsoni* sp. nov., holotype, slide no. NHMUK010316364, uncus and gnathos, 24/0, 25/0; **87**. *Ceuthomadarus tenebrionellus* Mann, 1864 slide no. NHMUK014333415, uncus and gnathos, 24/1, 25/5; **88**. *Oegoconia quadripuncta* (Haworth, 1828) B.M. genitalia slide no. 14365, uncus and gnathos, 24/2, 25/1; **89**. *P. draconella* sp. nov., holotype, slide no. NHMUK014332878, uncus and gnathos, 25/0, 24/0; **90**. *Autosticha pelodes* (Meyrick, 1883), holotype, slide no. JFGC9179, uncus and gnathos, 25/1, 24/2; **91**. *P. fuscilinea* sp. nov., holotype, slide no. NHMUK014332884, uncus and gnathos 25/2, 24/0; **92**. *P. chalazopa* Meyrick, 1920, slide no. NHMUK014332876, uncus and gnathos, 25/3, 24/0; **93**. *Periacma ferialis* Meyrick, 1894, holotype, slide no. JFGC7875, uncus and gnathos 25/4, 24/2.

**Figures 94–112.**
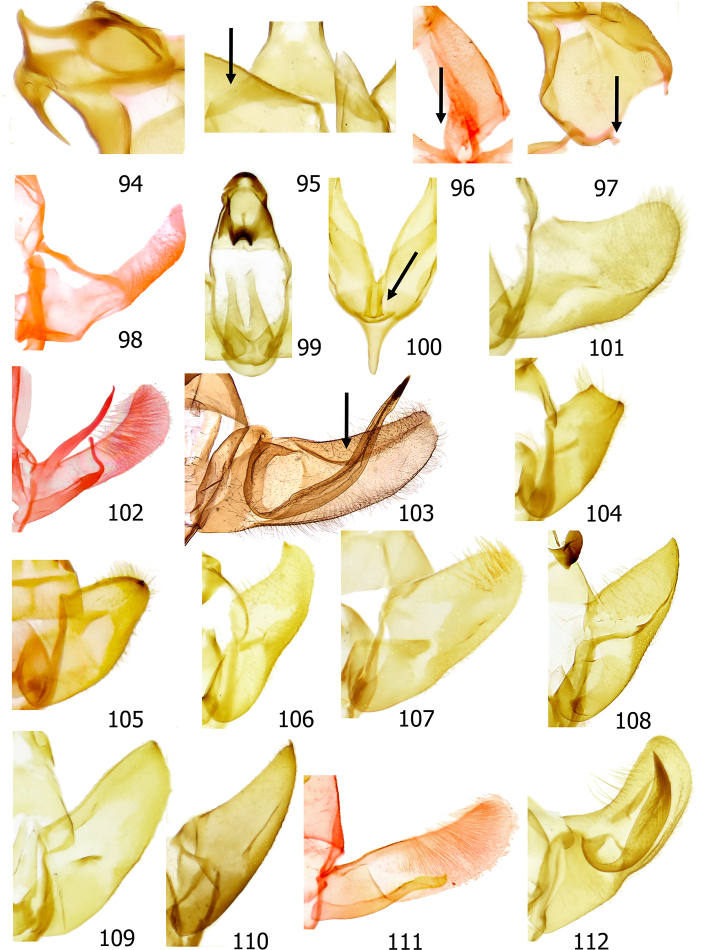
Selected morphological characters. n/n numbers refer to characters/states scored in the morphological analysis (Fig. 4, Suppl. material 1). The first n/n number (where >1) refers to the principal character/state sought to be illustrated. **94**. *Ceuthomadarus tenebrionellus* Mann, 1864 slide no. NHMUK014333415, uncus and gnathos, 25/5; **95**. *Ptochoryctis marmorella* sp. nov., holotype, slide no. NHMUK014332877, tegumen, 28/0, 24/0, 25/3; **96**. *Oegoconia quadripuncta* (Haworth, 1828) B.M. genitalia slide no. 14365, tegumen, 28/2; **97**. *Ceuthomadarus tenebrionellus* Mann, 1864 slide no. NHMUK014333415, tegumen, 28/1; **98**. *Lecithocera nigrana* (Duponchel, 1836) B.M. genitalia slide no. 14081, tegumen bridge, 29/1, 31/0; **99**. *P. caputanatis* sp. nov., holotype, slide no. NHMUK010316363, anellus lobes, 30/0; **100**. *Autosticha modicella* (Christoph, 1882), slide no. NHMUK014333416, anellus lobes, 30/1; **101**. *P. caputanatis* sp. nov., holotype, slide no. NHMUK010316363, valva, 31/1, 34/1 **102**. *Symmoca signatella* Herrich-Schäffer, 1854, B.M. genitalia slide no. 14168, valva, 31/2; **103**. *Metathrinca argentea*Wang et al. 2000, holotype, slide no. L97379, valva, 31/3; **104**. *P. scionota* Meyrick, 1906 lectotype, slide no. JFGC 7689, valva, 34/1, 33/1; **105**. *P. minimella* sp. nov., holotype, slide no. NHMUK014332882, valva, 34/1, 33/1; **106**. *P. eremopa* Meyrick, 1894, holotype, slide no. JFGC7683, valva, 34/1, 33/1; **107**. *P. ochraceella* sp. nov. holotype, slide no. NHMUK010316406, valva, 34/1, 33/1; **108**. *P. blanchella* sp. nov., holotype, slide no. NHMUK014331356, valva, 34/1, 33/1; **109**. *Ptochoryctis chalazopa* Meyrick, 1920, slide no. NHMUK014332876, valva, 34/1, 33/1; **110**. *P. corticivora* (Meyrick, 1934), comb. nov., valva, slide no. NHMUK014333407; **111**. *O. deauratella* (Herrich-Schäffer, 1854), B.M. genitalia slide no. 14269, valva, 35/0, 31/2, 33/0, 36/0, 37/0; **112**. *M. inviolata* (Meyrick, 1925), comb. nov., slide no. NHMUK010316416, valva, 35/1, 31/3, 33/0, 36/1, 37/1.

**Figure 113.**
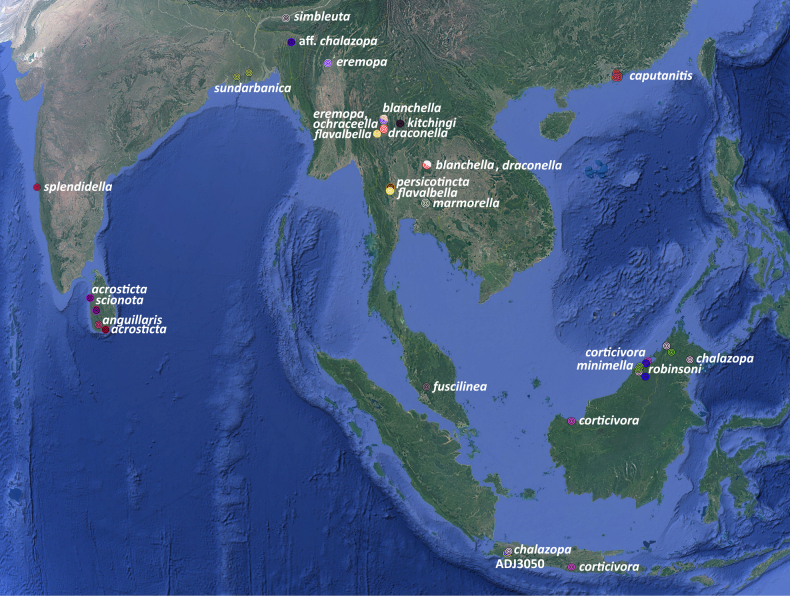
Distribution of species within the genus *Ptochoryctis*. Coloured target symbols represent different species, diagonally overlaid where the same site is represented by different species. Background data courtesy of Google Earth: SIO, NOAA, U.S. Navy, NGA, GEBCO Images Landsat/Copernicus Image IBCAO.

## Discussion

### The genus *Ptochoryctis*

The genus *Ptochoryctis* is both genetically and morphologically diverse. Morphologically, taxa can be strongly marked, as in the *anguillaris* and *chalazopa* groups, or all white with virtually no markings, as in *P. acrosticta* or *P. simbleuta*. The antenna in the male can be serrate, as in the *corticivora* group and some members of the *anguillaris* group, or bipectinate. The labial palps can be short, as in the *anguillaris* group and the *chalazopa* group, or long. In the male genitalia there is no discernible pattern for which taxa have an apical process on the valva, and the shape of the posterior apex of the uncus and the medial plate of the gnathos is variable, as is the shape of the postero-distal thickening of the membrane of the sacculus.

Nevertheless, in each of the analyses shown in Figs 1, 2, 3, 4, *Ptochoryctis* is recovered as monophyletic and each taxon within the *Ptochoryctis* clade exhibits all three morphological synapomorphies (R_3_ absent and M_3_ and CuA_1_ separate or connate, ventral surface of valva with short sparse setae and sacculus with postero-distal membranous thickening appressed to valva), which are identified as important characters in the Results section.

The first of these morphological synapomorphies is a compound character, but in practical terms, the relationship of R_3_, M_3_ and CuA_1_ in the forewing is a very useful diagnostic feature generally in the Asian and Australian Xyloryctidae. The images in Fig. 2 show the diversity in forewing venation of the genera included in our study. The combination of R_3_ present and M_3_ and CuA_1_ separate is characteristic of the Australian xyloryctid taxa. It is a character which is also present in our exemplars from the Autostichidae and the Lecithoceridae. R_3_ present, M_3_ and CuA_1_ stalked is characteristic of *Athrypsiastis* and *Topiris*. R_3_ absent, M_3_ and CuA_1_ stalked is characteristic of *Linoclostis* and almost all *Metathrinca*. Finally, R_3_ absent and M_3_ and CuA_1_ separate or connate is found only in *Ptochoryctis*.

### Family placement of *Ptochoryctis*

*Ptochoryctis* was transferred to the Autostichidae, without explanation, by Hodges (1978). This placement has for some time been open to question. For example, if an exemplar from the genus *Ptochoryctis* is run through Hodges’ key to families and subfamilies of the Gelechioidea (Hodges 1998: 133) it will come out in the Xyloryctidae rather than the Autostichidae.

In each of our analyses, the relevant *Ptochoryctis* clade is recovered within Xyloryctidae. There is no support in any of our analyses for placement of *Ptochoryctis* in the Autostichidae (with the caveat we only have COI-5P for our *Ptochoryctis* exemplars). We therefore transfer *Ptochoryctis* back to the Xyloryctidae, where the genus was originally placed by Meyrick.

### An “Asian White Xyloryctidae” clade?

None of the MrBayes analyses in Figs 1, 2, 3, 4 show support for an “Asian White Xyloryctidae” clade of (*Topiris* + *Athrypsiastis* + “*Ptochoryctis*” + *Linoclostis* + *Metathrinca* + *Ptochoryctis*), although there is an unsupported topology for such a grouping in each of these figures and the character mapping shows one forward nonhomoplasious change and one forward change with homoplasy (or reversal) at the relevant node in Fig. 4. These are: M_3_ and CuA_1_ stalked in forewing (reversed in *Ptochoryctis*) (Fig. 4: character 15, state 1) and antenna of male bipectinate (Fig. 4: character 6, state 2). The male antennae of the Australian Xyloryctidae are variable, both between and sometimes within current genera, but the stalking of M_3_ and CuA_1_ appears to be a very rare character in Xyloryctidae apart from the Asian genera and may be a significant morphological character for this grouping.

### Note on *Athrypsiastis*

The analyses in Sterling et al. (2025) showed no support for an *Athrypsiastis* clade. These analyses examined six taxa, two with limited COI data. In this study we examined four of the same taxa used in the previous study, using the same data. Here, our common combined data analysis supports a clade of *Athrypsiastis penumbrella* + *A. cheesmanae* and our extended combined data analysis supports a clade of (*A. penumbrella* + *A. symmetra* + *A. cheesmanae* + *A. phaeoleuca*). The character mapping shows one forward change with homoplasy or reversal for this clade: aedeagus with single cornutus (Fig. 4, character 38 state 1). Our work has not focused on the Papua New Guinea species of Xyloryctidae and more work needs to be done on this principally New Guinea genus to ascertain its true status and phylogeographic/phylogenetic limits.

### Note on Autostichidae

Our molecular Autostichidae clade is supported with SHaLRT and ABayes measures but only submarginally supported with UFBoot. In our morphological analysis there was submarginal support for a clade encompassing the autostichid exemplars + *Lecithocera nigrana* + *Ceuthomadarus tenebrionellus*, but no support for an Autostichidae clade. However, from our common and extended combined data, a monophyletic Autostichidae is supported. These findings are broadly in line with the results of previous phylogenetic studies.

Kaila’s morphological study of the Gelechioidea (Kaila 2004) found that Autostichidae, *sensu*Hodges 1998 (i.e., consisting of the subfamilies Holcopogoninae, Autostichinae, and Symmocinae), appeared to be paraphyletic, since Glyphidoceridae and Lecithoceridae were also nested within the clade. We did not examine an exemplar of Glyphidocerinae, but our results recovered a similar nesting of our Lecithoceridae exemplars.

The recent study of Yapar et al. (2025) based on 24–1767 genes shows a strongly supported Autostichidae. Our molecular Autostichidae clade is supported by SHaLRT and ABayes measures. We consider that the submarginal support in UFBoot is due to our using fewer exemplars and data than Yapar et al. (2025). The results of our combined data are also in line with the combined molecular and morphological studies of Heikkilä et al. (2014) and Wang and Li (2020), both of which show a strongly supported Autostichidae.

### Further investigation of diversity in *Ptochoryctis*

The genetic and morphological diversification of *Ptochoryctis* referred to above is striking and could be an interesting subject for further investigation. At present it is only in the few instances where pairwise distances among DNA barcodes are relatively small (*P. eremopa* and *P. persicotincta*; *P. chalazopa* and *P. marmorella*) that the relationship between the species is clearly supported by both molecular and combined morphological and molecular data. It remains to be seen whether *Ptochoryctis* is an ancient lineage in which there has been substantial genetic drift, or whether the disparities could be explained by extinction, or whether there still exist substantial numbers of unknown taxa in this genus. In our view, considering that most species are known from few specimens, the latter hypothesis is the most likely. Fig. 113 was compiled from all the spatially disparate collecting events for this genus known to us since the type of *P. eremopa* was discovered in 1888. For almost all species the few such events are scattered over a vast area and over a considerable period of time.

It is evident from iNaturalist, BOLD, and other Asian Lepidoptera websites that there is current interest in this genus and that there are several field workers who feel confident to place specimens to this genus. There is scope for collaboration between such citizen scientists and museum taxonomists. Such collaboration would, however, require retention of specimens, dispatch of those specimens to appropriate depositories for detailed examination, and for workers in those depositories to be able to sequence specimens received. Unfortunately, the current legal and regulatory framework for such a collaboration is almost wholly unfit for purpose.

## Conclusion

There is much more work to be done on this morphologically and molecularly divergent, but still little-known, genus. By gathering all the information which exists on the genus in one place, at least from an examination of NHMUK material, we aim to promote both its further study and the study of the neglected Asiatic taxa of Xyloryctidae.

## Supplementary Material

XML Treatment for Ptochoryctis

XML Treatment for Ptochoryctis eremopa

XML Treatment for Ptochoryctis persicotincta

XML Treatment for Ptochoryctis anguillaris

XML Treatment for Ptochoryctis caputanatis

XML Treatment for Ptochoryctis draconella

XML Treatment for Ptochoryctis kitchingi

XML Treatment for Ptochoryctis minimella

XML Treatment for Ptochoryctis robinsoni

XML Treatment for Ptochoryctis sundarbanica

XML Treatment for Ptochoryctis chalazopa

XML Treatment for Ptochoryctis marmorella

XML Treatment for Ptochoryctis corticivora

XML Treatment for Ptochoryctis blanchella

XML Treatment for Ptochoryctis fuscilinea

XML Treatment for Ptochoryctis

XML Treatment for Ptochoryctis acrosticta

XML Treatment for Ptochoryctis flavalbella

XML Treatment for Ptochoryctis galbanea

XML Treatment for Ptochoryctis ochraceella

XML Treatment for Ptochoryctis scionota

XML Treatment for Ptochoryctis simbleuta

XML Treatment for Ptochoryctis splendidella

XML Treatment for Metathrinca alma

XML Treatment for Metathrinca inviolata

XML Treatment for Metathrinca ochrograpta

XML Treatment for Metathrinca perigramma

## Additional information

### Artificial Intelligence (AI) use

The authors accept full responsibility for the content of the manuscript, including the disclosure of any use of AI.

No AI tools were used in the preparation of this manuscript.

### Author ORCIDs

M.J. Sterling https://orcid.org/0009-0006-5118-2838

D.C. Lees https://orcid.org/0000-0002-7580-8560

### Data availability

All of the data that support the findings of this study are available in the main text or Supplementary Information.

## Supplementary materials

Supplementary material 1 Accession numbers and other data associated with sequenced specimens Data type xlsx https://binary.pensoft.net/file/1723046This dataset is made available under the Open Database License (http://opendatacommons.org/licenses/odbl/1.0/). The Open Database License (ODbL) is a license agreement intended to allow users to freely share, modify, and use this Dataset while maintaining this same freedom for others, provided that the original source and author(s) are credited. Mark J. Sterling, David C. Lees

Supplementary material 2 Character matrix underlying phylogenetic analyses for *Ptochoryctis* and outgroups Data type xlsx https://binary.pensoft.net/file/1723047This dataset is made available under the Open Database License (http://opendatacommons.org/licenses/odbl/1.0/). The Open Database License (ODbL) is a license agreement intended to allow users to freely share, modify, and use this Dataset while maintaining this same freedom for others, provided that the original source and author(s) are credited. Mark J. Sterling, David C. Lees
